# The small GTPase Rit2 modulates LRRK2 kinase activity, is required for lysosomal function and protects against alpha-synuclein neuropathology

**DOI:** 10.1038/s41531-023-00484-2

**Published:** 2023-03-27

**Authors:** Julia Obergasteiger, Anne-Marie Castonguay, Sara Pizzi, Stefano Magnabosco, Giulia Frapporti, Evy Lobbestael, Veerle Baekelandt, Andrew A. Hicks, Peter P. Pramstaller, Claude Gravel, Corrado Corti, Martin Lévesque, Mattia Volta

**Affiliations:** 1 Institute for Biomedicine, Eurac Research, Affiliated Institute of the University of Lübeck, Via Volta 21, 39100 Bolzano, Italy; 2 Department of Psychiatry and Neurosciences, Faculty of Medicine, Université Laval, CERVO Brain Research Centre, 2601 Chemin de la Canardiere, Quebec, QC Canada; 3 Department of Neurosciences, KU Leuven, Herestraat 49 bus 1023, 3000 Leuven, Belgium; 4 Present Address: Department of Cellular, Computational and Integrative Biology—CIBIO, University of Trento, Trento, Italy

**Keywords:** Parkinson's disease, Cellular neuroscience, Cell biology

## Abstract

In Parkinson’s disease (PD) misfolded alpha-synuclein (aSyn) accumulates in the substantia nigra, where dopaminergic neurons are progressively lost. The mechanisms underlying aSyn pathology are still unclear, but they are hypothesized to involve the autophagy-lysosome pathway (ALP). LRRK2 mutations are a major cause of familial and sporadic PD, and LRRK2 kinase activity has been shown to be involved in pS129-aSyn inclusion modulation. We observed selective downregulation of the novel PD risk factor *RIT2* in vitro and in vivo. Rit2 overexpression in G2019S-LRRK2 cells rescued ALP abnormalities and diminished aSyn inclusions. In vivo, viral mediated overexpression of Rit2 operated neuroprotection against AAV-A53T-aSyn. Furthermore, Rit2 overexpression prevented the A53T-aSyn-dependent increase of LRRK2 kinase activity in vivo. On the other hand, reduction of Rit2 levels leads to defects in the ALP, similar to those induced by the G2019S-LRRK2 mutation. Our data indicate that Rit2 is required for correct lysosome function, inhibits overactive LRRK2 to ameliorate ALP impairment, and counteracts aSyn aggregation and related deficits. Targeting Rit2 could represent an effective strategy to combat neuropathology in familial and idiopathic PD.

## Introduction

Parkinson’s disease (PD) is the second most common neurodegenerative disorder affecting 2–3% of people over the age of 65. The disease is characterized by motor symptoms including resting tremor, rigidity, bradykinesia, and postural instability, which originate from the loss of dopaminergic (DA) neurons in the substantia nigra pars compacta (SNc)^1,2^. Around 15% of PD patients have a family history and 5–10% of cases are caused by mutations and alterations in specific genes (e.g., SNCA, LRRK2, Parkin, VPS35)^3^. The vast majority of cases are sporadic and are thought to be caused by a combination of genetic and environmental factors^4,5^. Gene variants in alpha-synuclein (aSyn) and leucine-rich repeat kinase 2 (LRRK2) lead to autosomal dominant PD, mostly presenting intracytoplasmic protein aggregates called Lewy bodies (LBs). LBs are one of the main neuropathological hallmarks of PD and a group of other neurological disorders termed synucleinopathies, and they are mainly composed of oligomeric and fibrillar aSyn^6,7^. Mechanisms underlying the formation and clearance of these inclusions are still under intense investigation. The autophagy-lysosome pathway (ALP) is a conserved and tightly regulated cellular process that is dysregulated in PD and, amongst other cellular processes, likely contributes to the defective clearance of aSyn^8–10^. The ALP leads to lysosomal degradation of long-lived proteins, defective organelles and protein aggregates. Cargo delivery to the lysosome can be achieved by three main pathways: microautophagy, chaperone-mediated autophagy (CMA) and macroautophagy. Macroautophagy, named autophagy herein, involves the formation of double-membrane vesicles, termed autophagosomes that engulf the cargo and finally fuse with the lysosome, where final degradation takes place^11,12^.

Leucine-rich repeat kinase 2 (LRRK2) is a multifunctional protein composed of a catalytic GTPase and kinase core and several protein binding domains^13^. LRRK2 is expressed in different tissues with high expression levels in the lungs, kidneys and white blood cells^14^. In the brain, LRRK2 is expressed ubiquitously and not restricted to neurons^15^. Mutations in the catalytic core have been associated with late-onset familial PD, with the G2019S substitution leading to a 3–4-fold increase in kinase activity and being the most common genetic cause of PD^16^. Cellular roles of LRRK2 are varied and include synaptic transmission, vesicle trafficking and autophagy^17–20^. Numerous studies have reported that mutant LRRK2 alters the ALP, but a common consensus on the specific effects and the impact of LRRK2 mutations on the ALP is currently lacking^21–24^. Moreover, LRRK2 kinase inhibition is beneficial against aSyn neuropathology, suggesting that LRRK2 activity mediates accumulation of pathologic aSyn^25^.

Recent genome-wide association studies (GWAS) identified the locus containing the *RIT2* gene to be associated with increased risk for PD^26–28^. This gene encodes for the small GTPase Rit2, which is mainly expressed in neural tissue^29^. Rit2 belongs to the Ras superfamily of GTPases and is implicated in MAPK-mediated neurite outgrowth^30–32^, calcium signaling^29,30^ and dopamine transporter (DAT) trafficking^33,34^. Furthermore, the knock-down (KD) of Rit2 in male mice altered DAT levels and produced alterations in DA-dependent behaviors^35^. Recently, we reviewed the possible regulation of autophagy by GTPase-MAPK pathways. Since Rit2 modulates the activity of MAPKs (including p38 and ERK1/2), we hypothesized that it could contribute to the regulation of the ALP^36^. Notably, LRRK2 participates to MAPK signaling^37^.

Here, we investigate the effects of altered Rit2 expression on ALP regulation and aSyn pathology and the underlying molecular mechanism using preclinical in vitro and in vivo PD models. Overexpression of Rit2 in G2019S-LRRK2 neuroblastoma cells restored ALP deficits and reduced accumulation of phosphorylated aSyn (pS129-aSyn), phenocopying the effects of pharmacological LRRK2 kinase inhibition that we recently reported^38^. The selective overexpression of Rit2 in DA neurons in the mouse SNc attenuated nigrostriatal neurodegeneration and pS129-aSyn neuropathology induced by virally expressed aSyn. Importantly, we found that Rit2 interacts with LRRK2 and its enhanced expression inhibits both recombinant (in vitro) and endogenous (in vivo) LRRK2 kinase activity. On the other hand, reduced Rit2 levels lead to lysosome alterations, indicating its requirement for correctly functional ALP.

## Results

### RIT2 gene expression is reduced in recombinant LRRK2 cells and in dopamine neurons from idiopathic PD patients

**Fig. 1:**
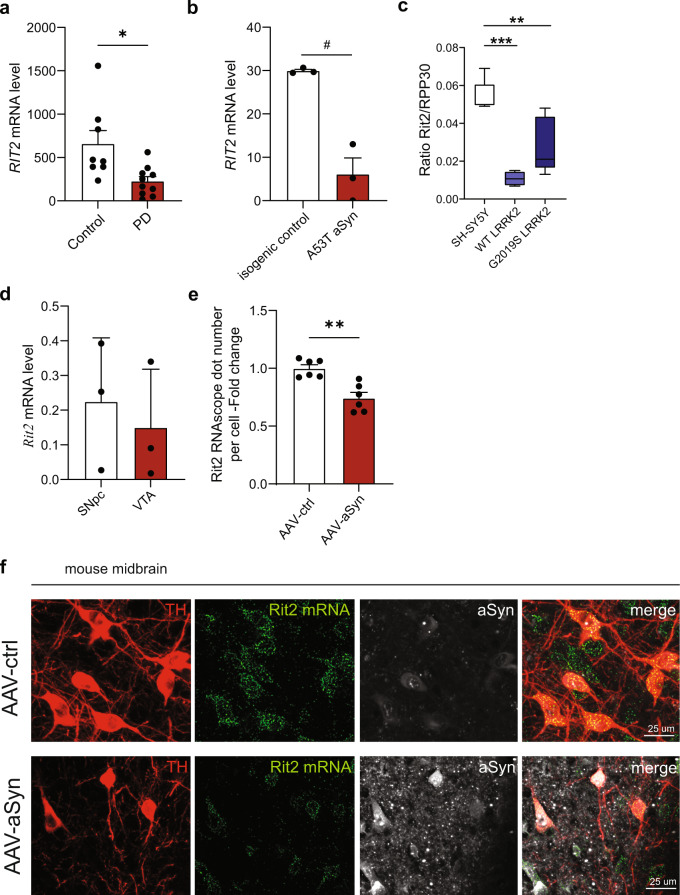
Rit2 is expressed in human and mouse brain, and is reduced in both sporadic PD patients and in cells overexpressing LRRK2. **a** Geo dataset comparing *RIT2* expression levels in DA neurons of the SNc isolated by laser capture microdissection. mRNA levels were reduced by 2.2-fold in DA neurons from sporadic PD patients, when compared to healthy controls (GSE20141, controls = 8, PD = 10). **b** Geo dataset (GSE46798) comparing Rit2 expression in iPSC-derived DA neurons with A53T-aSyn mutation to control mutation-corrected neurons (*n* = 3). **c** Droplet Digital PCR was carried out to assess *RIT2* mRNA levels in recombinant neuroblastoma cell lines. Ratio of *RIT2* mRNA, normalized to RPP30, is reduced in WT and G2019S-LRRK2 cells (*n* = 3). **d** Geo dataset (GSE17542) comparing *Rit2* expression in SNc and VTA from TH-GFP mice (*n* = 3). **e** Quantification of *Rit2* mRNA levels in dopaminergic neurons in the midbrain. Viral aSyn expression decreases *Rit2* mRNA levels (*n* = 6). **f** Fluorescent in situ hybridization (RNAscope®) was employed to show *Rit2* mRNA expression in DA neurons of the mouse SNc and TH and aSyn staining were visualized using immunostaining. Data are represented as median, boxes show the IQ and whiskers show min–max or means ± SEM. *p < 0.05, **p < 0.01 two-tailed Student’s *t* test. ^#^*p* < 0.05 unpaired *t*-test with Welch’s correction after D’Agostino-Pearson test for normal distribution. **p < 0.01, ***p < 0.001, one-way ANOVA followed by Bonferroni’s post hoc test.

Genetic alterations in the *RIT2* promoter region have been associated to PD and are hypothesized to alter expression levels^39^. Accordingly, *RIT2* expression was reduced in the SNc of PD patients^40^. Using publicly available datasets, we analyzed *RIT2* mRNA expression in human and rodent samples. The Geo dataset GSE20141 reports microarray data obtained from laser-capture microdissected SNc DA neurons from idiopathic PD patients and controls^41^. Consistent with literature, we found that *RIT2* expression is downregulated by about 2.2-fold in DA neurons from idiopathic PD patients, when compared to controls (Fig. 1a). Interestingly, *RIT2* mRNA levels in total brain tissue were not changed (GSE7621^42^) (Supplementary Fig. 1a), indicating a specific downregulation of *RIT2* in DA neurons of the SNc. In vitro, *RIT2* mRNA levels were also reduced in DA neurons generated from induced pluripotent stem cells (iPSCs) carrying the A53T mutation in aSyn, when compared to isogenic control cells (published in dataset GSE46798^43^) (Fig. 1b) and in SK-N-SH neuroblastoma cells overexpressing A53T-aSyn (Supplementary Fig. 1b) when compared to naïve SK-N-SH.

To complement these analyses, we measured *RIT2* mRNA levels in WT- and G2019S-LRRK2 overexpressing SH-SY5Y cells using droplet digital PCR (ddPCR). These cell lines have been previously characterized extensively by our group, for LRRK2 expression levels, lysosomal alterations and pS129-aSyn accumulation in G2019S-LRRK2 cells. These phenotypes proved to be dependent on LRRK2 kinase activity as they were ameliorated by the selective LRRK2 kinase inhibitor PF-06447475^38^. Here, we found reduced gene expression in both cell lines, when compared to naïve SH-SY5Y cells (Fig. 1c). In rodents, *Rit2* was reported to be expressed in the brain^44^. The analysis of Geo dataset GSE17542^45^, comparing *Rit2* mRNA levels from laser capture microdissected mouse SNc and VTA did not reveal significant regional differences in *Rit2* mRNA expression (Fig. 1d). To confirm *Rit2* expression in DA neurons of the SNc in our experimental setting, we performed multiplex in situ hybridization (RNAscope® ISH technology) on mouse midbrain sections. Strong *Rit2* mRNA signal was evident in SNc DA (TH+) neurons from control AAV-injected mice, and it was significantly reduced in mice injected with AAV-A53T-aSyn (Fig. 1e, f and Supplementary Fig. 1d). *Rit2* mRNA levels in TH- cells was not affected by AAV-A53T-aSyn (Supplementary Fig. 1c). Together, our results show that *RIT2* is expressed in both mouse and human DA neurons. *RIT2* expression is also reduced in DA neurons from idiopathic PD patients and in different cellular and animal models of PD, suggesting a possible role for Rit2 in both familial and idiopathic PD biology.

### Overexpression of Rit2 rescues ALP deficits in G2019S-LRRK2 cells

It has been extensively reported that both aSyn and LRRK2 affect the autophagic process^46–49^. Furthermore, we recently found that the reduction of pS129-aSyn-positive inclusions in G2019S-LRRK2 cells, following exposure to a LRRK2 kinase inhibitor, depends on functioning lysosomal activity^38^. Since G2019S-LRRK2 cells displayed lower *RIT2* mRNA levels, we decided to reconstitute *RIT2* expression in these cells, using high efficiency nucleofection of a construct containing the human *RIT2* gene. Rit2 protein and mRNA levels after nucleofection are correctly increased (Supplementary Fig. 1e–h). We then looked at specific stages of the ALP to investigate the functionality of the pathway, starting with monitoring the overall autophagic flux.

**Fig. 2:**
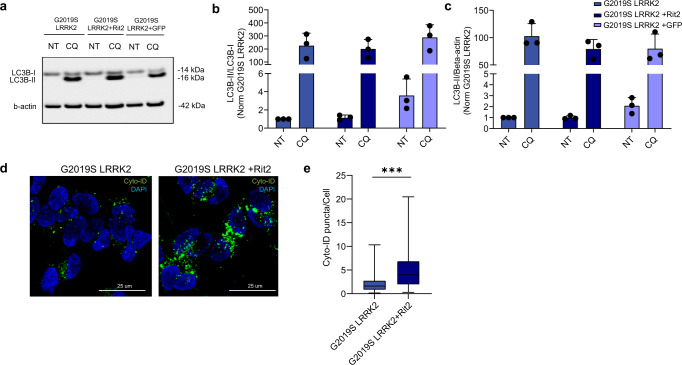
Overexpression of Rit2 does not affect overall autophagic flux. **a** The autophagic flux was assessed in G2019S-LRRK2, G2019S-LRRK2 + Rit2 and G2019S-LRRK2 + GFP cells upon treatment with CQ (100 µM, 3 h) and WB for LC3B. **b** The ratio between LC3B-II and LC3B-I was not different, suggesting no differences in autophagic flux (*n* = 3). **c** Quantification of LC3B levels (normalized to β-actin) indicated no differences upon Rit2 overexpression or CQ-treatment (*n* = 3). **d** CytoID assay was employed to visualize autophagosome and autolysosome distribution. **e** Quantification of CytoID-positive puncta revealed a significant increase in G2019S-LRRK2 cells, when Rit2 was overexpressed (*n* = 4). Data are represented as median, boxes show the IQ and whiskers show min–max or means ± SEM. In imaging experiments, analysis was conducted on 700–1000 cells per group in each experiment. ***p < 0.01, unpaired two-tailed Student’s *t* test.

**Fig. 3:**
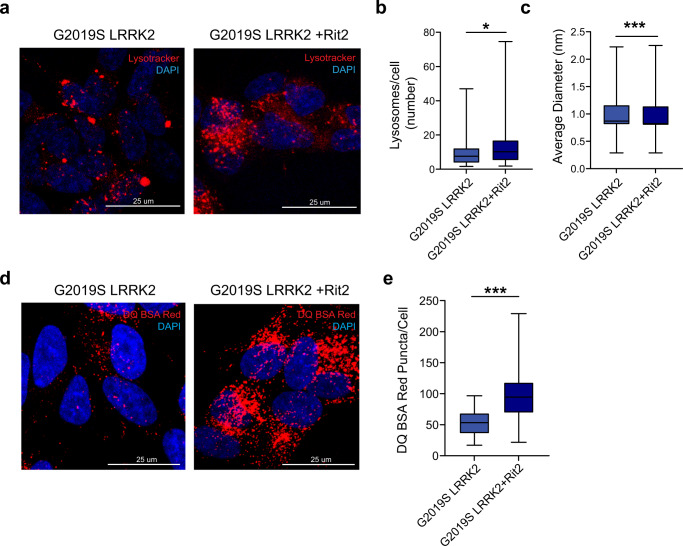
Rit2 overexpression rescues lysosomal morphology and proteolytic activity in G2019S-LRRK2 cells. **a** Cell processing with the Lysotracker Red dye was performed to visualize lysosomes in G2019S-LRRK2 and G2019S-LRRK2 + Rit2 cells. **b** The number of lysosomes per cell was quantified and revealed an increase, when Rit2 was transfected in G2019S-LRRK2 cells (*n* = 3). **c** The average size of lysosomes was assessed, and a significant decrease of the diameter was measured when Rit2 was transfected to G2019S-LRRK2 cells (*n* = 3). **d** The DQ-Red-BSA assay was employed to assess the proteolytic activity of lysosomes. **e** Quantification of DQ-Red-BSA fluorescent spots revealed a significant increase in G2019S-LRRK2 cells, with Rit2 overexpression (*n* = 3). Data are represented as median, boxes show the IQ and whiskers show min–max. In imaging experiments, analysis was conducted on 700–1000 cells per group in each experiment. *p < 0.05, ***p < 0.001, unpaired two-tailed Student’s *t* test.

G2019S-LRRK2 cells were transiently nucleofected with *Rit2* or GFP as a control. After 72 h of overexpression, cellular extracts were prepared and LC3B levels were measured via Western blotting to quantify the dynamics of the autophagic process (Fig. 2a)^50^. The LC3B-II/LC3B-I ratio was not significantly changed between non-transfected G2019S-LRRK2 cells, GFP-transfected cells or cells transfected with Rit2 (Fig. 2b). In parallel, we assessed the LC3B-II/b-actin ratio in the same cellular extracts, which was also not affected by Rit2 overexpression (Fig. 2c). These results indicate that Rit2 overexpression does not affect the overall autophagic flux. Then, we utilized the Cyto-ID assay to visualize autophagosomes and autolysosomes and observed that Rit2 overexpression increased the number of Cyto-ID-stained vesicles (Fig. 2d, e). Given the importance of later fusion steps and our previous results showing impaired lysosome function in the same cell lines, we subsequently evaluated the endpoint effector of the ALP, the lysosome. We investigated lysosomal morphology and number employing the lysosomotropic dye Lysotracker Red, which accumulates in cellular acidic compartments (Fig. 3a). We observed that Rit2 overexpression increases the number of lysosomes (Fig. 3b) and decreases their size (Fig. 3c). The results from LC3B Western blotting and Lysotracker experiments suggest that the increase in the number of Cyto-ID-positive vesicles might derive from an accumulation of autolysosomes/lysosomes and not from an increased production of autophagosomes. Given these morphological alterations, usually coupled to abnormal lysosome function, we measured the proteolytic activity of lysosomes using the DQ-Red-BSA assay. DQ-Red-BSA is degraded by proteases in the acidic environment of the lysosome and an increase of fluorescent spots indicates an enhanced amount of cleaved DQ-Red-BSA, correlating with proteolytic capacity of the cell. The overexpression of Rit2 significantly enhanced the number of fluorescent spots per cell (Fig. 3d, e), indicating an increase in lysosomal proteolysis. Control expression of GFP in G2019S-LRRK2 cells did not modify lysosome morphology or function (Supplementary Fig. 2c–g). Taken together these results reveal that Rit2 does not affect the overall autophagic flux, but rescues the morphological and functional deficits of the lysosomes we previously identified in G2019S-LRRK2 neuroblastoma cells^38^. We replicated some of the above-mentioned experiments in WT-LRRK2 neuroblastoma cells to determine if the effect was specific for the G2019S mutation. Successful nucleofection is again shown by mRNA (Supplementary Fig. 1h) and protein levels (Supplementary Fig. 3h, i). Autophagosome/autolysosome number, measured using the CytoID assay, was not changed when Rit2 was overexpressed in WT-LRRK2 cells (Supplementary Fig. 3a, b). Lysosome number and functionality, measured by Lysotracker Red (Supplementary Fig. 3c–e) and DQ-Red-BSA (Supplementary Fig. 3f, g) staining respectively, were not significantly changed, when Rit2 was overexpressed in these cells. Thus, Rit2 overexpression appears to rescue dysfunctional lysosomes caused by G2019S-LRRK2 while not affecting phenotypes in WT-LRRK2 expressing cells. In addition, we also investigated total aSyn and LRRK2 levels, which were not changed by Rit2 overexpression in the WT-LRRK2 cell line (Supplementary Fig. 3h–k).

### pS129-aSyn positive inclusions in G2019S-LRRK2 cells are reduced by Rit2 overexpression

**Fig. 4:**
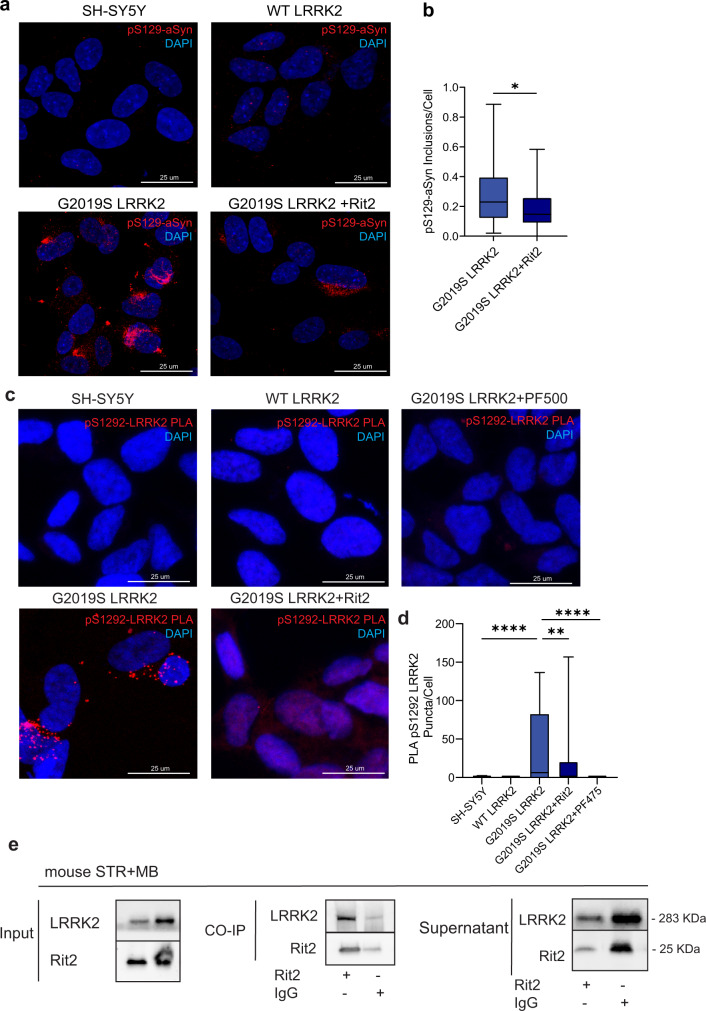
Rit2 overexpression reduces pS129-aSyn positive inclusions, reduces pS1292-LRRK2 and Rit2 and LRRK2 interact directly. **a** Representative images showing pS129-aSyn immunostaining in SH-SY5Y, WT-LRRK2 and G2019S-LRRK2 cells with or without Rit2 overexpression. **b** Quantification pS129-aSyn in cell overexpressing either G2019S-LRRK2 alone or G2019S-LRRK2 with Rit2 (*n* = 4). **c** PLA was used to quantify phosphorylation of Serine 1292 of LRRK2 in neuroblastoma cell lines. **d** The G2019S-LRRK2 mutation increases Serine 1292 phosphorylation, which is reduced by Rit2 overexpression or treatment with PF-475 LRRK2 kinase inhibitor (*n* = 4). **e** CO-immunoprecipitation of LRRK2 using Rit2 antibody in mouse brain lysates. LRRK2 can be precipitated while bound to Rit2 and therefore, the two proteins are physically interacting with each other also at endogenous protein levels in the mouse brain (*n* = 3). Data are represented as median, boxes show the IQ and whiskers show min–max. In imaging experiments, analysis was conducted on 700–1000 cells per group in each experiment. *p < 0.05, **p < 0.01, ****p < 0.0001 two-tailed Student’s *t* test.

A significant proportion of PD patients carrying the G2019S mutation exhibit LB pathology^51,52^ and this mutation has been shown to promote the accumulation of pS129-aSyn in experimental models^25^. SH-SY5Y neuroblastoma cells stably overexpressing G2019S-LRRK2 display inclusion-like structures staining positively for pS129-aSyn, while WT-LRRK2 cells only exhibited diffuse, barely detectable pS129-aSyn staining comparable to naïve cells (Fig. 4a). Enhanced expression of Rit2 showed beneficial effects on different stages of the ALP; therefore, we hypothesized that it might play a role in the degradation of pS129-aSyn. Thus, we decided to investigate the effects of Rit2 on pathological inclusions. G2019S-LRRK2 cells were transiently nucleofected with Rit2 and after 72 h fixed and processed for pS129-aSyn staining. Overexpression of Rit2 significantly reduced the number of objects positively stained for pS129-aSyn per cell (Fig. 4a, b). Control GFP nucleofection did not alter pS129-aSyn inclusion number (Supplementary Fig. 4a, b). Total aSyn levels, measured with Western blotting, were not changed by Rit2 overexpression (Supplementary Fig. 4c, d), indicating a likely specific effect on inclusions of the pathologic form.

### Rit2 overexpression reduces kinase activity of recombinant LRRK2

Our results indicate that Rit2 overexpression in G2019S-LRRK2 cells phenocopies pharmacological LRRK2 kinase inhibition in ALP and pS129-aSyn modulation, as we recently reported^38^. Thus, we hypothesized that LRRK2 and Rit2 could play a role in a common molecular mechanism. We explored the possibility that LRRK2 kinase inhibition could influence *RIT2* mRNA levels, with LRRK2 acting upstream of *Rit2*. We treated G2019S-LRRK2 cells with different concentrations of the LRRK2-selective kinase inhibitor PF-06447475^53^ and we assessed *RIT2* mRNA levels using ddPCR. No difference in *RIT2* gene expression levels were observed upon pharmacological LRRK2 kinase inhibition (Supplementary Fig. 4e). Since LRRK2 kinase inhibition did not affect *RIT2* expression, we explored the hypothesis that Rit2 might directly interact with LRRK2. We first used proximity ligation assay (PLA) for Rit2 and LRRK2 in SH-SY5Y cell lines and observed that overexpressed WT-LRRK2 and Rit2 displayed a robust level of PLA interaction signal. This was strongly reduced in G2019S-LRRK2 cells (Supplementary Fig. 5a, b). To rule out an effect of the different LRRK2 protein expression levels in these cell lines (as we previously reported^54^), we performed the same PLA experiment in HEK293T cells with transient, stoichiometrically comparable expression of Rit2 and LRRK2 (either WT or G2019S). WT-LRRK2 and Rit2 expression again led to a robust PLA signal and the G2019S-LRRK2 mutation decreased the interaction between the two proteins (Supplementary Fig. 5c, d). PLA is a measure of close proximity of two proteins in the cellular environment (<40 nm), but it does not demonstrate physical interaction. Therefore, we confirmed the physical interaction in mouse brain samples (midbrain and striatum pooled) to investigate the interaction of endogenous Rit2 and LRRK2. We observed LRRK2 co-precipitation, when Rit2 was pulled down (Fig. 4e). Furthermore, both Rit2 and LRRK2 levels were reduced in the supernatants of the Co-IP, when compared to the IgG control (Fig. 4e), indicating a depletion due to the pulled-down proteins. This interaction and the phenocopy of LRRK2 inhibition led us to hypothesize that Rit2 overexpression might inhibit LRRK2 kinase activity. LRRK2 phosphorylation was measured using Western blotting at two distinct residues (S935 and S1292), which are responsive to pharmacological LRRK2 inhibition^38,55^. The autophosphorylation of Serine 1292 (pS1292) is a validated readout of LRRK2 kinase activity^55^. We previously found that G2019S-LRRK2 cells harbor a 3–4-fold increase of relative pS1292-LRRK2 when compared to WT-LRRK2 cells^38^. The measurement of LRRK2 phosphorylation by Western blot following overexpression of *RIT2* revealed a significant decrease in the ratio of pS1292-LRRK2/LRRK2 (Supplementary Fig. 6a, b). Differently from LRRK2 kinase inhibitor treatment, pS935-LRRK2 levels were not decreased, but rather significantly increased (Supplementary Fig. 6c, d). Moreover, total LRRK2 levels in G2019S-LRRK2 cells overexpressing Rit2 were also increased (Supplementary Fig. 6e). This effect is distinct from LRRK2 kinase inhibitors, which have been shown to reduce total LRRK2 through protein destabilization likely due to dephosphorylation at the S935 residue^56^. Consistent with a lack of S935 dephosphorylation with Rit2 overexpression, our results indicate that Rit2 does not impair LRRK2 protein stability. We further measured pS1292-LRRK2 levels also using PLA to enhance the specificity and sensitivity of the analysis, as previously validated^57^. Naïve SH-SY5Y and WT-LRRK2 cells displayed negligible levels of pLRRK2 PLA signal, consistent with a very low basal activation of LRRK2^55^, whereas G2019S-LRRK2 cells displayed a dramatic increase in PLA signal (Fig. 4c). The overexpression of Rit2, in line with the western blot data, produced a significant reduction of pS1292-LRRK2 PLA signal. In order to validate the assay, we used pharmacological inhibition of LRRK2 as positive control, which consistently completely abolished the PLA signal (Fig. 4c, d). In summary, we show that Rit2 directly interacts with LRRK2 in the cell and leads to decrease of S1292-LRRK2 phosphorylation, thus limiting LRRK2 kinase activity.

### Enhanced Rit2 expression in the mouse midbrain counteracts aSyn-dependent deficits and DA neuron loss

**Fig. 5:**
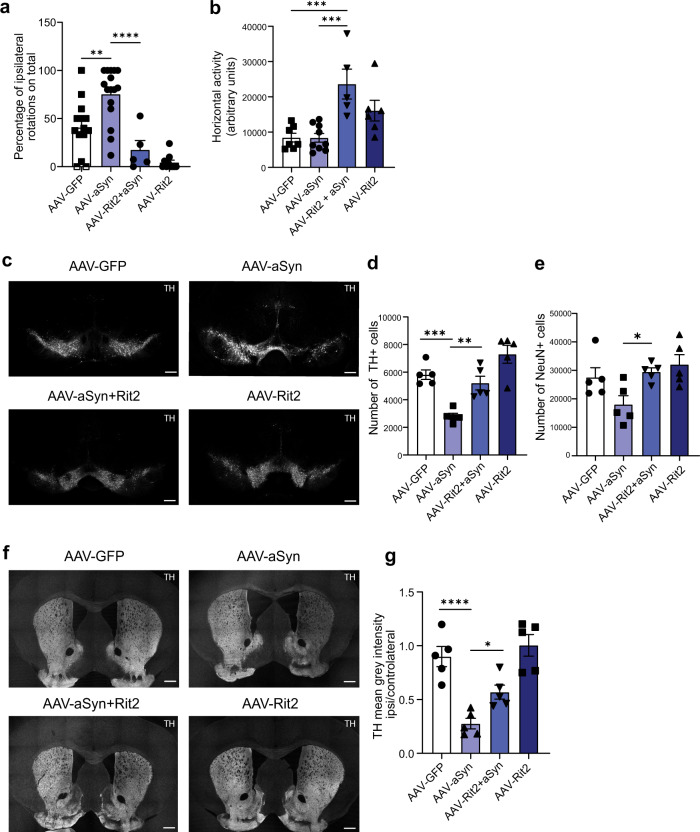
Enhanced Rit2 expression in the mouse midbrain counteracts aSyn-dependent deficits and DA neuron loss. **a** Overexpression of A53T-aSyn increases the number of ipsilateral rotations and co-injection with AAV-Rit2 or injection of AAV-Rit2 alone increases the number of contralateral rotations. **b** Overexpression of Rit2 alone or with aSyn significantly increases horizontal activity in the open field (*n*^GFP^ = 7, *n*^aSyn^ = 9, *n*^aSyn + RIT2^ = 8, *n*^RIT2^ = 6 for behavioral tests). **c** IHC for TH was used to count dopaminergic neurons in the midbrain. **d** Overexpression of A53T-aSyn alone induces a significant loss of DA neurons, which is attenuated by co-injection of AAV-Rit2. **e** A53T-aSyn overexpression tends to reduce the number of NeuN+ cells in the ipsilateral SNc and concomitant overexpression of Rit2 significantly preserves NeuN+ cells (5 animals/group). **f** IHC for TH was used to measure the density of DA projections in the striatum. **g** Overexpression of A53T-aSyn decreases the number of TH+ axons in the striatum, which is attenuated by the co-injection of AAV-Rit2 (*n* = 5/group for IHC experiments). Data represented as mean ± SEM, *p < 0.05, **p < 0.01, ***p < 0.001, ****p < 0.0001, one-way ANOVA followed by Bonferroni’s post hoc test. Scale bar = 500 um.

To determine if the beneficial effects of Rit2 could also be translated in vivo, we modeled PD pathology in mice by unilaterally injecting an Adeno-Associated virus 2/9 (AAV) expressing the human A53T-aSyn (herein AAV-A53T-aSyn). We used DAT-Ires-Cre mice that specifically express the Cre recombinase in DAT-expressing neurons^58^. This allowed us to manipulate gene expression in a neuron-specific manner, as Rit2 is specifically downregulated in DA neurons from PD patients (Fig. 1a). The AAV-mediated overexpression of aSyn in the mouse midbrain has previously been shown to induce progressive SNc neuron loss, aSyn pathology and motor deficits^59^. Sixteen weeks following overexpression of A53T-aSyn (in combination with AAV-GFP), we observed motor abnormalities in the cylinder, open field and amphetamine tests. Mice expressing A53T-aSyn displayed an ipsilateral rotation phenotype (Fig. 5a and Supplementary Fig. 7b) and a preferential use of the ipsilateral forepaw (Supplementary Fig. 7a), but no changes in the horizontal activity in the open field (Fig. 5b). In addition, A53T-aSyn overexpression induced a loss of TH-positive neurons in the midbrain (Fig. 5c–e) coupled to a loss of striatal DA terminals (Fig. 5f, g), mimicking the progressive nigrostriatal degeneration observed in PD. Overexpression of A53T-aSyn also tended to decrease the number of NeuN-positive cells in the ipsilateral SNc (Fig. 5e). To further ensure that overexpression of A53T-aSyn did not cause a downregulation of TH expression without cell loss, we counted the percentage of double-positive cells for GFP and TH in the ipsilateral SNc of AAV-GFP and AAV-GFP + A53T-aSyn injected mice. Both groups showed a percentage of double-positive cells of around 90%, suggesting that A53T-aSyn overexpression did not alter TH expression in dopaminergic neurons (Supplementary Fig. 7c). Mice were co-injected with AAV-A53T-aSyn and the Cre dependent AAV-CAG-Flex-*Rit2*-EGFP (AAV-Rit2) or AAV-CAG-Flex-EGFP (AAV-GFP) control, to achieve overexpression selectively in DA neurons. This strategy ensured that the observed phenotypes would be attributed to the effect of Rit2 in this neuronal population. Rit2 overexpression was confirmed by Western blotting of ventral midbrain protein lysates (Supplementary Fig. 8c). The loss of TH-positive neurons in the SNc (Fig. 5d) and of DA terminals in the striatum (Fig. 5g) were greatly attenuated by the co-expression of *Rit2*, when compared to GFP control, and also significantly preserved NeuN+ cells in the ipsilateral SNc (Fig. 5e). Overexpression of Rit2 alone did not cause alterations to midbrain neuron number nor striatal terminal density, but interestingly, strongly promoted locomotor activity. We observed a marked increase in horizontal activity (Fig. 5b) in the open field, along with an increased number of contralateral rotations in the cylinder test (Fig. 5a).

### Enhanced Rit2 expression reduces pS129-aSyn load and total aSyn levels in the mouse midbrain

**Fig. 6:**
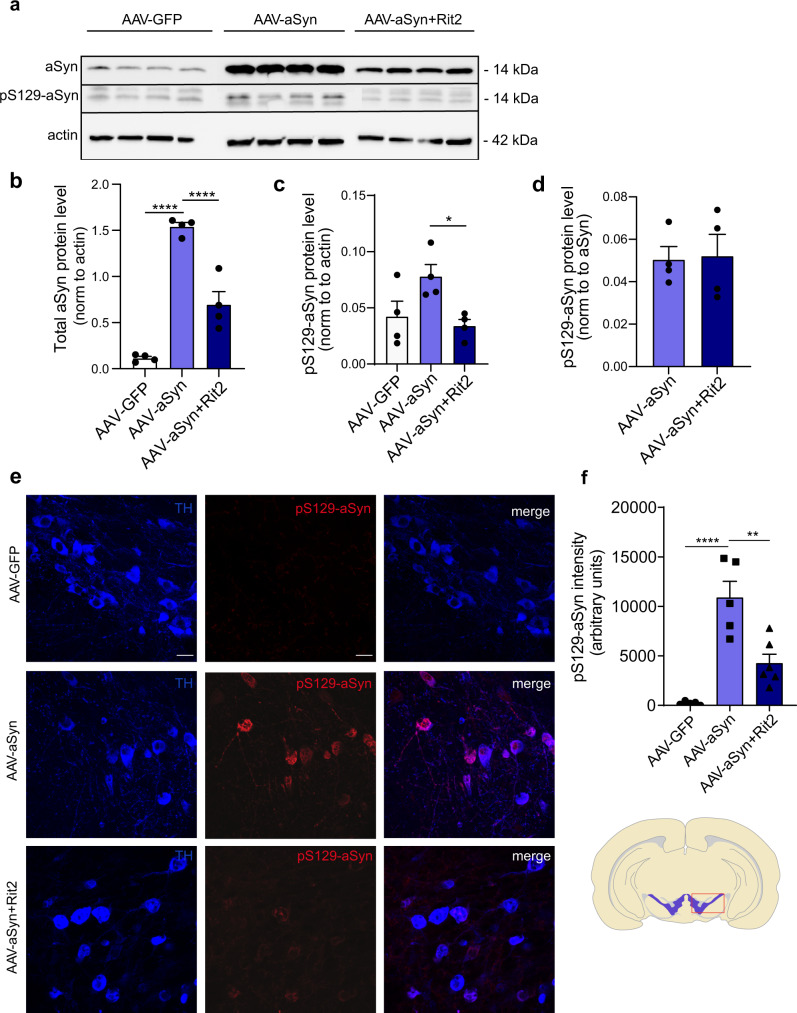
Enhanced Rit2 expression reduces total aSyn and pS129-aSyn levels. **a** Total aSyn levels in mice injected with AAV-A53T-aSyn alone or in combination with AAV-Rit2 were assessed by blotting for total and phosphorylated aSyn and β-actin. **b** Quantification of total aSyn levels, normalized on β-actin (*n* = 4). Co-injection of AAV-Rit2 significantly reduces aSyn levels in the ipsilateral side. **c** Quantification of pS129-aSyn levels, normalized on β-actin (5 animals/group). Co-injection of AAV-Rit2 significantly reduces pS129-aSyn levels in the ipsilateral side. **d** Quantification of pS129-aSyn levels, normalized on total aSyn. Co-injection of AAV-Rit2 does not alter pS129-aSyn/aSyn ratio. **e** IHC staining of pS129-aSyn and TH in the midbrain of AAV-GFP, AAV-A53T-aSyn and AAV-A53T-aSyn + AAV-Rit2 injected mice. **f** Quantification of pS129-aSyn intensity. AAV-A53T-aSyn injection significantly increases the intensity of pS129-aSyn signal, which is reduced by the co-injection of AAV-Rit2 (5 animals/group). Scale bar = 20 um. Data represented as mean ± SEM. *p < 0.05, **p < 0.01, ***p < 0.001, ****p < 0.0001, one-way ANOVA followed by the Bonferroni’s post hoc test.

Viral overexpression of A53T-aSyn in the mouse SNc also led to a significant increase of total aSyn protein levels measured by Western blotting (Fig. 6a). The concomitant overexpression of Rit2 produced a reduction of total aSyn, when compared to AAV-GFP co-injection (Fig. 6b). Importantly, viral A53T-aSyn overexpression significantly increased the burden of pS129-aSyn in DA neurons, when compared to AAV-GFP-injected animals (Fig. 6d). The co-injection of AAV-Rit2 significantly reduced pS129-aSyn levels by Western blotting (Fig. 6c) and immunostaining (Fig. 6d, e) in midbrain DA neurons. This indicates that enhanced Rit2 expression counteracts pS129-aSyn accumulation not only in recombinant neuroblastoma cell lines, but importantly also in an in vivo PD mouse model, providing effective neuroprotection in a non-LRRK2 modeling paradigm. In addition, we investigated the autophagic flux in tissue using Western blotting for LC3B and could not detect any alteration in the LC3B-II/LC3B-I ratio among the experimental groups (Supplementary Fig. 8a, b). This is consistent with the results obtained in the G2019S-LRRK2 cells, where the overall autophagic flux was not altered by Rit2 overexpression.

### Rit2 overexpression prevents aSyn-induced hyperactivation of endogenous LRRK2 kinase

**Fig. 7:**
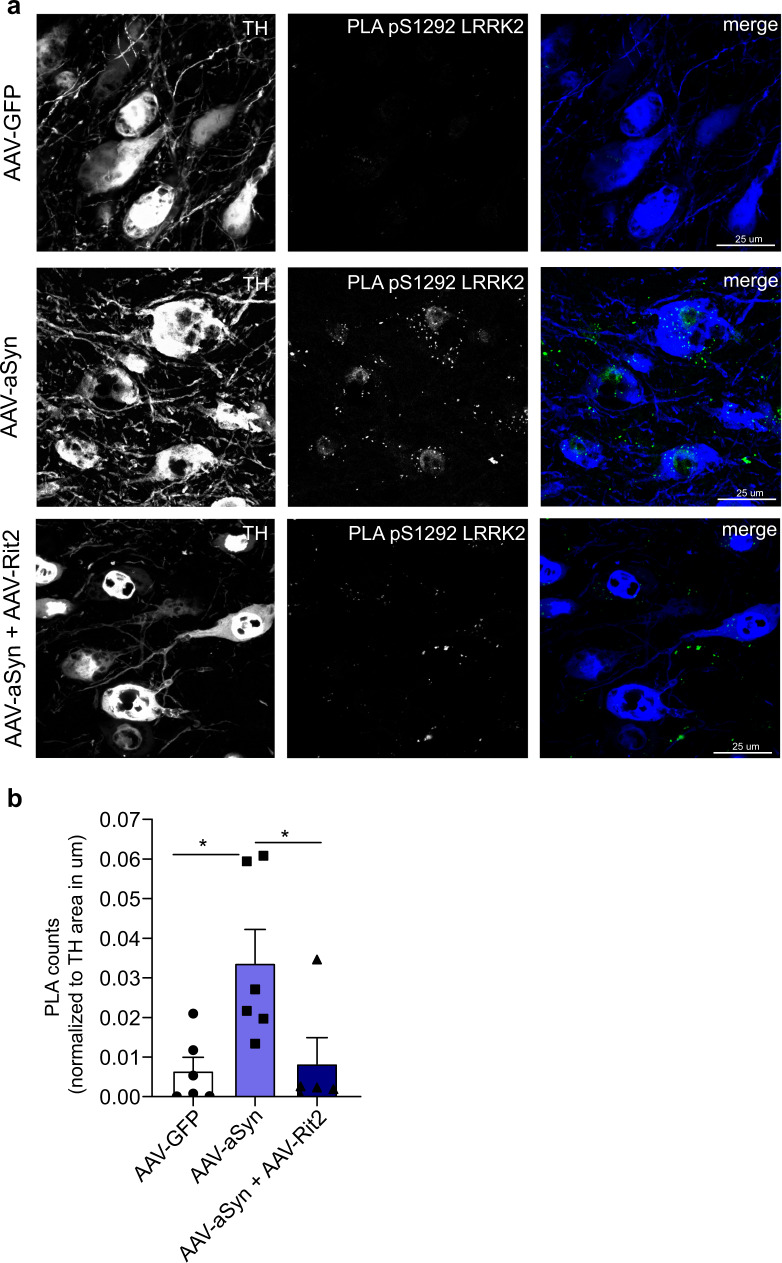
In vivo aSyn overexpression increases endogenous LRRK2 activity, which is prevented by Rit2 co-expression. **a** PLA analysis of AAV-GFP, AAV-A53T-aSyn and AAV-A53T-aSyn + AAV-Rit2 injected mice in TH-positive neurons in the SNc. **b** Quantification of PLA counts in TH-positive neurons shows a significant increase of endogenous LRRK2 kinase activity with AAV-A53T-aSyn injection. The increase is completely prevented by co-injection of AAV-Rit2 with AAV-A53T-aSyn (AAV-GFP = 6 animals, AAV-A53T-aSyn = 6 animals, AAV-A53T-aSyn + AAV-Rit2 = 5 animals). Data represented as mean ± SEM. *p < 0.05, one-way ANOVA followed by Bonferroni’s post hoc test.

We show that viral co-expression of Rit2 with A53T-aSyn in the mouse rescues neuronal loss and aSyn accumulation. In a recent study, viral overexpression of aSyn in rats led to increased kinase activity of endogenous LRRK2, observed as well in the midbrain of idiopathic PD patients, in the absence of any LRRK2 mutation^60^. Here we report that Rit2 reduces phosphorylation levels of S1292-LRRK2 in recombinant neuroblastoma cells (Fig. 4c, d) and is neuroprotective in animals with no LRRK2 mutations (Fig. 5), therefore we assessed the impact of Rit2 on S1292 autophosphorylation in the mouse DA neurons. The validated pS1292-LRRK2/total LRRK2 PLA method was employed in mouse brain sections (Fig. 7a), to increase specificity and decrease background with respect to standard IHC staining^57^. The injection of AAV-A53T-aSyn induced a strong increase in the number of PLA dots, indicating a significant enhancement of endogenous LRRK2 phosphorylation at S1292 and thus an increase in kinase activity. Rit2 co-expression completely prevented the overactivation of endogenous LRRK2 that was elicited by A53T-aSyn (Fig. 7b). Importantly, total LRRK2 levels were not changed in any of the conditions analyzed (Supplementary Fig. 8c, d), supporting a specific kinase-regulating effect. In summary, viral Rit2 overexpression in the mouse SNc DA neurons conferred neuroprotection and reduced aSyn neuropathology through prevention of endogenous LRRK2 overactivation. However, the effects on lysosome function were observed upon Rit2 overexpression, and thus we set out to explore whether Rit2 has a physiological role in the ALP.

### Rit2 is required for correct lysosome function in cells and neurons

**Fig. 8:**
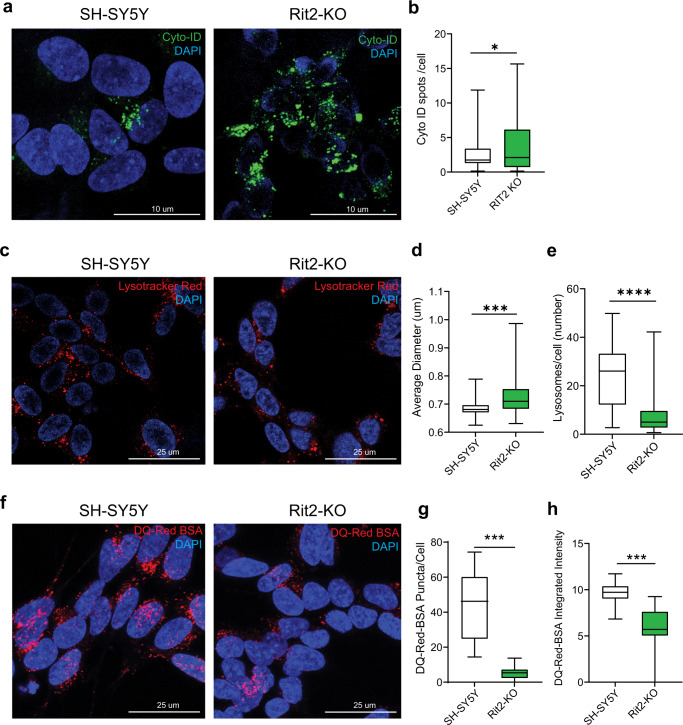
Lysosomal morphology and proteolytic activity are altered in Rit2 KO neuroblastoma cells. **a** CytoID assay was employed to visualize autophagosome and autolysosome distribution. **b** Quantification of CytoID-positive puncta revealed a significant increase in Rit2-KO cells (*n* = 3). **c** Cell processing with the Lysotracker Red dye was performed to visualize number and size of lysosomes. **d** The number of lysosomes per cell was quantified and revealed a decrease, with Rit2 KO (*n* = 6). **e** The average size of lysosomes was assessed, and a significant increase of the diameter was measured in Rit2-KO cells (*n* = 6). **f** The DQ-Red-BSA assay was employed to assess the proteolytic activity of lysosomes. **g**, **h** Quantification of DQ-Red-BSA fluorescent spots revealed a significant decrease of number and intensity in Rit2-KO cells (*n* = 3). Data are represented as median, boxes show the IQ and whiskers show min–max. In imaging experiments, analysis was conducted on 700–1000 cells per group in each experiment. *p < 0.05, ***p < 0.001, ****p < 0.0001 unpaired two-tailed Student’s *t* test.

**Fig. 9:**
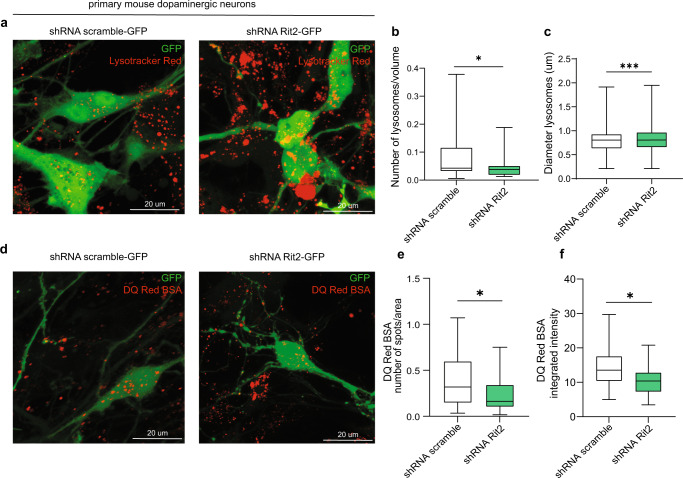
Rit2 KD leads to abnormal lysosomal morphology and proteolytic activity in primary dopaminergic neurons. **a** Lysosomes were visualized with the Lysotracker Red dye in neurons. **b** The number of lysosomes per cell was quantified and revealed a decrease when Rit2 levels were reduced (*n* = 3). **c** The average size of lysosomes was assessed, and a significant increase of the diameter was measured upon Rit2 KD (*n* = 3). **d** The DQ-Red-BSA assay was employed to assess the proteolytic activity of lysosomes. **e**, **f** Quantification of DQ-Red-BSA fluorescent spots revealed a significant decrease of number and intensity in Rit2 KD neurons (*n* = 3). Data are represented as median, boxes show the IQ and whiskers show min–max. *p < 0.05, ***p < 0.001, unpaired two-tailed Student’s *t* test.

Enhanced Rit2 expression in pathological conditions, like G2019S-LRRK2 mutation, reduces the accumulation of pS129-aSyn in vitro and in vivo and rescues lysosomal deficits. However, the precise function of endogenous Rit2 in the ALP is unclear. Therefore, we used previously characterized SH-SY5Y Rit2 knock-out (KO) cells^60^ to investigate the physiological involvement of Rit2 in different stages of the ALP. First, we used the Cyto-ID assay to stain autophagosomes/autolysosomes and Rit2-KO cells displayed an increase in the number of these vesicles (Fig. 8a, b). We confirmed this result by Western blotting for LC3B and show that the Rit2-KO increases the number of autophagosomes, as represented by the LC3B-II/actin ratio. Furthermore, also the LC3B-II/LC3B-I ratio was increased in Rit2-KO cells (Supplementary Fig. 9a–c). Then, we investigated the number and morphology of lysosomes and Rit2-KO cells displayed an increased diameter and a drastically reduced number of lysosomes (Fig. 8c–e). In addition, when using the DQ-Red BSA assay to investigate proteolytic activity of the lysosomes, the Rit2 KO led to a major decrease in both the number and intensity of DQ-Red-BSA spots, indicating Rit2 is important for lysosomal catabolism (Fig. 8f–h). Of note, these phenotypes are similar to what we observed in G2019S-LRRK2 cells (bearing a downregulation of Rit2 expression, Fig. 1c) and indicate that the loss of Rit2 affects ALP functionality and is required for lysosome activity. To confirm that these phenotypes are caused by the loss of Rit2 in the cells, we carried out a rescue experiment, where we overexpressed Rit2 in the Rit2-KO cells. Nucleofection efficiency was similar to the other neuroblastoma cell lines, as protein (Supplementary Fig. 9d, e) and mRNA levels (Supplementary Fig. 9f) of Rit2 were correctly augmented by overexpression. Rit2 overexpression did not alter the number of autophagosomes/autolysosomes (Supplementary Fig. 9g, h). However, the overexpression of Rit2 in Rit2-KO cells increases the number of lysosomes (Supplementary Fig. 9i, j) and the intensity of the DQ-Red-BSA staining (Supplementary Fig. 9k, l), indicating Rit2-dependency of these phenotypes. Furthermore, we investigated total aSyn and LRRK2 levels in Rit2-KO cells with and without Rit2 overexpression. Total aSyn levels were significantly lower in Rit2-KO + Rit2 cells when compared to SH-SY5Y naïve cells (Supplementary Fig. 9m, n), but both aSyn and LRRK2 levels did not differ in Rit2-KO cells with or without Rit2 expression (Supplementary Fig. 9m–o). To assess the role of Rit2 in neuronal lysosomes, we used primary mouse dopaminergic neurons. Neurons were infected with either lentiviral particles expressing a scramble shRNA or a shRNA directed against Rit2 and expressing GFP in the same construct. The most efficient shRNA was tested in NIH-3T3 cells using Western blotting (Supplementary Fig. 10a) and the knock-down (KD) efficiency in primary neurons was determined via qPCR and was about 90% (Supplementary Fig. 10b). Analysis of Rit2 protein levels by Western blotting (Supplementary Fig. 10c, d) corroborated the significant downregulation caused by Rit2-shRNA treatment. The infection with the two lentiviral constructs did not alter TH levels (Supplementary Fig. 10e) or LC3B-II/LC3B-I ratio (Supplementary Fig. 10f) in the midbrain cultures. We then employed the Lysotracker Red and DQ-Red BSA assays to explore lysosome morphology and function in this model as well. Both lysosome number and morphology were altered consistently with neuroblastoma cells. DA neurons displayed a decreased number of lysosomes and an increase in their size with Rit2 KD (Fig. 9a–c). Moreover, lysosomal proteolytic activity was reduced with Rit2 KD, similar to the results in SH-SY5Y cells (Fig. 9d–f). In summary, we show that Rit2 modulates the ALP and is specifically required for correct morphology and functionality of the lysosomes, strengthening a role of Rit2 in the ALP and consistently, impacting aSyn accumulation.

## Discussion

In this study, we aimed at evaluating the neuroprotective role of a novel, underexplored target against aSyn neuropathology and elucidate its role in the ALP. We validated the potential of the small GTPase Rit2 in complementary in vitro and in vivo models of PD and revealed the cellular mechanism involved. In recent GWAS, *RIT2* has been associated with PD^28^ and neuropsychiatric disorders^61–63^. Here, we show *RIT2* mRNA levels are reduced in DA neurons from idiopathic PD patients, in G2019S-LRRK2 overexpressing cells and an in vivo PD mouse model. Altogether, expression analysis shows that *RIT2* is expressed by DA neurons and reduced in PD conditions.

The downregulation of *RIT2* mRNA levels constitutes the rationale to transiently overexpress Rit2 in G2019S-LRRK2 cells. Rit2 overexpression was effective in increasing lysosome numbers and reducing their size. Moreover, Rit2 was able to rescue the impaired proteolytic function of lysosomes. In summary, enhanced Rit2 expression provided similar rescue to what was observed with the application of a pharmacological LRRK2 kinase inhibitor.

Consistently, the overexpression of Rit2 reduced the number of pS129-aSyn inclusions in G2019S-LRRK2 cells. It has already been reported that autophagy impairment might constitute a causative factor in aSyn accumulation^64–66^ and autophagy/lysosome markers are altered in PD brain areas affected by LB pathology^10,67^. In our study, we specifically focused on macroautophagy since, in our cell model, this major form of autophagy was significantly affected. Nevertheless, we do not exclude an involvement of CMA that is also profoundly implicated in LRRK2 biology and aSyn pathology^49,64,68,69^. Notably, the impairment of (macro)autophagy leads to a compensatory activation of CMA and vice versa^70^. Our results suggest that Rit2 and LRRK2 function in a common pathway and physically interact. We found that in vitro Rit2 overexpression modulates the phosphorylation levels, and thus kinase activity, of LRRK2. Importantly, S1292 phosphorylation was reduced by Rit2 overexpression, whereas pS935-LRRK2 was significantly increased, critically differentiating Rit2-related effects from those of pharmacological LRRK2 kinase inhibition (which targets both phospho-sites). The G2019S-LRRK2 mutation is known to enhance aSyn inclusion formation after application of aSyn preformed fibrils. Indeed, it has been reported that LRRK2 kinase inhibition is beneficial against aSyn aggregation and neurodegeneration, highlighting that these effects are dependent on LRRK2 kinase activity^25^ and even more important because PD-associated mutations in LRRK2 increase its kinase activity^71^. Similar beneficial effects on aSyn inclusion formation and neuron loss were achieved by reducing total LRRK2 levels using antisense oligonucleotides against LRRK2^72^, strengthening the important role of the LRRK2 kinase in aSyn aggregation and associated to defective autophagy. In addition, we show that total LRRK2 protein levels were not reduced when overexpressing Rit2, contrasting with the effect of LRRK2 kinase inhibitors^56^. Phosphorylation of S935-LRRK2 is required for a stable binding to 14-3-3 proteins and a reduction in pS935-LRRK2 was shown to reduce this interaction. The disrupted interaction leads to mislocalization of LRRK2 into cytoplasmic pools and therefore, the S935-dependent 14-3-3 binding is thought to protect LRRK2 protein from degradation^73,74^. The exact mechanism behind LRRK2 destabilization is not yet understood, but it is believed to rely on LRRK2 kinase activity, since mice expressing a kinase-dead mutation in LRRK2 display reduced LRRK2 protein levels^75,76^. Understanding the exact regulation of LRRK2 protein degradation is a key point to predict side effects of LRRK2 kinase inhibitors, as it has been reported to produce lung and kidney pathology, similar to observations in LRRK2 KO mice^75^. LRRK2 inhibitors are envisaged to be administered not only to LRRK2-PD patients, but also to idiopathic PD patients, as indicated by the increase of pS1292-LRRK2 levels in urinary exosomes and brain tissue^57,77^. We found that Rit2 overexpression is capable of reducing pS1292-LRRK2 levels, without affecting S935-LRRK2 phosphorylation and total protein stability. These findings indicate that a potential strategy based on targeting Rit2 or its associated pathway(s) could be efficient in inhibiting LRRK2 while avoiding peripheral side effects induced by LRRK2 kinase inhibitors and related to reduction of total protein levels. Notably, targeting Rit2 could be a direct way to impact autophagy and aSyn aggregation. In addition, we show that the beneficial effects are specific to the mutated form of LRRK2 and not its WT form, further enlarging the possible therapeutic window.

Accordingly, our results indicate that Rit2 overexpression not only rescued motor impairments, but also strongly attenuated the loss of DA neurons and striatal DA terminals in mice. Moreover, total aSyn levels and pS129-aSyn pathology were reduced by Rit2 in vivo. In our cell model, total levels of aSyn were not changed, but pathological pS129-aSyn was strongly reduced. This might be due to the cellular model, with overall low endogenous levels of total aSyn protein, whereas in vivo aSyn levels were high due to viral expression. In addition, the G2019S-LRRK2 mutation increases the recruitment of endogenous aSyn into pathological inclusions and having a smaller impact on total aSyn levels^25^. In summary, our results suggest that enhanced Rit2 expression has specific beneficial effects on aSyn inclusion clearance, rather than a general effect on aSyn protein levels.

Viral overexpression of Rit2 in DA neurons, independently from aSyn overexpression, induced motor hyperactivity and this might be due to altered extracellular DA levels or release dynamics. It has been previously reported that Rit2 interacts directly with DAT, is required for DA trafficking, and is necessary for the inactivation of DAT^33^. This suggests that these modulations could be responsible for the increased locomotion in mice. In fact, the genetic deletion of DAT in rats increases extracellular DA lifetime, resulting in locomotor hyperactivity^78^. Interestingly, the conditional KO of Rit2 in mouse midbrain DA neurons decreases total DAT protein in the striatum, strengthening the possible modulation of DAT by Rit2^35^.

Lastly, the reduction of pS1292-LRRK2 levels in vitro and the recent observation that virally overexpressed aSyn in rats increases endogenous LRRK2 kinase activity^57^, led us to investigate LRRK2 kinase activity in our mouse model. Using a viral vector encoding A53T-aSyn we confirmed the increased pS1292-LRRK2 levels in DA neurons, indicating an increased kinase activity. The co-expression of Rit2 completely prevented the increase of pS1292-LRRK2 levels. As previously reported, aSyn overexpression results in an increase of endogenous LRRK2 activity, impacting the ALP and worsening aSyn pathology^57^. Even though we used a mutant version of aSyn for this set of experiments, WT-aSyn is believed to involve similar mechanisms of neurodegeneration, but with a slower effect^79^. These results suggest that the activation of WT-LRRK2 plays a role in idiopathic PD pathogenesis and consequently holds a strong pathogenic implication in most PD cases.

However, upstream modifiers of LRRK2 and related mechanisms are mostly unknown. *Rab7L1*, a PD risk factor^80^, has recently been suggested to recruit LRRK2 to the Golgi-network^81^ and stressed lysosomes^82^, and to stimulate its kinase activity^83^. Interestingly, Rab7L1 is a Rab GTPase, part of the RAS superfamily^84^ to which Rit2 also belongs^29^. Moreover, we previously proposed a novel hypothesis, in which GTPase-MAPK signaling is involved in the regulation of autophagy^36^. Our study provides evidence for a molecular mechanism leading to LRRK2 kinase inhibition, with Rit2 acting as a direct modifier of LRRK2. This could partly explain the reduced penetrance of the G2019S-LRRK2 mutation, when considering possible differential expression. In addition, we show the involvement of Rit2 in the ALP and its diverse effects on the lysosome, depending on its expression levels. Enhanced Rit2 expression rescues the G2019S-LRRK2-induced lysosomal defects, whereas the reduction of Rit2 levels in neuroblastoma cells and primary DA neurons induces defects in the ALP and specifically to lysosome biology. These defects are comparable to the ones detected in G2019S-LRRK2 cells, indicating that they might be caused by the reduced Rit2 expression. This notion is further supported by the partial rescue of ALP defects by re-expression of Rit2 in Rit2-KO cells. We cannot exclude any partial effect due to the clonal origin of Rit2-KO cells, however we present the same lysosomal phenotypes in primary DA neurons, which strengthens our results. In fact, genetic alterations in Rit2 can alter its expression levels, via length differences in short tandem repeats within the promoter^85^.

Reduced Rit2 levels, as observed in post-mortem PD brains, strengthen the role of Rit2 as a PD risk factor, possibly by modulating the ALP. Further studies are needed to elucidate the connection of ALP regulation and DAT endocytosis and trafficking.

In conclusion, we demonstrate that Rit2 acts both on autophagy-related processes and pS129-aSyn clearance. Our results suggest that inhibiting LRRK2 kinase activity through enhanced Rit2 expression is beneficial against ALP defects, aSyn pathology and could target not only G2019S-LRRK2 PD, but also idiopathic PD. Our results suggest Rit2, through modulation of LRRK2 activity, as a novel target for neuroprotection in PD and a modulator of the ALP.

## Methods

### Cell culture and transfection

SH-SY5Y neuroblastoma cell lines stably overexpressing wild-type (WT) or G2019S-LRRK2 were previously described^86,87^ and maintained as in ref. ^38^. SH-SY5Y Rit2 KO cells were maintained in DMEM GlutaMAX medium supplemented with 10% FBS and 1% Penicillin/Streptomycin (P/S). SK-N-SH neuroblastoma cell lines stably overexpressing A35T-aSyn were recently described and maintained as in ref. ^87^. HEK293T cells were grown in culture medium composed of MEM supplemented with 10% FBS, 1% MEM non-essential amino acids, 1% L-Glutamine, 1% P/S. All culture conditions were maintained strictly consistent across experimental groups and procedures.

Cells were transfected using the SF Cell Line 4D-Nucleofector^TM^ X kit (Lonza) with the 4D Nucleofector X unit. Nucleofection was carried out according to the manufacturer’s protocol. Briefly, 200,000 cells were resuspended in the nucleofection solution containing plasmid DNA (SC118279, Origene, eGFP in pcDNA3.1, 800 ng) and analyzed after 72 h. For CO-IP experiments, 2 × 10^6^ cells were resuspended in 100 μl of the nucleofection solution containing 2 μg of plasmid DNA. The plasmids used were: 2xMyc-LRRK2 in pCMV vector for the transient overexpression of both WT and G2019S-LRRK2; hRIT2 CDS in pcDNA3.1 vector. The plasmids were nucleofected alone or in combination. Cells were processed for co-immunoprecipitation 48 h post nucleofection.

### Primary dopaminergic neurons

Primary dopaminergic neurons were prepared as described in ref. ^88^. Neurons were infected with shRNA (scramble or RIT2) at DIV 7 with MOI of 30 and live imaging assays were performed at DIV 14 as described for each assay.

### Droplet digital PCR and qPCR

Total RNA was extracted using the RNeasy Plus Mini Kit (Qiagen). First strand cDNA was synthesized using the SuperScript VILO cDNA Synthesis Kit (Invitrogen). In total, 10 ng of cDNA was used for each reaction to assess *RIT2* mRNA gene expression (Hs01046671_m1, FAM), multiplexed with a housekeeping gene (RPP30, HEX, BioRad cat# 10031243). PCR was carried out as suggested by the manufacturer using ddPCR Supermix for probes (BioRad, cat# 11969064001).

PCR program:

95 °C 10 min

94 °C 30 s + 60 °C 1 min} 40 cycles

98 °C 10 min

4 °C ∞

After PCR amplification, the plate was analyzed in the QX200 Droplet Reader. Data were analyzed using QuantaSoftTM software from BioRad. Results are reported as Fractional abundance of gene of interest with respect to housekeeping gene.

qPCR samples for Rit2 levels in primary neurons were prepared as described above. qPCR was performed in the QuantStudio 5 Real-Time PCR System using the following oligos:

mTBP

FW: ACAGCCACTCTGGAGGAGAA

REV: GCCTGTTTCCGTAACCTCAA

mRit2

FW: GGAGCAGTTCCGTCAGGTATC

REV: ACTAAGCCTTGAAAAGCATCATCG

### Analysis of RIT2 mRNA levels from publicly available datasets

Geo Dataset GSE2014 composed of SN tissues isolated by laser capture microdissection from sporadic PD patients and controls was analyzed with 206984_s_at probe for RIT2. Geo Dataset GSE46798 composed of human iPSC-derived DA neurons from A53T mutant aSyn and corrected control lines was analyzed with ILMN_1796673 probe for *RIT2*. Finally, Geo Dataset GSE17542 composed of laser captured SNpc and VTA from TH-GFP mice was analyzed with 1448125_at probe for *RIT2*.

### Western blotting and CO-IP

Cells or tissue lysates for Western blotting were prepared as previously described in ref. ^38^. Primary antibodies used: anti-LRRK2 1:20,000 (Abcam, ab172378), anti-pS935-LRRK2 1:1000 (Abcam, ab172382), anti-pS1292-LRRK2 1:1000 (Abcam, ab206035), anti-LC3 1:1000 (Cell Signaling Technologies, 3868), anti-β-actin 1:6000 (Sigma-Aldrich, A-5316), anti-aSyn (Abnova, MAB5383), anti-Myc (9E10 hybridoma supernatant, 1:100), total aSyn (CST 2628), pS129-aSyn (WAKO, 015-25191). Chemiluminescence images were acquired using Chemidoc Touch (BioRad) and relative band intensity levels were calculated using ImageLab software (BioRad). For CO-IP experiments cells were mechanically lysed in lysis buffer composed of 50 mM Tris-HCl (pH = 7.6), 150 mM NaCl, 1% IGEPAL CA-630, 0.1% SDS (Sodium Dodecyl Sulfate), protease/phosphatase inhibitors cocktail (Roche, 4693124001 and 4906837001) and incubated 30 min on ice. Total lysates were cleared by centrifugation for 10 min at 13,000 × *g* at 4 °C. Protein concentration was assessed by BCA assay (Pierce, Thermo Scientific, 23225) and samples were adjusted to a concentration of 1 mg/ml. Pre-clearing was performed incubating samples with protein A/G magnetic beads (Fisher, PI88803) with gentle agitation for 30 min, 4 °C. After pre-clearing, 0.5 mg of protein extracts were incubated overnight at 4 °C with 5 μg of rabbit anti-Rit2 antibody (Origene, TA501701), or with same species IgG (Life Technologies, 10400C) as negative control, followed by incubation with 25 μl of protein A/G-magnetic beads for 2 h at 4 °C. After centrifugation protein G-agarose pellets were washed and resuspended in 2X sample buffer and processed for SDS-PAGE as in ref. ^38^.

### Lysotracker Deep Red, DQ-Red-BSA and Cyto-ID staining

To investigate lysosome morphology, we utilized the Lysotracker Deep Red dye (Molecular Probes, L12492) following the manufacturer’s instructions, as in ref. ^38^. To study the proteolytic activity of lysosomes, the fluorescent DQ-Red-BSA dye (Molecular Probes, D12051) was used following manufacturer’s instructions, as in ref. ^38^. To investigate autophagic vesicles we used the CYTO-ID® Autophagy Detection Kit in live cells, which uses a novel dye that selectively labels accumulated autophagic vacuoles. The assay was carried out following manufacturer’s instructions. Briefly, 2 μl of CYTO-ID® Green Detection Reagent were diluted in the assay buffer and then incubated for 30 min in the incubator. Imaging was carried out in complete media. In Lysotracker and Cyto-ID experiments, the number of vesicles per cell was obtained by using Cell Profiler software to assess the number of fluorescent puncta and divide it by the number of nuclei (representing the number of cells) in the same image field.

### Immunofluorescence and immunohistochemistry

Cells were fixed in 4% PFA, permeabilized, blocked and incubated with primary and secondary antibodies, as described in ref. ^38^. The primary antibodies were mouse anti-pS129-aSyn 1:2000 (Abcam, ab184674). The secondary antibodies were: Donkey anti-Mouse Secondary Antibody, Alexa Fluor 555 (A31570). Visualization was performed using a Leica SP8-X confocal laser scanning microscope system equipped with an oil immersion ×63 objective and images were analyzed using CellProfiler to quantify the number and intensity of investigated objects (object size in pixel units: pS129-aSyn 20-250; pipelines available upon request). Immunohistochemistry (IHC) was performed as described in ref. ^88^. For DA neuron survival quantification, sections of midbrain (4 sections interval) were stained with NeuN (Millipore, MAB377, 1:500) and TH (Pel-Freez Biologicals, P60101, 1:1000) antibodies, followed by incubation with Cy3 (Jackson Immuno, 715-165-150, 1:200) and Alexa Fluor 647 (Life Technologies, A-21448, 1:400) respectively. Images were acquired using a slide scanner (TissueScope^TM^, Huron Digital Pathology). Both NeuN and TH-positive neurons were counted using a stereology software with optical fractioning (Stereo Investigator, mbf bioscience). For fluorescence quantification of the striatal dopamine terminals, sections were labeled with a TH antibody (P60101) and incubated with Alexa Fluor 647 (Life Technologies, A-21448, 1:400). For aSyn neuropathology, sections were labeled for pS129-aSyn (custom antibody, 73C6 1 μg/ml, see following section) followed by Alexa Fluor 647 (Life Technologies, A-31571, 1:400). We used our custom 73C6 antibody for pS129-aSyn quantification because the signal obtained on brain sections was stronger and more specific than with ab184674 (used on cells). Images were acquired with a confocal microscope (LSM700, Carl Zeiss) and fluorescence quantification was obtained by using Corrected Total Cell Fluorescence with ImageJ software.

### 73C6 antibody production and purification

Briefly, mice were immunized with a peptide containing amino acids 111 to 135 (C-terminal) from human aSyn. B-cells were harvested and fused with myeloma cells. Hybridomas were maintained in DMEM high glucose supplemented with 10% Ultra-low IgG Fetal Bovin Serum (FBS) and Penicillin/Streptomycin (Gibco) and filtered through a 0.2 μm filter. For purification, Protein-G agarose (Thermo Fisher, cat# P120399) was used according to manufacturer’s instructions. Final IgG concentration was determined with spectrometry (Nanodrop One, Thermo Fisher). Antibody specificity was determined using Western Blotting on HEK293 cells transfected with either WT-aSyn or S129A-aSyn, with or without concomitant transfection with the PLK2 kinase (Supplementary Fig. 11).

### Animals

Heterozygous DAT-Ires-Cre mice aged between 2 and 3 months from Jackson Laboratory were used (males and females). Housing, breeding and procedures were approved by the CPAUL (Comité de protection des animaux de l’Université Laval) and the CCPA (Comité canadien de protection des animaux).

### Stereotaxic injection of AAV vectors

Mice were deeply anesthetized with isoflurane (3–4% for induction and 2% for maintenance with 0.5% oxygen). AAV-emCBA-GFP-Flex (7.5E12), AAV-CMVie-hSynP-synA53T (7.5E12) + AAV-emCBA-GFP-Flex, AAV-CAG-Flex-*Rit2*-EGFP, AAV-CAG-Flex-*Rit2*-EGFP (7E12 GC/ml) + AAV-CMVie-hSynP-synA53T were unilaterally injected in the right substantia nigra. A total volume of 1 μl was injected at 2 nl/s at the following coordinates: −3.5 mm (A/P); +1 mm (M/L); +4 mm (D/V) from bregma. Mice were euthanized 4 months after surgery.

### Behavioral tests

#### Open field

Mice were placed in the room 1 h prior to testing for habituation, and then were placed in the open field for 60 min. Open fields were used in the 2-animal monitor mode and the activity of the mice was recorded automatically with the VersaMax software. The total distance traveled (cm), horizontal activity (number of beam breaks) and vertical activity (number of beam breaks) were analyzed. The test was performed after 4 months following injection.

### Cylinder, rotation and amphetamine test

Mice were placed, 1 h prior to testing, in the room for habituation and were then placed in a cylinder with a diameter of 10 cm. Tests were performed 4 months after injection and were recorded by video. For the cylinder and rotation tests, mice were placed in the cylinder for 5 min. Forepaw use and complete rotations were analyzed manually by an investigator. For the amphetamine test, mice were injected with amphetamine at 5 mg/kg of body weight. After 15 min, they were placed in the cylinder for 30 min. Their complete rotations were counted manually by an investigator.

### Fluorescence in situ hybridization

RNAscope probes for mouse *RIT2* (RIT2—58904) were designed by Advanced Cell Diagnostics (Newark, CA, USA). The in situ hybridization was carried out following the manufacturers protocol for fixed and frozen tissue and as described in ref. ^88^.

### Proximity ligation assay (PLA)

Cells for PLA were processed as for ICC experiments. In vivo, 60 μm thick sections were used for PLA staining. Sections were washed twice in 1x PBS and then blocked for 60 min in blocking solution (1x PBS + 1% NDS + 0.2% Triton X-100) at RT. After blocking, the manufacturer’s protocol was followed. Primary antibodies (pS1292-LRRK2, Abcam ab203181; total LRRK2, UC Davis #75-36253 or ab133474, Rit2, Origene TA501704) were diluted 1:500 in Duolink II Antibody Diluent (1x) and incubated overnight at 4 °C shaking. After 2 washes of 5 min in 1x PBS + 0.1% Triton X-100, sections were incubated with PLA probes (Duolink II anti-Mouse MINUS and Duolink II anti-Rabbit PLUS) 1:5 in Antibody Diluent and incubated for 90 min at 37 °C shaking. Washes in 1x PBS + 0.1% Triton X-100 were repeated and then sections were incubated in ligation solution (1x Duolink II Ligation stock + Ligase 1:40) for 45 min at 37 °C shaking. After ligation sections were washed 2 × 5 min in 1x PBS and then incubated with an amplification solution (1x Duolink II Amplification stock + Polymerase 1:80) for 100 min at 37 °C shaking. After amplification sections were washed twice in 1x PBS for 10 min and then mounted using Fluoroshield mounting medium. Images were acquired with a confocal microscope (LSM700, Zeiss, or Leica SP8-X) and analysis of PLA spots was carried out using the “Particle Analysis” function in ImageJ or Cell Profiler (pipelines available upon request).

### Statistical analysis

Statistical analyses were performed using GraphPad Prism 8. One-way ANOVA was used in experiments comparing 3 or more groups, followed by Bonferroni’s or Sidak post hoc test for pairwise comparisons. With two experimental groups, the unpaired two-tailed Student’s *t* test or two-tailed Mann–Whitney test was utilized. Threshold for significance was set at *p* < 0.05. Data distribution was assessed using the D’Agostino-Pearson test for normal distribution. All experiments were performed in a minimum of three independent biological replicates.

## Supplementary information

Supplementary figures and legends

## Data Availability

The datasets generated and/or analyzed during the current study, and the Cell Profiler pipelines generated by for image analyses are available upon reasonable request to both the corresponding authors, through means of a data transfer agreement between the parties and with the scope of reproducing results presented in this study.
