# Supplementary material for: The small GTPase Rit2 modulates LRRK2 kinase activity, is required for lysosomal function and protects against alpha-synuclein neuropathology
Source: NPJ Parkinsons Dis. 2023 Mar 27;9:44. doi: 10.1038/s41531-023-00484-2 (PMC10042831; doi:10.1038/s41531-023-00484-2)

Supplementary Information

Supplementary Figures

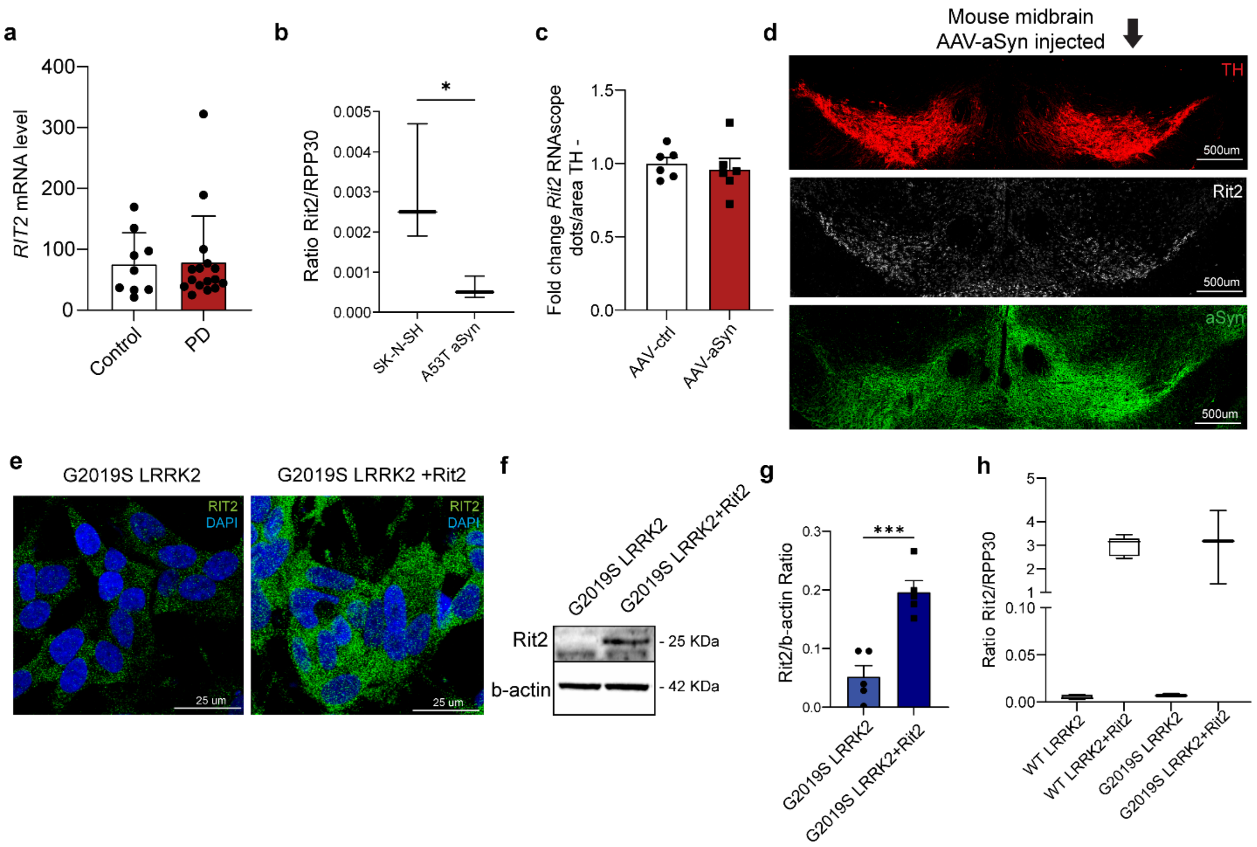

Supplementary Figure 1: *RIT2 gene expression in brain tissue and neuroblastoma cell lines.* a) *RIT2* mRNA levels are not altered in brain tissue of sporadic PD patients, when compared to controls (GSE7621, controls=9, PD=16). b) RT-qPCR was carried out to assess *RIT2* mRNA levels in recombinant neuroblastoma cell lines overexpressing A53T-aSyn. Fold change of *RIT2* mRNA is reduced in A53T-aSyn overexpressing cells (n=3). c) Rit2 RNAscope analysis in TH-areas of the midbrain are not different with aSyn viral expression. d) Low magnification overview of the AAV-aSyn injected midbrain used for the RNAscope experiment. Rit2 is visualized as mRNA, whereas TH and aSyn are stained using antibodies against the respective proteins. e) Nucleofection was used to efficiently express Rit2 in G2019S-LRRK2 cells and ICC for Rit2 was used to control for nucleofection efficiency. f) Western blot of Rit2 levels in

G2019S-LRRK2 cells with and without Rit2 nucleofection. g) Quantification of Rit2 protein levels in G2019S-LRRK2 cells. Rit2 overexpression significantly increases Rit2 levels (n=4). h) Droplet Digital PCR was carried out to assess *RIT2* mRNA levels in recombinant neuroblastoma cell lines with or without Rit2 overexpression. Ratio of *RIT2* mRNA, normalized to RPP30 is increased after Rit2 nucleofection. Data are represented as median, boxes show the IQ and whiskers show min-max or means±SEM.

*p<0.05, ***p<0.001 two-tailed Student's t-test.

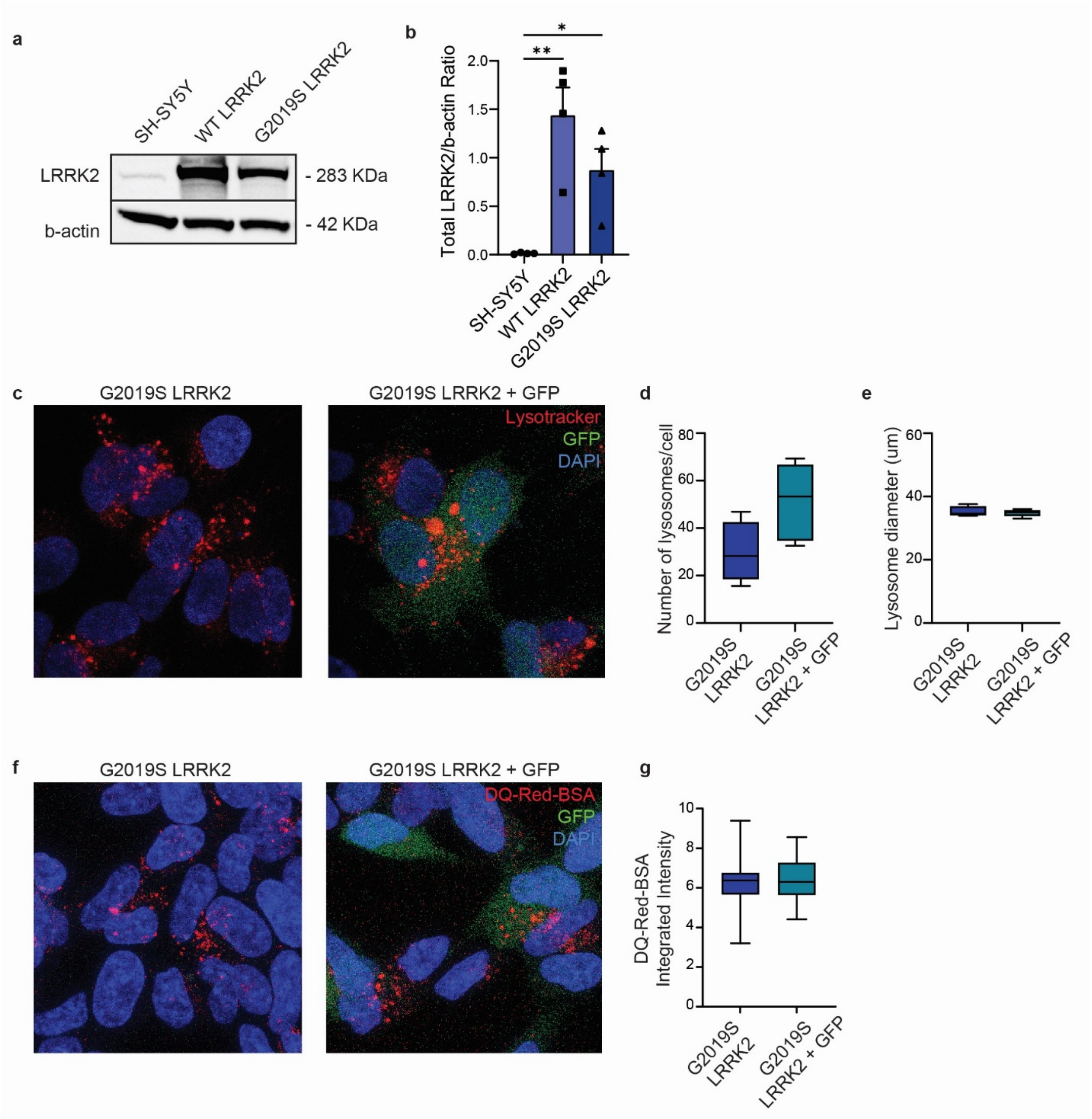

Supplementary Figure 2: *Control GFP overexpression does not modify lysosome morphology or activity.* a) Recombinant stable expression of LRRK2 in SH-SY5Y lines is confirmed by Western blotting, compared to naïve neuroblastoma SH-SY5Y cells. b) Quantification of LRRK2 protein levels in neuroblastoma SH-SY5Y cell lines stably expressing WT- or G2019S-LRRK2, compared to naïve SH-SY5Y cells (n=3). c) The Lysotracker Red dye was used to visualize cellular lysosomes in G2019S-LRRK2 cells with or without GFP nucleofection. d) The number of

lysosomes was determined and was not significantly affected by control GFP expression (n=4). e) Assessment of the diameter of lysosomes revealed that control GFP expression does not modify the size of the lysosomes in G2019S-LRRK2 cells (n=4). f) The DQ-Red-BSA assay was employed to determine the proteolytic activity of lysosomes in G2019S-LRRK2 expressing cells with and without GFP expression. g) The integrated intensity of the DQ-Red-BSA signal, that is proportional to lysosomal proteolysis, is not affected by control GFP expression (n=4).

Data are represented as median, boxes show the IQ and whiskers show min-max or means±SEM. *$p<0.05$, **$p<0.01$, one-way ANOVA followed by Bonferroni´s post-hoc test.

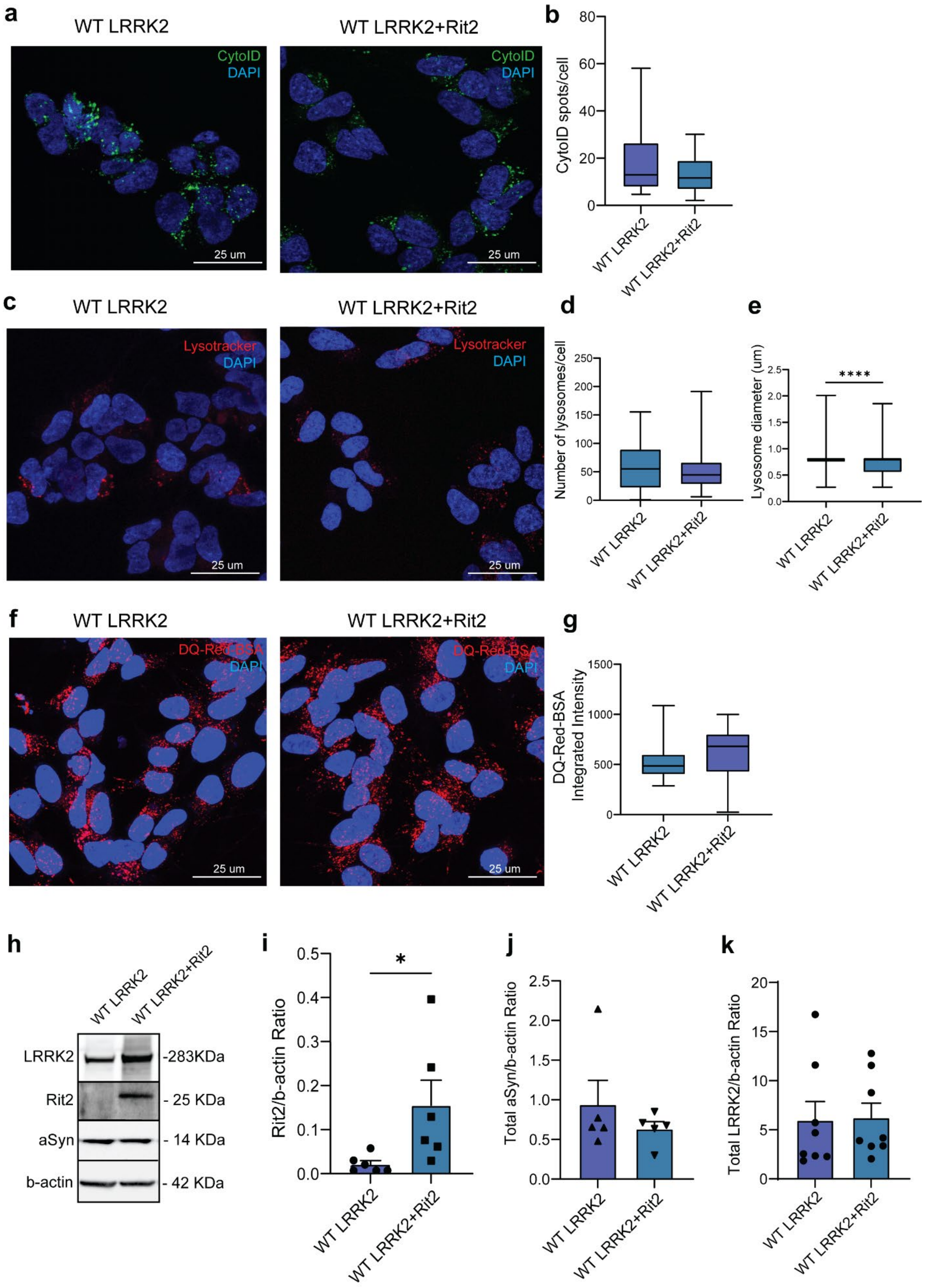

a
WT LRRK2
WT LRRK2+Rit2
CytoID
DAPI
25 um
b
CytoID spots/cell
80
60
40
20
0
WT LRRK2
WT LRRK2+Rit2
c
Lysotracker
d
Number of lysosomes/cell
250
200
150
100
50
e
Lysosome diameter (um)
2.5
2.0
1.5
1.0
0.5
0.0
****
f
DQ-Red-BSA
g
DQ-Red-BSA Integrated Intensity
1500
1000
500
h
LRRK2
Rit2
aSyn
b-actin
-283KDa
- 25 KDa
- 14 KDa
- 42 KDa
i
Rit2/b-actin Ratio
0.5
0.4
0.3
0.2
0.1
0.0
*
j
Total aSyn/b-actin Ratio
k
Total LRRK2/b-actin Ratio
20
15
10
5

Supplementary Figure 3: *Rit2 overexpression in WT-LRRK2 cells does not alter ALP phenotypes*. a) CytoID assay was employed to visualize autophagosome and autolysosome distribution. b) Quantification of CytoID-positive puncta revealed no difference in WT-LRRK2 cells, when Rit2 was overexpressed (n=4). c) Cell processing with the Lysotracker Red dye was performed to visualize lysosomes in WT-LRRK2 and WT- LRRK2+ Rit2 cells. d) The number of lysosomes per cell was quantified and revealed no difference, when Rit2 was transfected (n=4). e) The average size of lysosomes was assessed, and a significant decrease of the diameter was measured when Rit2 was transfected to G2019S-LRRK2 cells (n=4). f) The DQ-Red-BSA assay was employed to assess the proteolytic activity of lysosomes. g) Quantification of DQ-Red-BSA fluorescent spots revealed no significant difference in WT- LRRK2 cells, with Rit2 overexpression (n=4). h) Western blot analysis of total LRRK2, aSyn and Rit2 in WT-LRRK2 with or without Rit2 expression. i) Rit2 protein levels are increased after nucleofection (n=6). j) Analysis demonstrated that Rit2 overexpression does not alter total aSyn levels (n=5). k) Analysis demonstrated that Rit2 overexpression does not alter total LRRK2 levels (n=6).

In imaging experiments analysis was conducted on 700-1000 cells per group in each experiment. Data are represented as median, boxes show the IQ and whiskers show min-max or means±SEM.

*p<0.05. ****p<0.0001, two-tailed Student's t-test

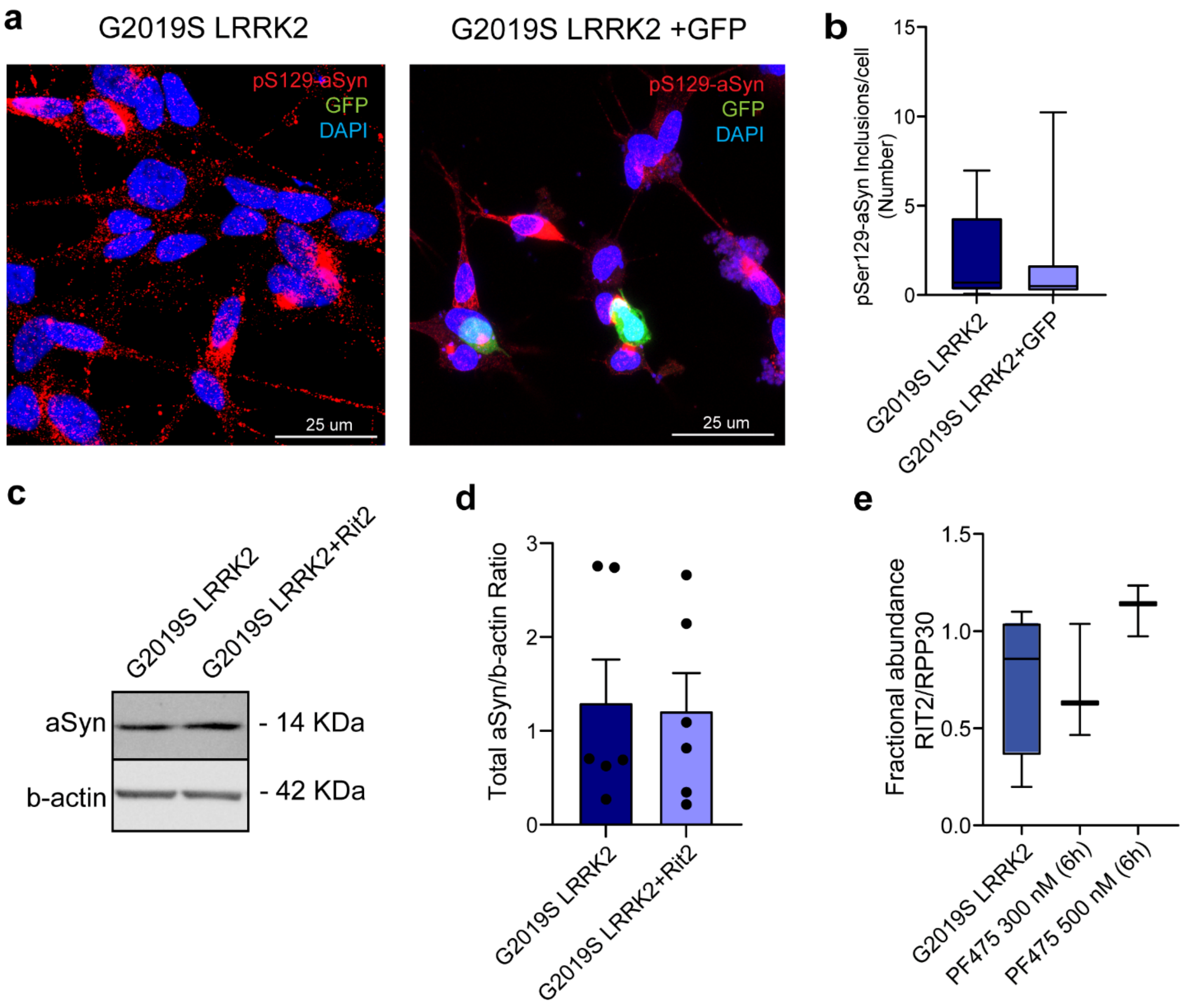

Supplementary Figure 4: *Nucleofection of Rit2 and GFP constructs in G2019S LRRK2 cells* a) Representative images for pS129-aSyn immunostaining and GFP expression in GFP-transfected G2019S-LRRK2 cells. b) Quantification of pS129-aSyn inclusions. Expression of GFP does not alter pS129-aSyn inclusion number in G2019S-LRRK2 cells. c) Western blot of total aSyn in G2019S LRRK2 cells. d) Protein levels are not changed with Rit2 overexpression in G2019S LRRK2 cells. e) *Rit2* mRNA levels were measured using ddPCR and are presented as fractional abundance of gene of interest (*RIT2*) over housekeeping gene (*RPP30*) (n=3). In imaging

experiments analysis was conducted on 700-1000 cells per group in each experiment. Data are represented as median, boxes show the IQ and whiskers show min-max or means±SEM.

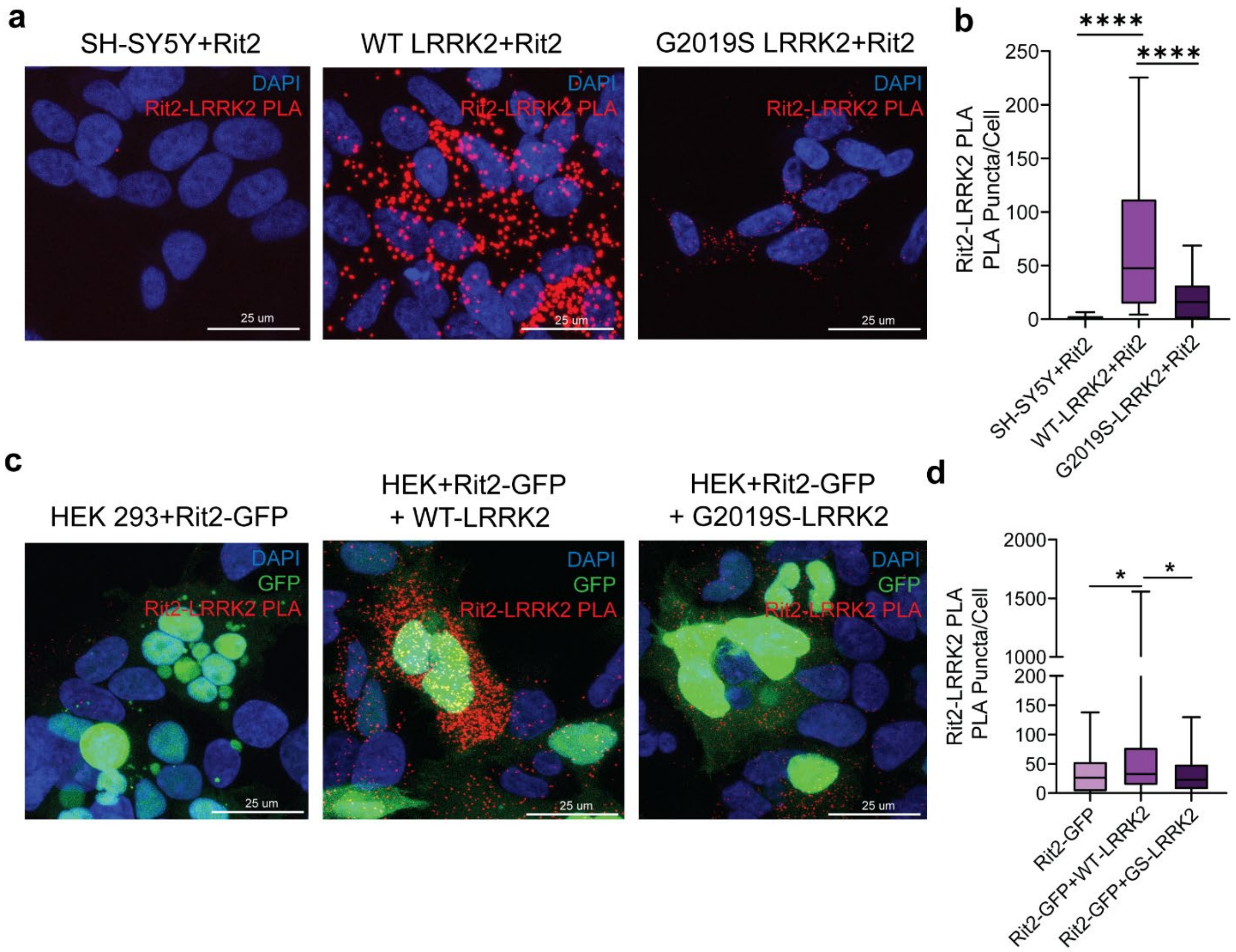

Supplementary Figure 5: *LRRK2 and Rit2 are in close proximity.* a) PLA for Rit2 and LRRK2 in neuroblastoma cells. b) Quantification of PLA puncta shows an increase of PLA signal in WT- and G2019S-LRRK2, when compared to naïve SH-SY5Y cells and a decrease when G2019S-LRRK2 cells are compared to WT LRRK2 cells. c) PLA for Rit2 and LRRK2 in HEK293 cells. d) Rit2 and (WT or mutant) LRRK2 protein were overexpressed and PLA signal quantified. The G2019S-LRRK2 mutation leads to a decreased proximity of Rit2 and LRRK2.

In imaging experiments analysis was conducted on 700-1000 cells per group in each experiment.

Data are represented as median, boxes show the IQ and whiskers show min-max.

*p<0.05, ****p<0.0001, one-way ANOVA followed by Bonferroni´s post-hoc test.

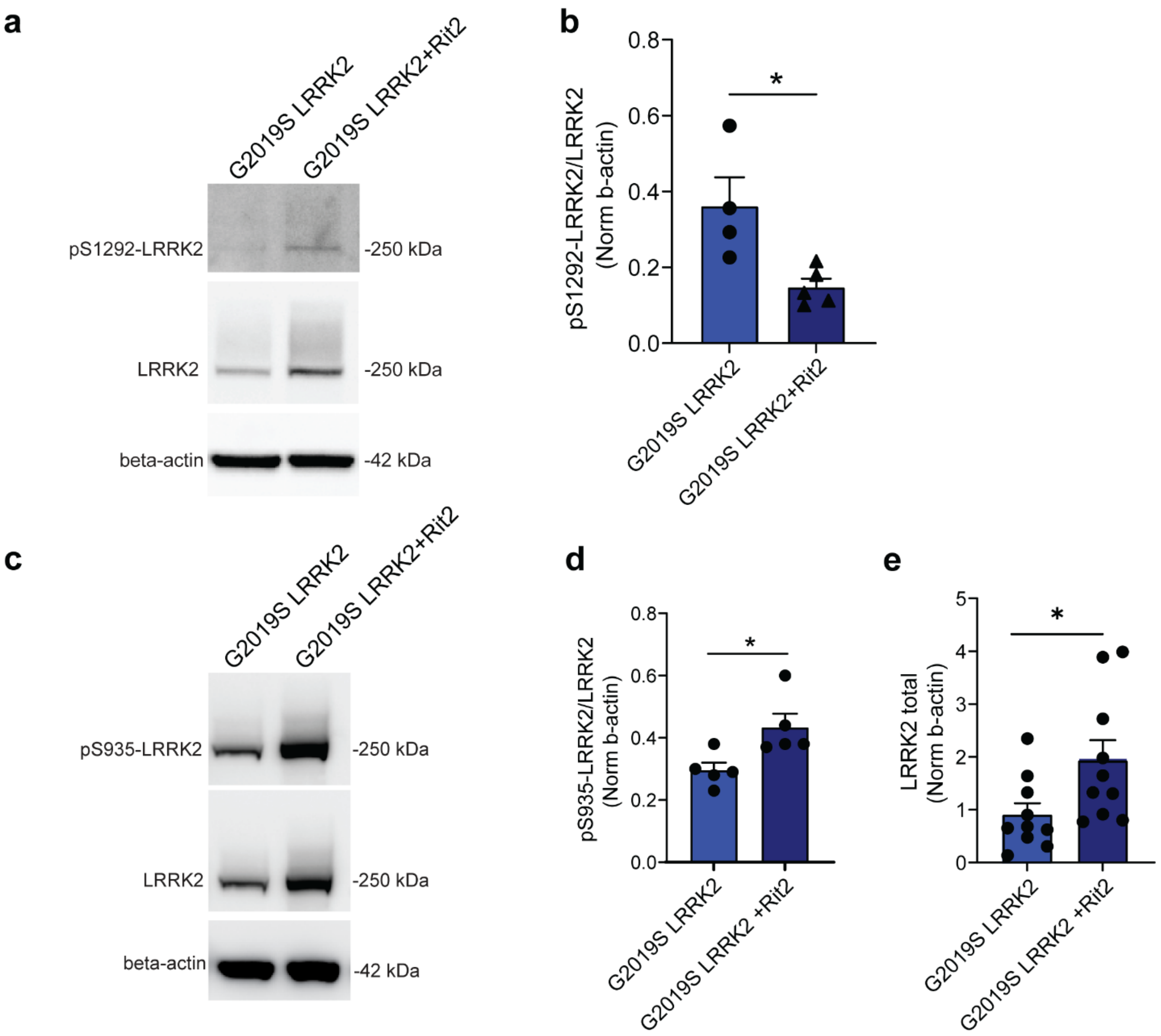

Supplementary Figure 6: *pS1292-LRRK2 levels are reduced and pS935 levels are increased with the overexpression of Rit2.*

a) Phosphorylation levels of S1292 in G2019S-LRRK2 and G2019S LRRK2+Rit2 were measured using WB for pS1292-LRRK2 and total LRRK2. b) pS1292 LRRK2 levels are reduced when Rit2 is overexpressed (normalized to total LRRK2) (n=5). c) Phosphorylation levels of S935 in G2019S-LRRK2 and G2019S LRRK2+Rit2 were measured using WB for pS935-LRRK2 and total LRRK2. d) pS935 LRRK2 levels are increased when Rit2 is overexpressed and when

normalized to total LRRK2 (n=10). e) Rit2 overexpression leads to increased total LRRK2 levels, when normalized to b-actin.

Data are means±SEM of 5-6 independent experiments for WB.

*$p<0.05$, unpaired two tailed Student´s t-test.

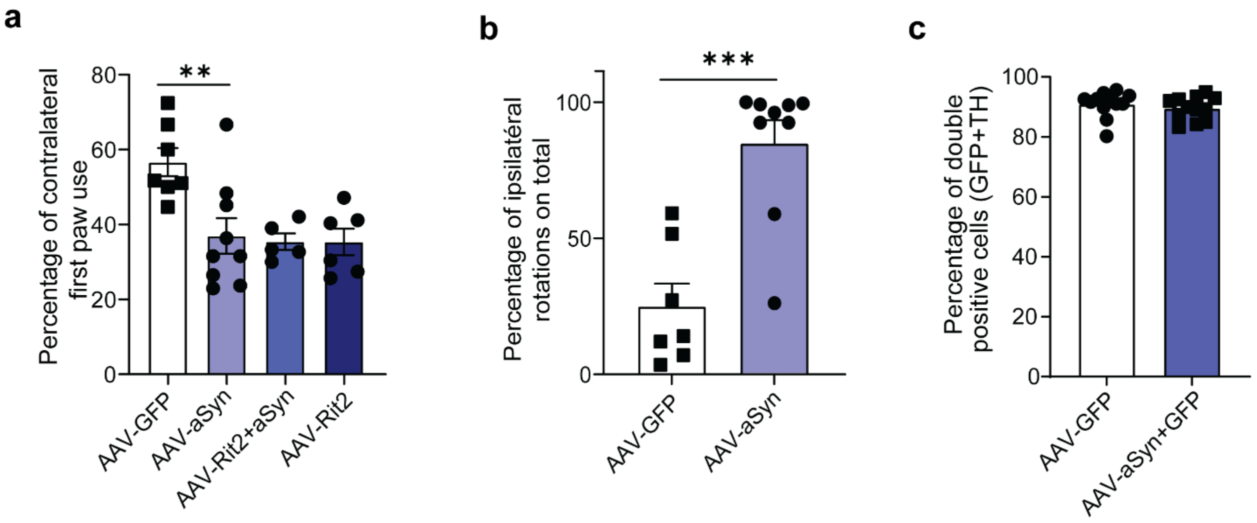

Supplementary Figure 7: *Overexpression of aSyn increases ipsilateral rotations in the amphetamine test and doesn't affect expression level in DA neurons.* a) Overexpression of A53T-aSyn reduces the percentage of contralateral forepaw use and co-injection with AAV-Flex-*Rit2* has the same effect. b) aSyn overexpression induces a significant increase in ipsilateral rotations in the cylinder with amphetamine test (n: AAV-GFP=7, AAV-aSyn=9). c) Overexpression of aSyn in the SNc does not affect TH expression. Around 90% of the cells in the ipsilateral side of AAV-GFP alone or AAV-GFP+AAV-aSyn injected mice are double positive for GFP and TH (5 animals/group). Data represented as mean ± SEM.

***p<0.001, unpaired two tailed Students's t-test. **p<0.01, one-way ANOVA followed by Bonferroni´s post-hoc test.

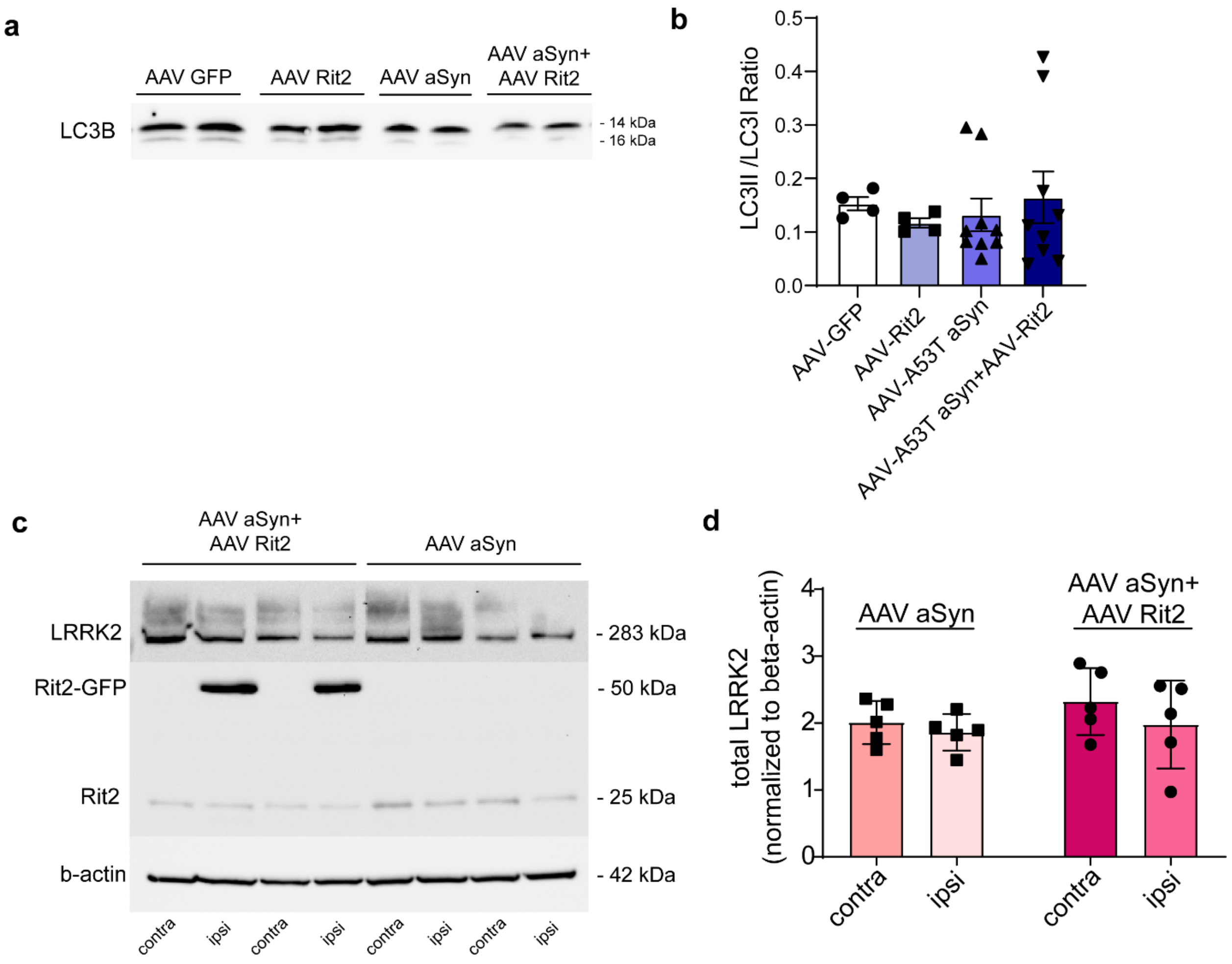

Supplementary Figure 8: *Total LRRK2 and LC3B levels are not altered by viral overexpression of aSyn and Rit2.* a) LC3BII and LC3BI levels were assessed by Western blot analysis in the different experimental groups (n=4). b) LC3BII/LC3BI ratio is not altered in the different experimental groups. c) Total LRRK2 and Rit2 levels were assessed by Western blot analysis in AAV-aSyn and AAV-aSyn+AAV- Rit2 injected mice (contra- and ipsilateral site). d) Total LRRK2 protein levels are not altered by viral overexpression of aSyn and Rit2 (normalized to b-actin) (n=5).

Data are means±SEM.

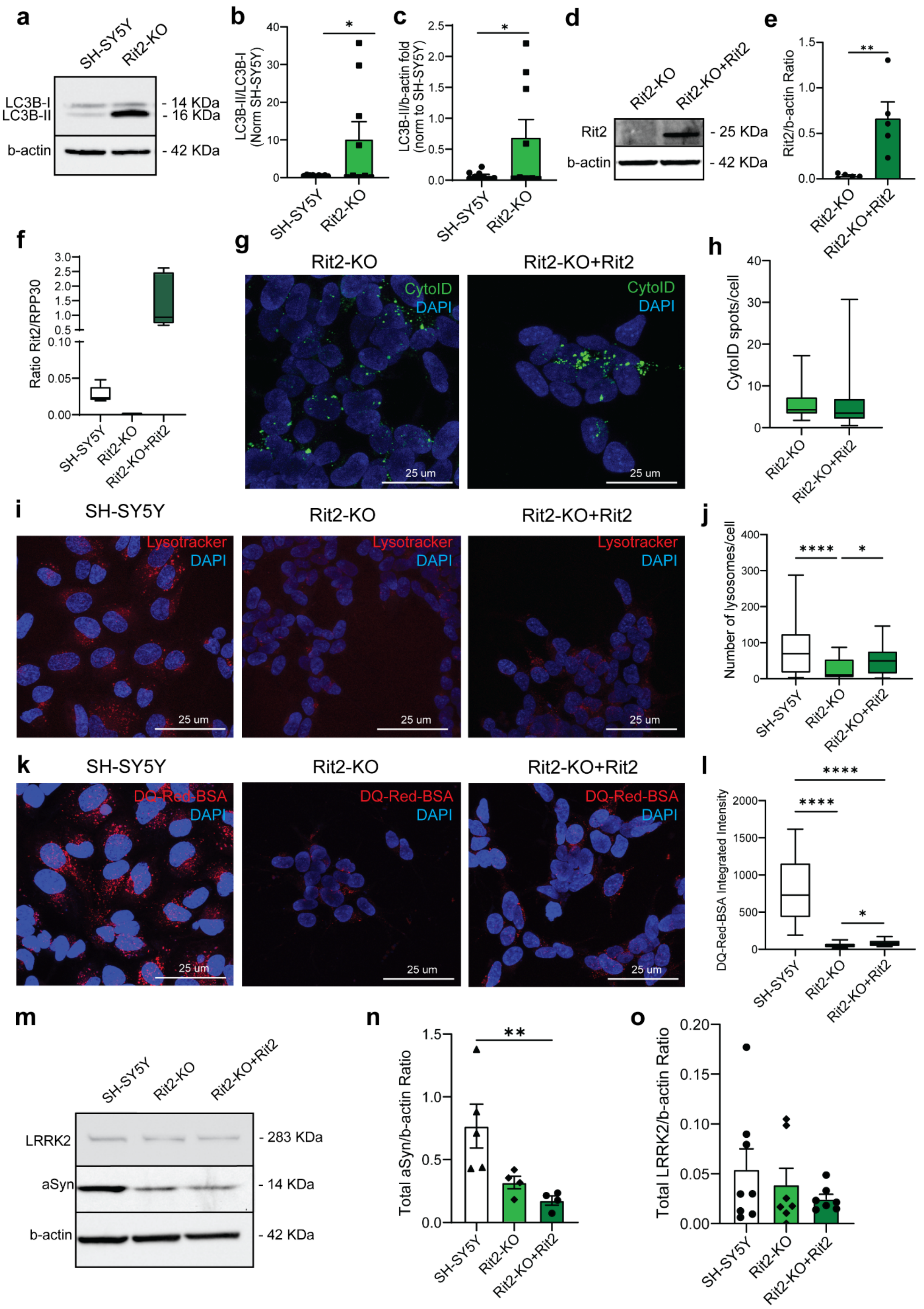

a
SH-SY5Y
Rit2-KO
LC3B-I
LC3B-II
- 14 KDa
- 16 KDa
b-actin
- 42 KDa
b
LC3B-II/LC3B-I (Norm SH-SY5Y)
c
LC3B-II/b-actin fold (norm to SH-SY5Y)
d
Rit2-KO
Rit2-KO+Rit2
Rit2
- 25 KDa
b-actin
- 42 KDa
e
Rit2/b-actin Ratio
f
Ratio Rit2/RPP30
g
CytoID
DAPI
25 um
h
CytoID spots/cell
i
Lysotracker
DAPI
j
Number of lysosomes/cell
k
DQ-Red-BSA
l
DQ-Red-BSA Integrated Intensity
m
LRRK2
- 283 KDa
aSyn
- 14 KDa
n
Total aSyn/b-actin Ratio
o
Total LRRK2/b-actin Ratio

Supplementary Figure 9: *Rit2 rescues lysosome number and functionality in Rit2-KO cells*. a) LC3BII and LC3BI levels were assessed by Western blot analysis in the different experimental groups. b) LC3BII/LC3BI ratio is increased in Rit2-KO cells. c) LC3BII/b-actin ratio is increase in Rit2-KO cells. d) Western blot of Rit2 protein levels after Rit2 nucleofection. e) Rit2 protein levels are significantly increased after nucleofection. f) Droplet Digital PCR was carried out to assess *RIT2* mRNA levels in Rit2-KO cells with or without Rit2 overexpression. Ratio of *RIT2* mRNA, normalized to RPP30 is increased after Rit2 nucleofection (n=3). g) CytoID assay was employed to visualize autophagosome and autolysosome distribution. h) Quantification of CytoID-positive puncta revealed no difference in Rit2-KO cells, when Rit2 was overexpressed (n=4). i) Cell processing with the Lysotracker Red dye was performed to visualize lysosomes in SH-SY5Y, Rit2-KO and Rit2-KO+ Rit2 cells. j) The number of lysosomes per cell was quantified and revealed a rescue of the number of lysosomes when Rit2 was transfected into Rit2-KO cells (n=4). k) The DQ-Red-BSA assay was employed to assess the proteolytic activity of lysosomes. l) Quantification of DQ-Red-BSA fluorescent spots an increase of the DQ-Red-BSA intensity in Rit2-KO cells, with Rit2 overexpression (n=4). m) Western blot analysis of total LRRK2 and aSyn in Rit2-KO cells with or without Rit2 expression. n) Analysis demonstrated that Rit2 overexpression does not alter total aSyn levels in Rit2-KO cells, but total aSyn levels are lower than in SH-SY5Y cells. (n=5). o) Analysis demonstrated that Rit2 overexpression does not alter total LRRK2 levels in Rit2-KO cells (n=6).

In imaging experiments analysis was conducted on 700-1000 cells per group in each experiment. Data are represented as median, boxes show the IQ and whiskers show min-max or means±SEM. *p<0.05, unpaired two tailed Students's t-test *p<0.05, **p<0.01 ****p<0.0001, one-way ANOVA followed by Bonferroni´s post-hoc test.

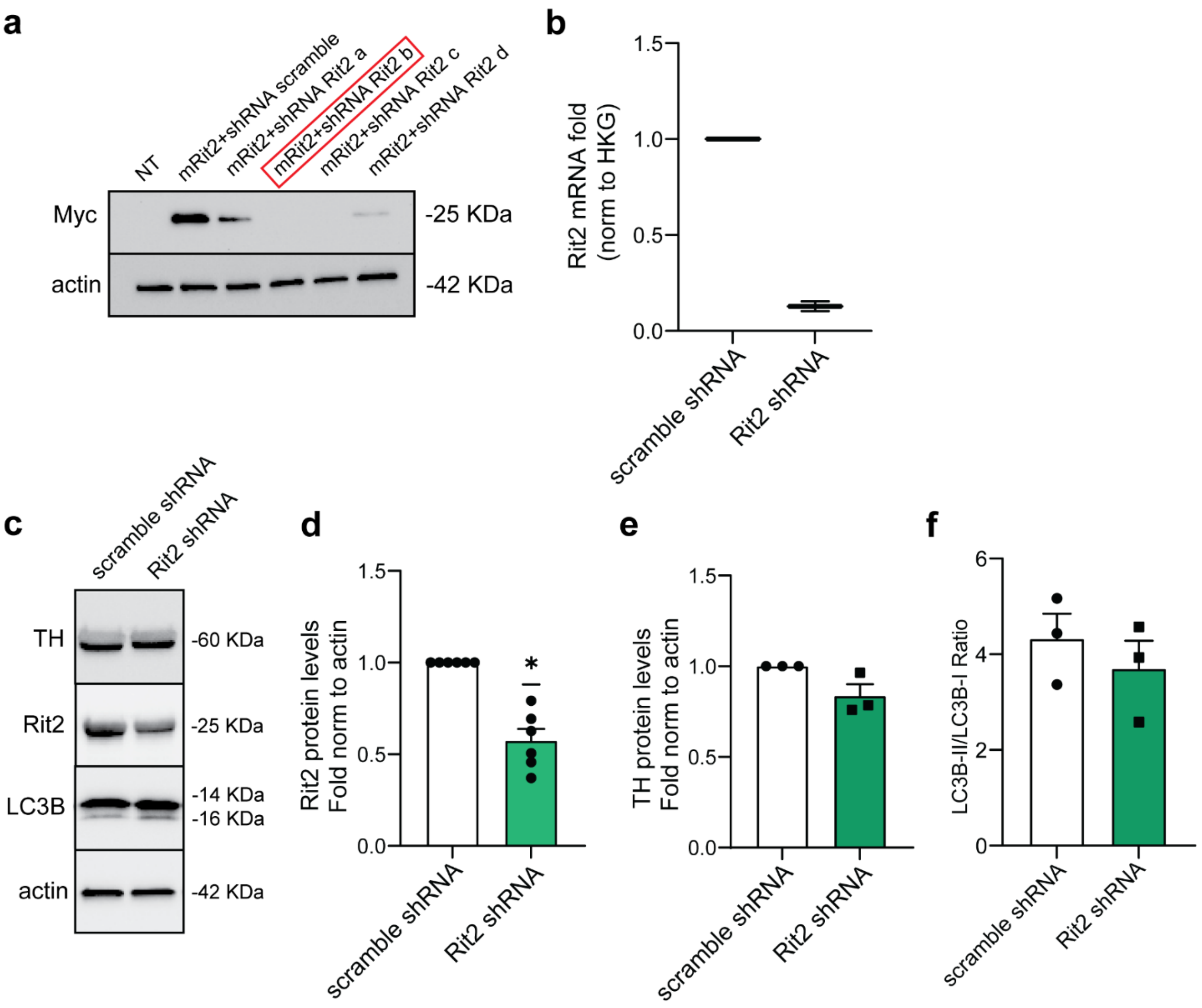

Supplementary Figure 10: *Rit2 knock-down in midbrain cultures does not alter TH or LC3B levels*. a) Four different shRNA constructs were tested in NIH-3T3 cells with the overexpression of Rit2. shRNA Rit2 b was chosen for further experiments. b) *Rit2* mRNA levels in primary dopaminergic neurons were reduced of about 90% after 7 days of shRNA expression as measured in qPCR (n=2). c) Western blot analysis of scramble and Rit2 shRNA infected midbrain cultures. d) Rit2 protein levels are decreased when midbrain cultures are infected with Rit2 shRNA (n=6). e) TH protein levels are not altered with scramble or Rit2 shRNA infection (n=3). f) LC3BII/LC3BI ratio is not altered with scramble or Rit2 shRNA infection (n=3). Data are

represented as median, boxes show the IQ and whiskers show min-max or means±SEM. One sample Wilcoxon test, *p<0.05.

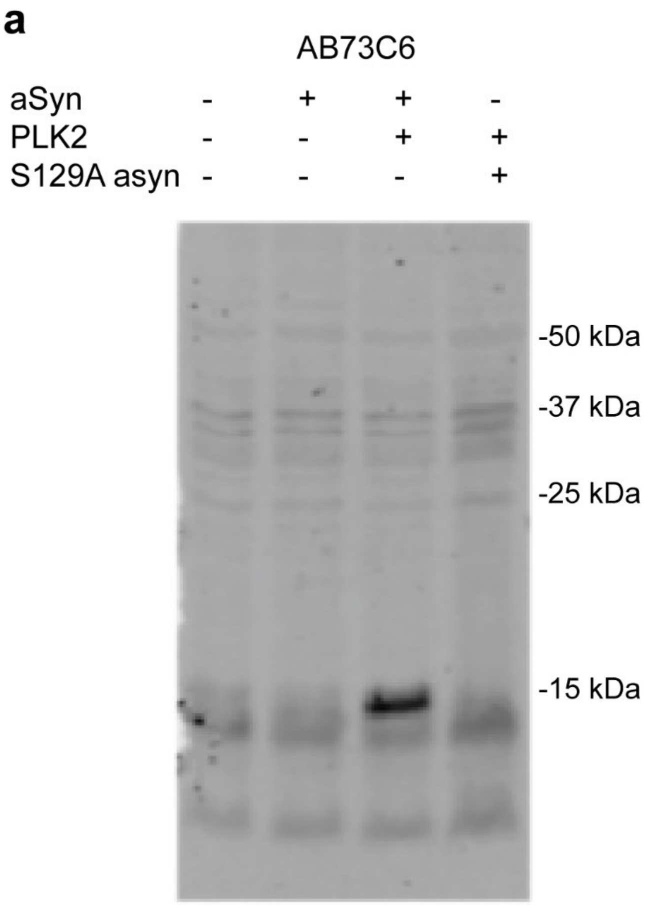

Supplementary Figure 11: *Custom antibody 73C6 is specific for pS129-aSyn.* a) HEK293 cells transfected with aSyn in combination with the PLK2 kinase, which is known to phosphorylate aSyn, results in a strong band at 15 kDa (lane 3). Cells transfected with PLK2 and a mutated version of aSyn that cannot be phosphorylated (S129A), no band is observed at 15 kDa (lane 4), indicating that the antibody recognizes specifically the phosphorylated form of aSyn. No detectable signal is observed in naïve HEK293 cells or when transfected with aSyn alone (lanes 1 and 2).

WB for LC3B (Fig 2 A-C)

N1

LC3B

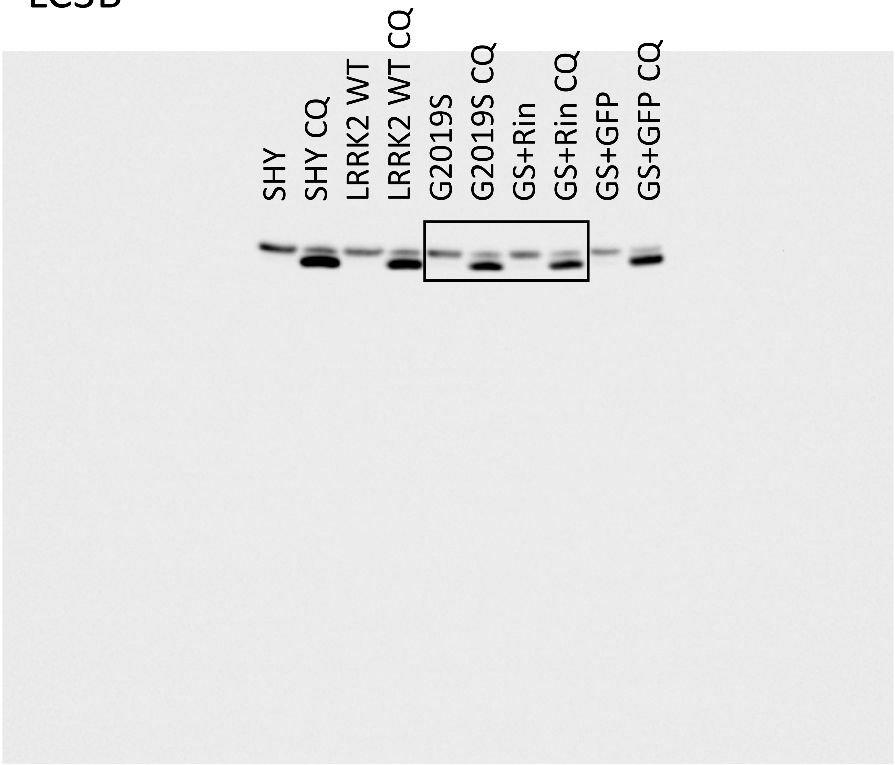

beta-actin

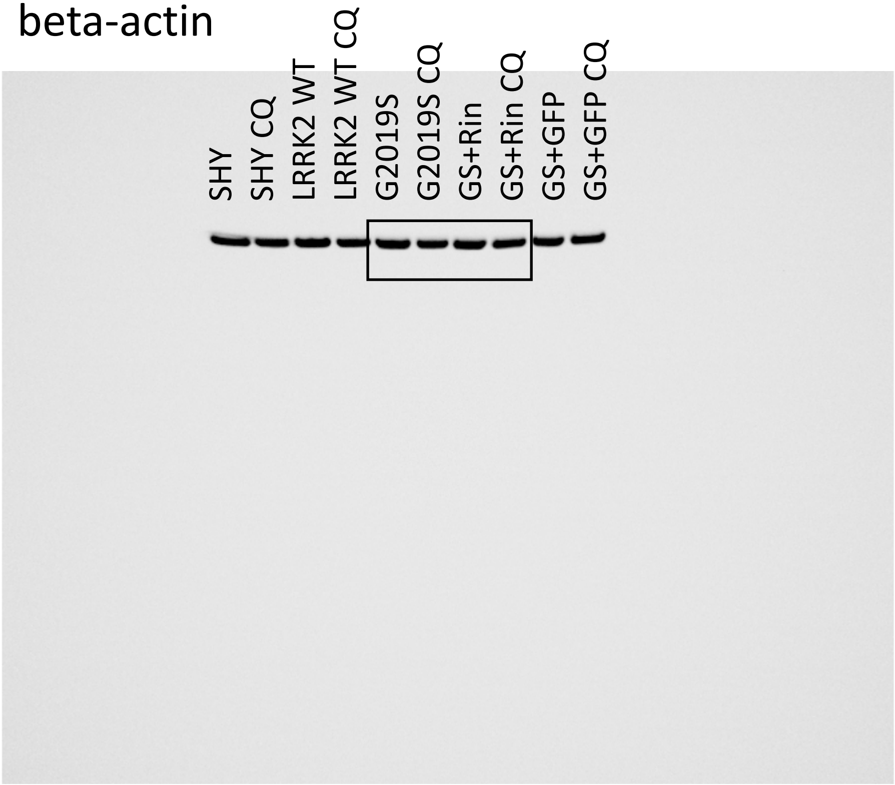

WB for LC3B (Fig 2 A-C)

N2

LC3B

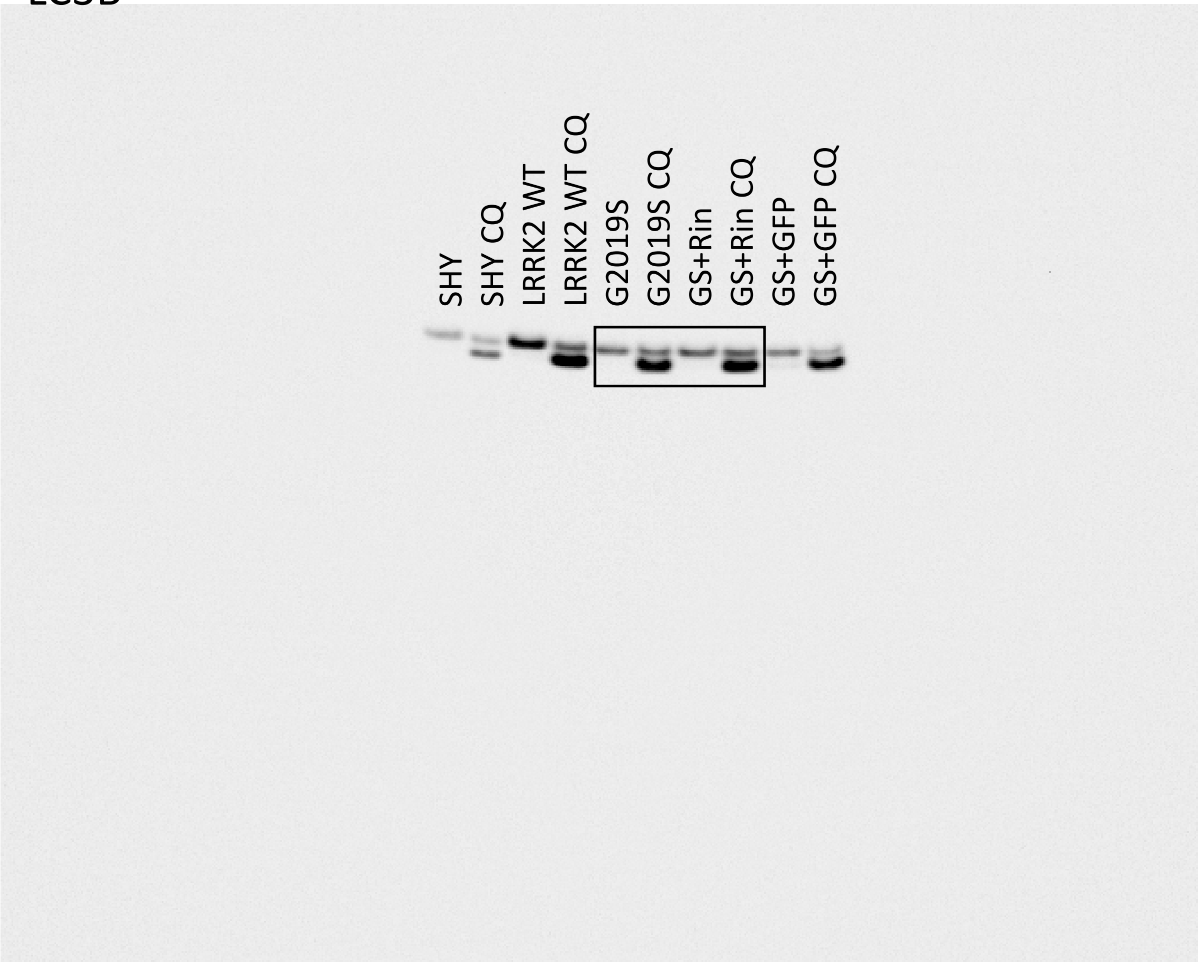

beta-actin

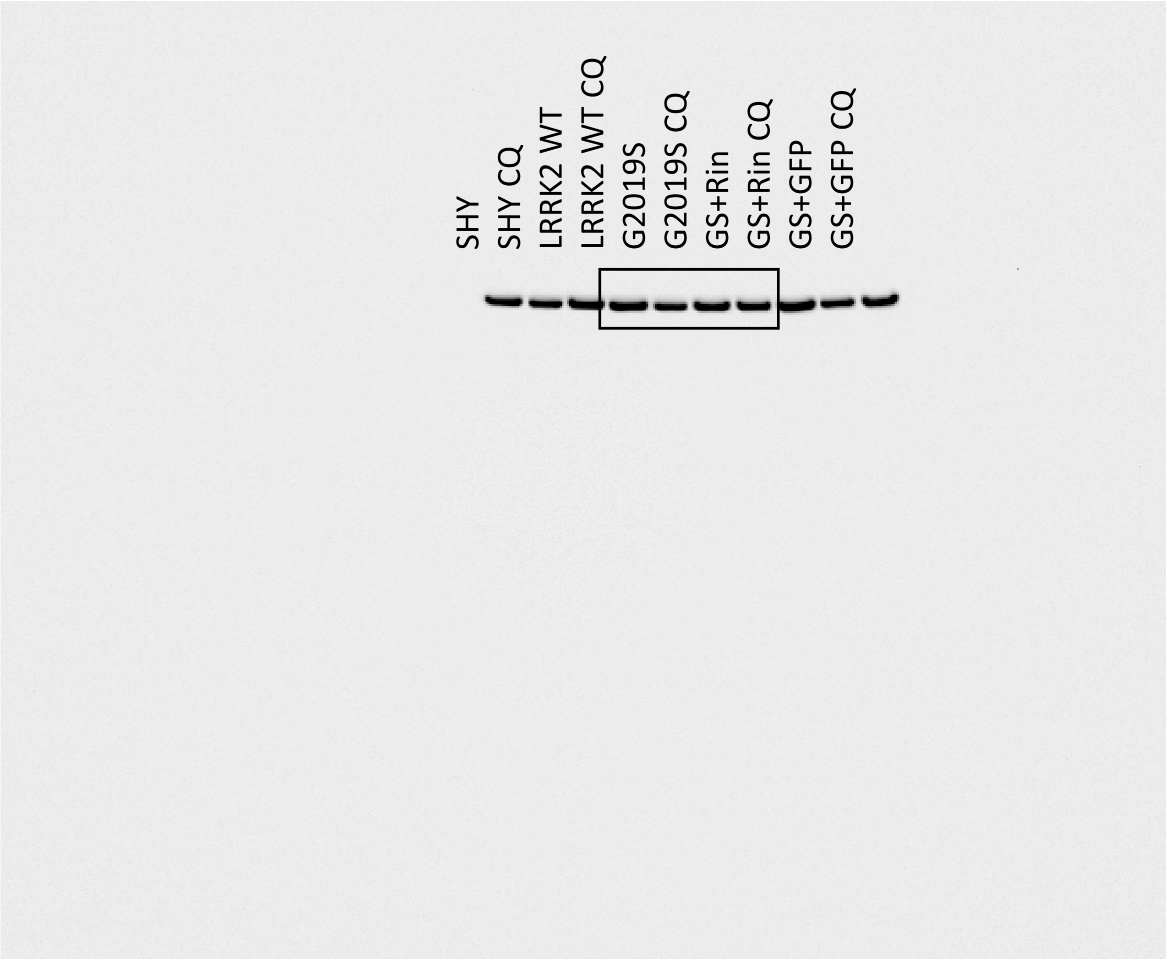

WB for LC3B (Fig 2 A-C)

N3

LC3B

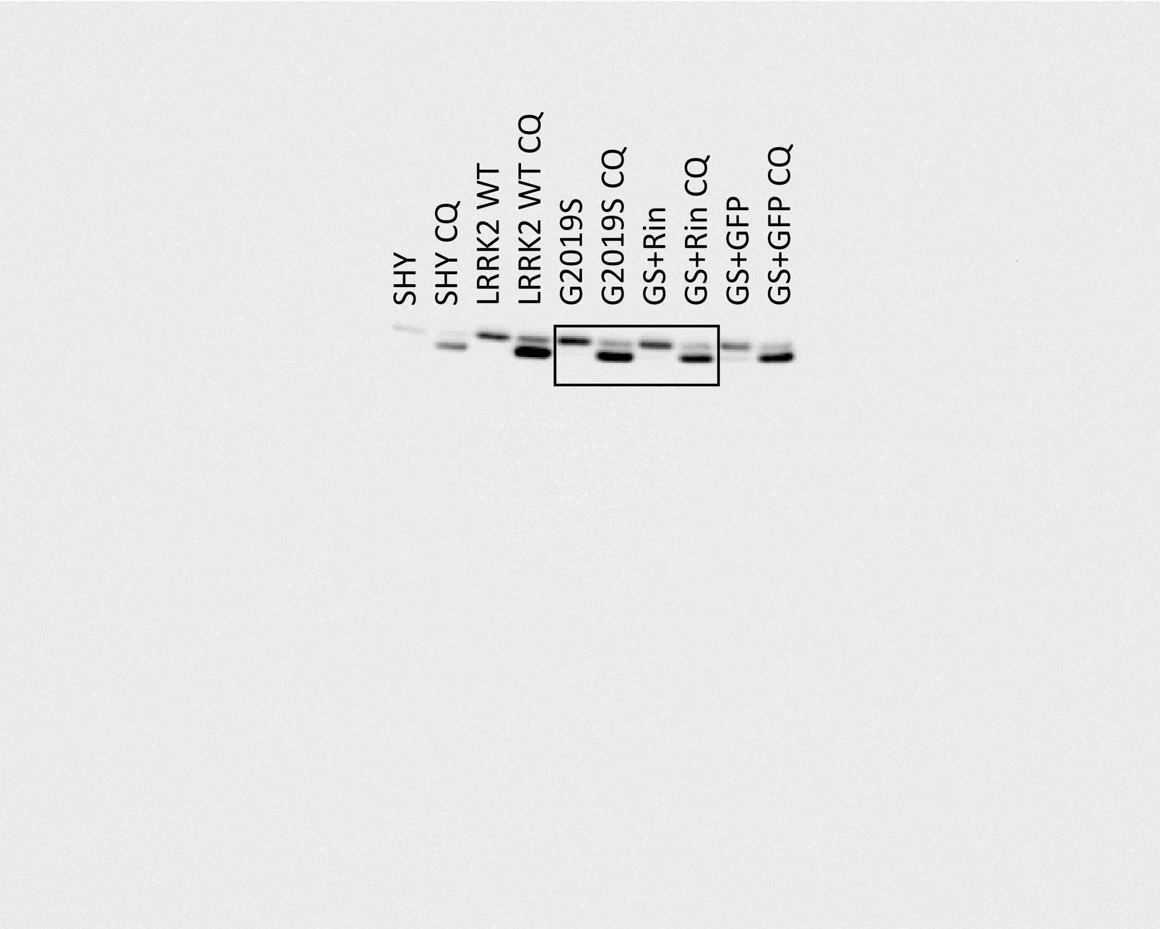

beta-actin

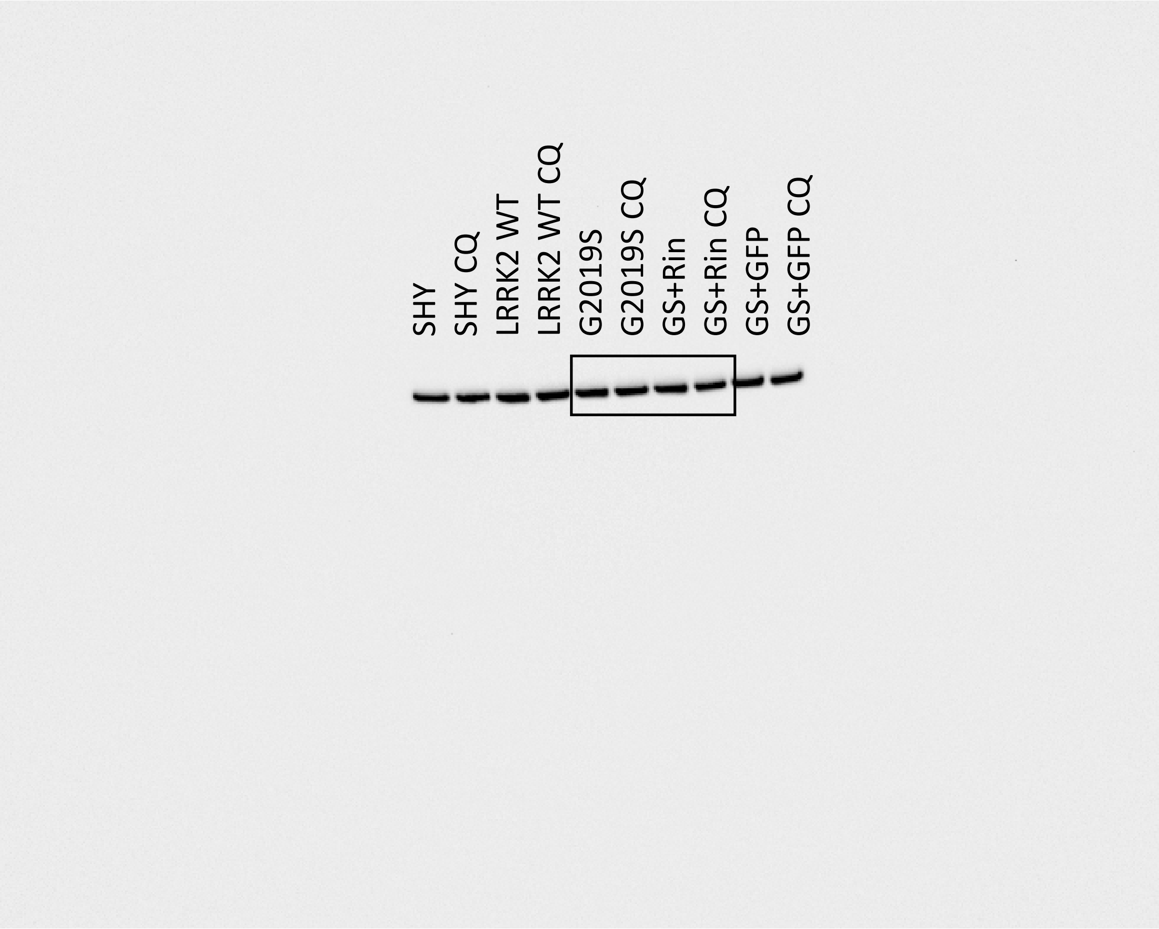

CO IP (Fig 4)

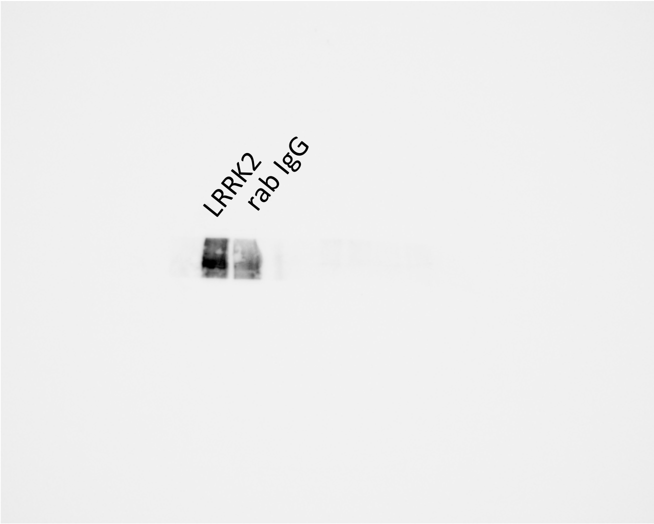

LRRK2 CO IP

LRRK2

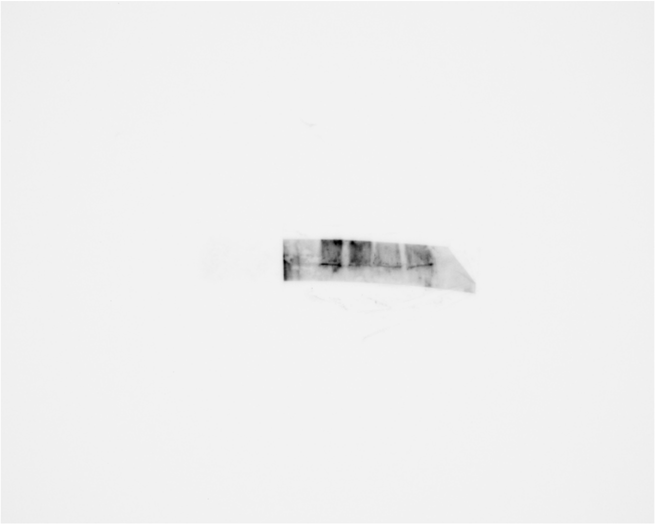

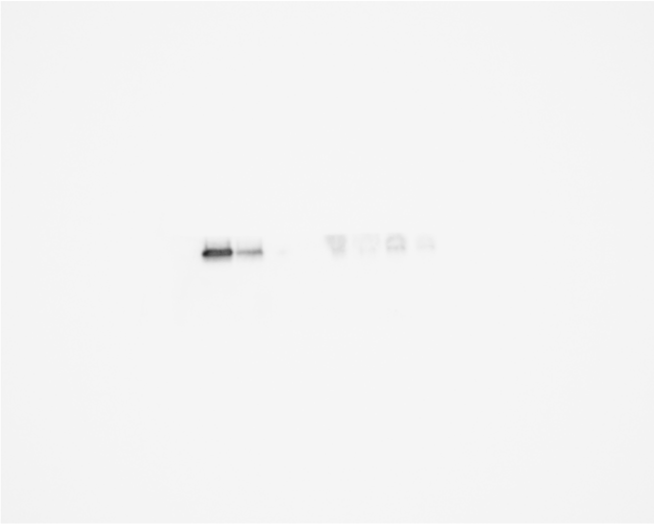

Rit2 CO IP

Rit2

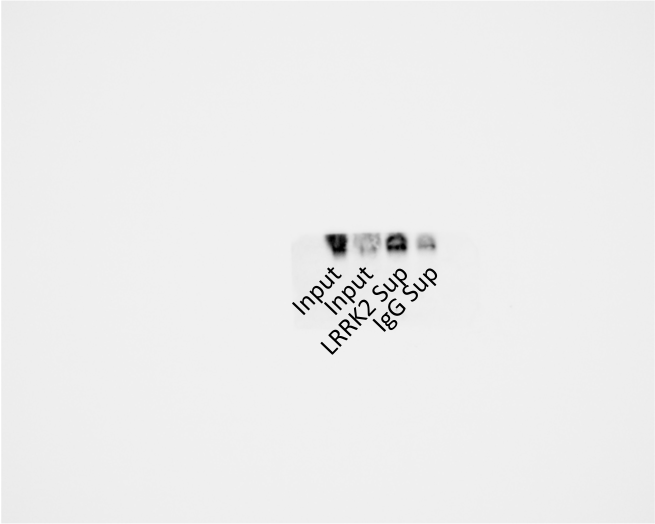

CO IP (Fig 4)

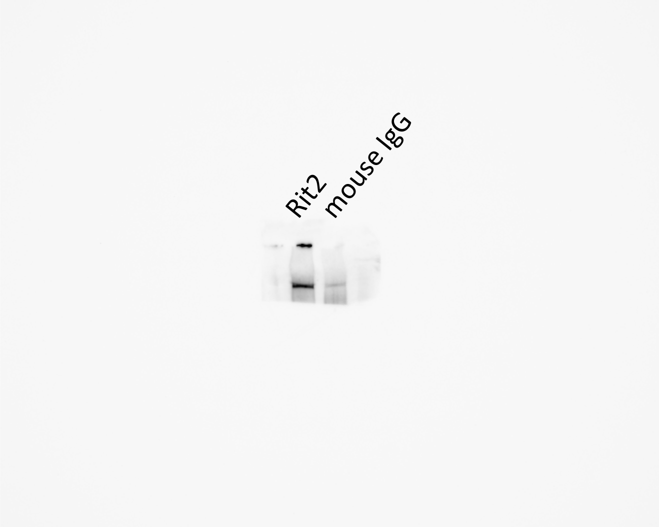

LRRK2 CO IP

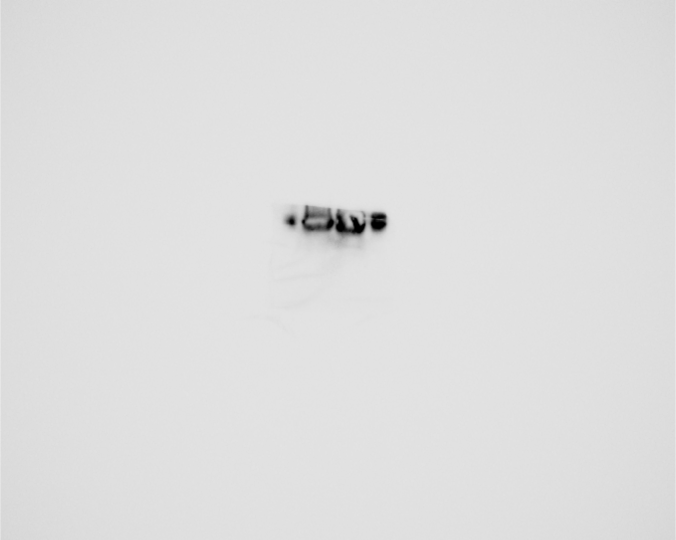

Rit2 CO IP

LRRK2

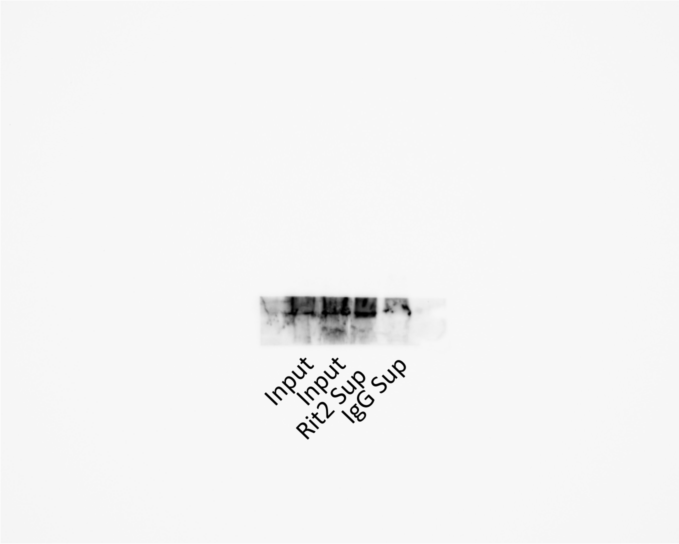

Rit2

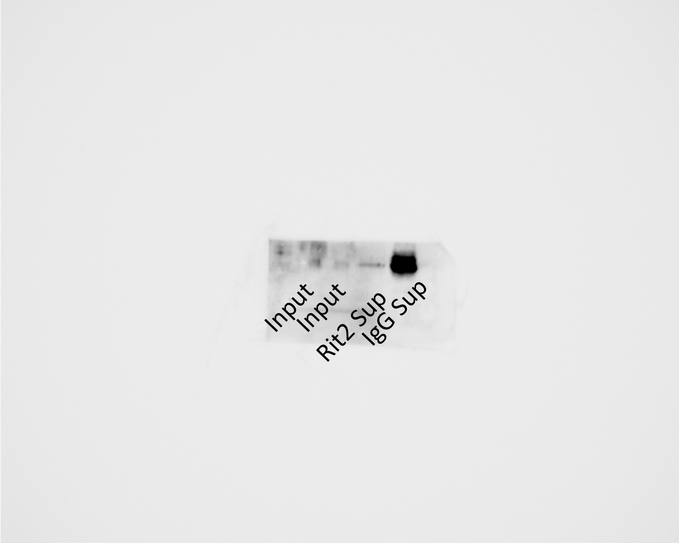

CO IP (Fig 4)

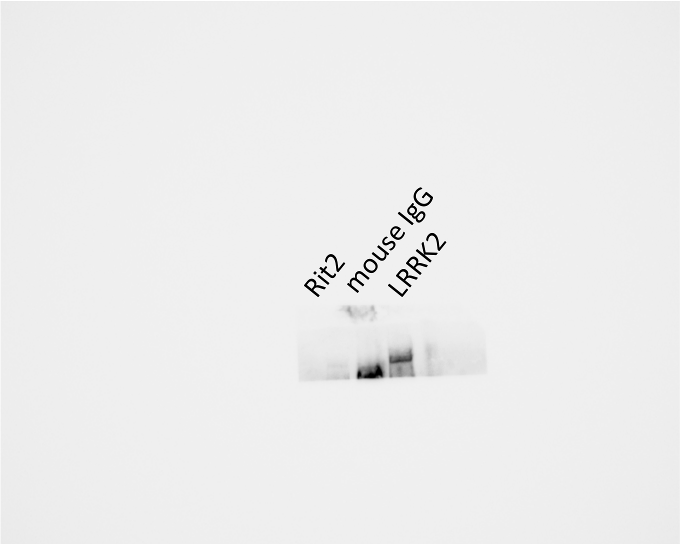

LRRK2 CO IP

LRRK2

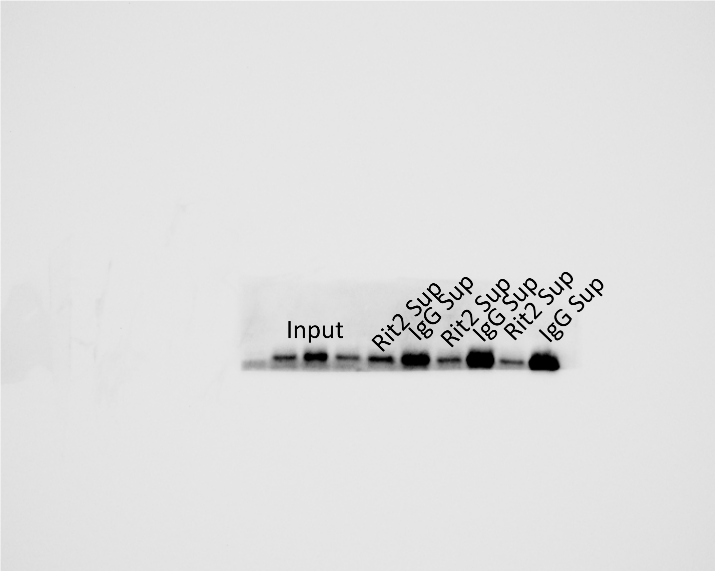

Rit2

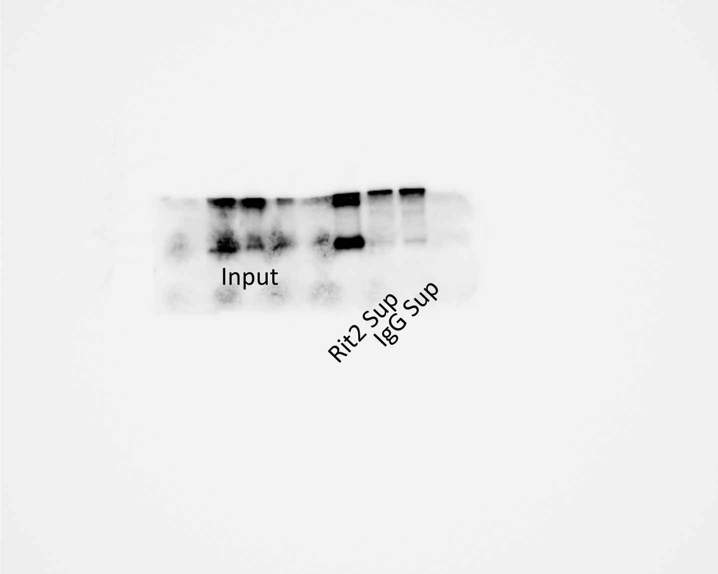

Rit2 CO IP

CO IP Fig 4

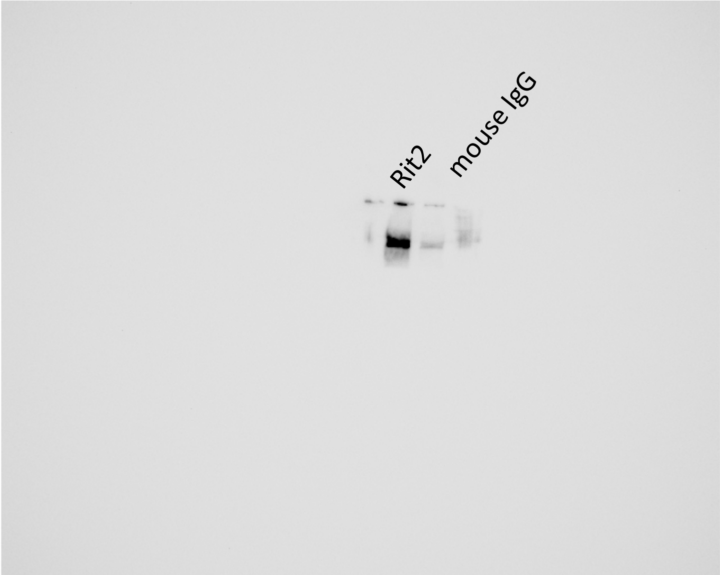

LRRK2 CO IP

LRRK2

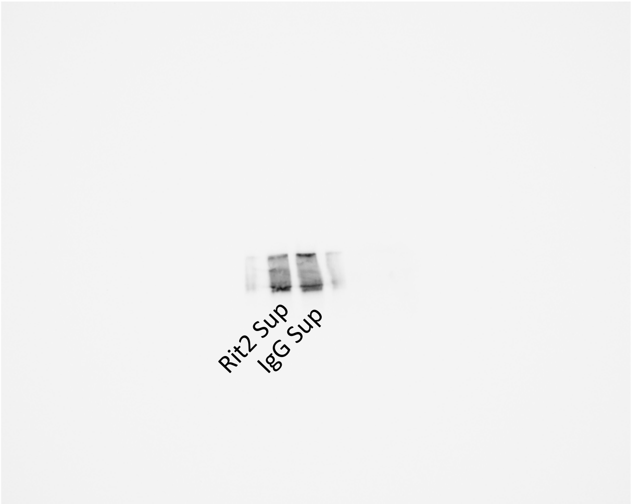

Rit2

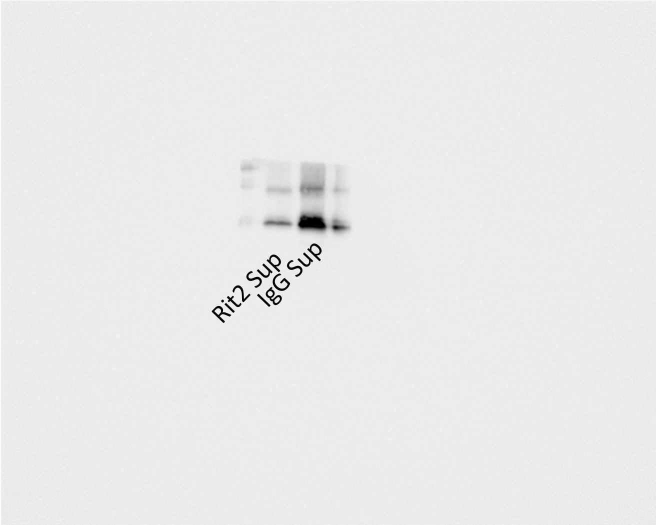

Rit2 CO IP

CO IP Fig 4

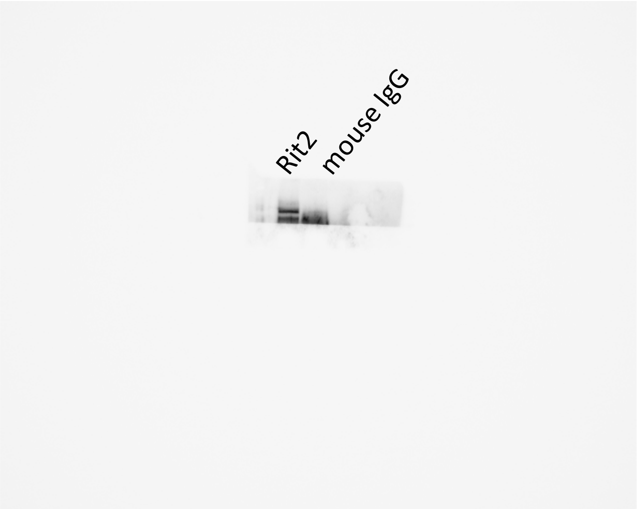

LRRK2 CO IP

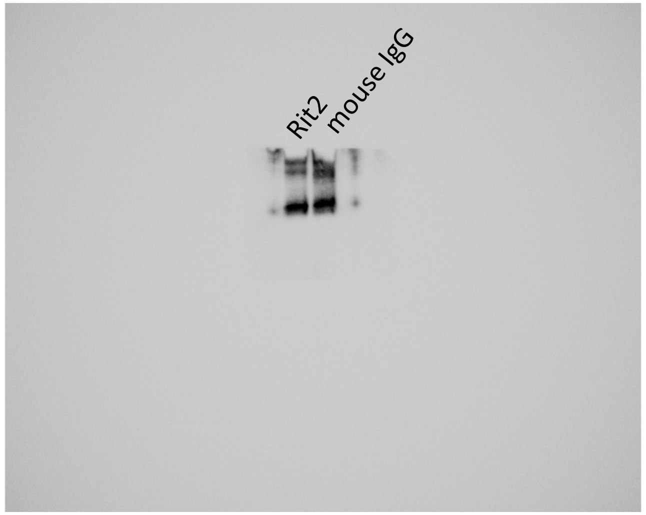

Rit2 CO IP

LRRK2

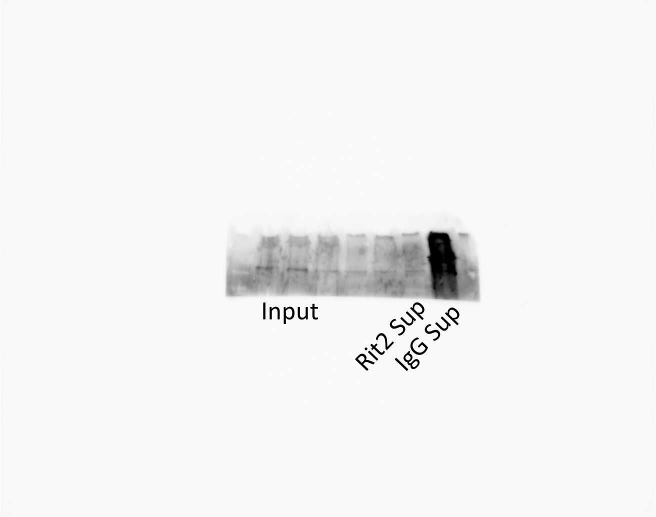

Rit2

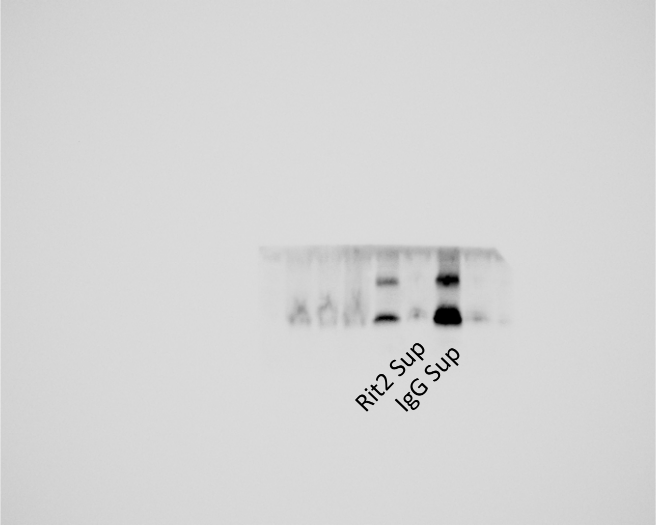

WB for total aSyn (Fig S4)

N1

aSyn

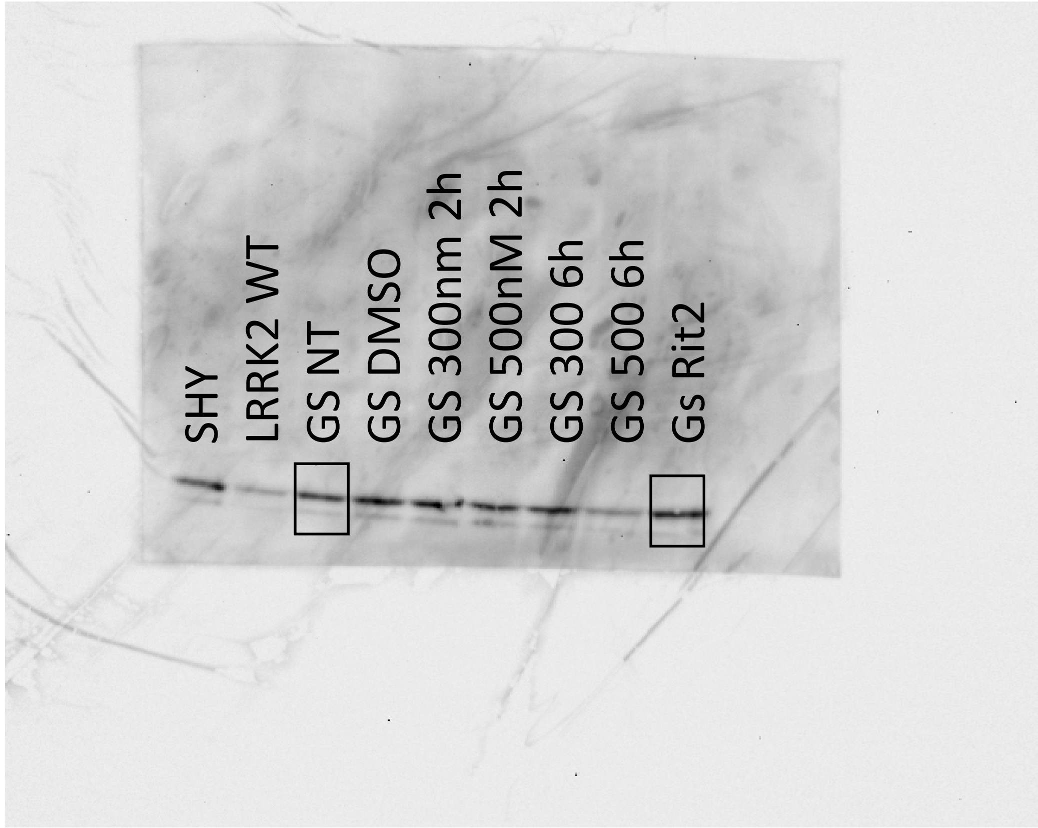

actin

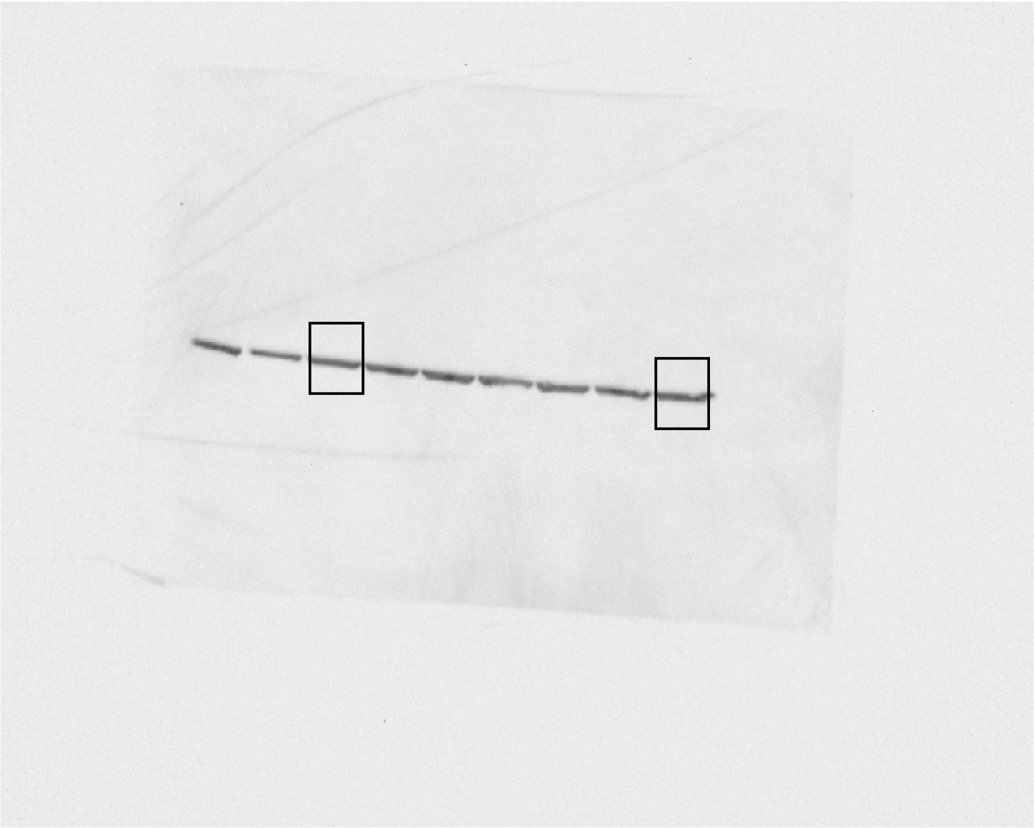

WB for total aSyn (Fig S4)

N2

aSyn

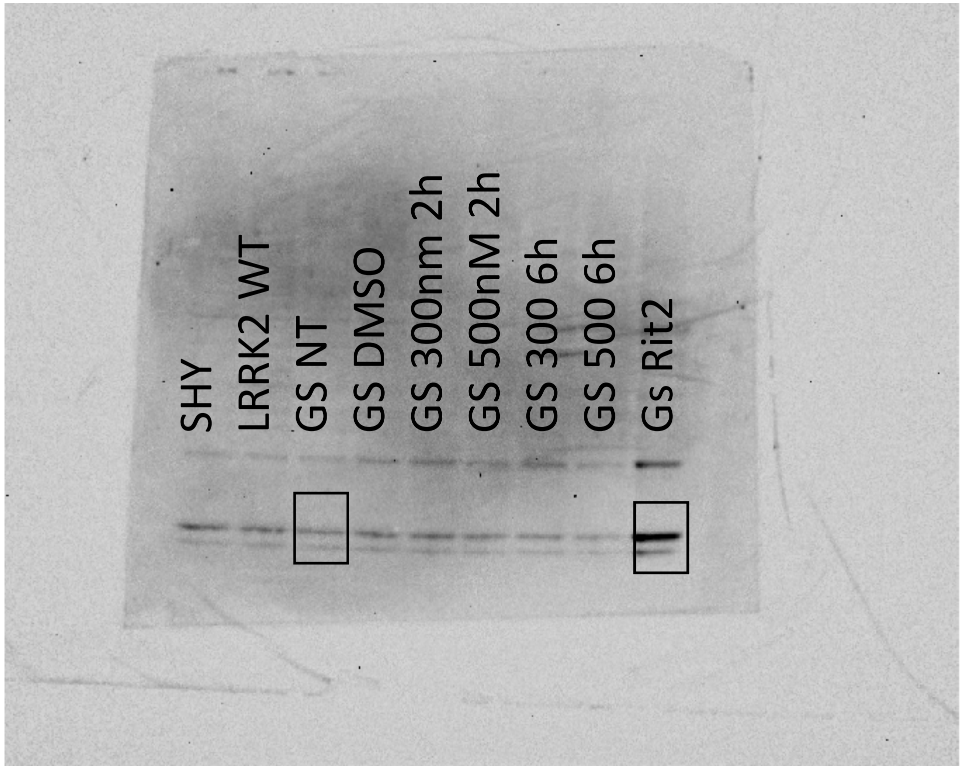

actin

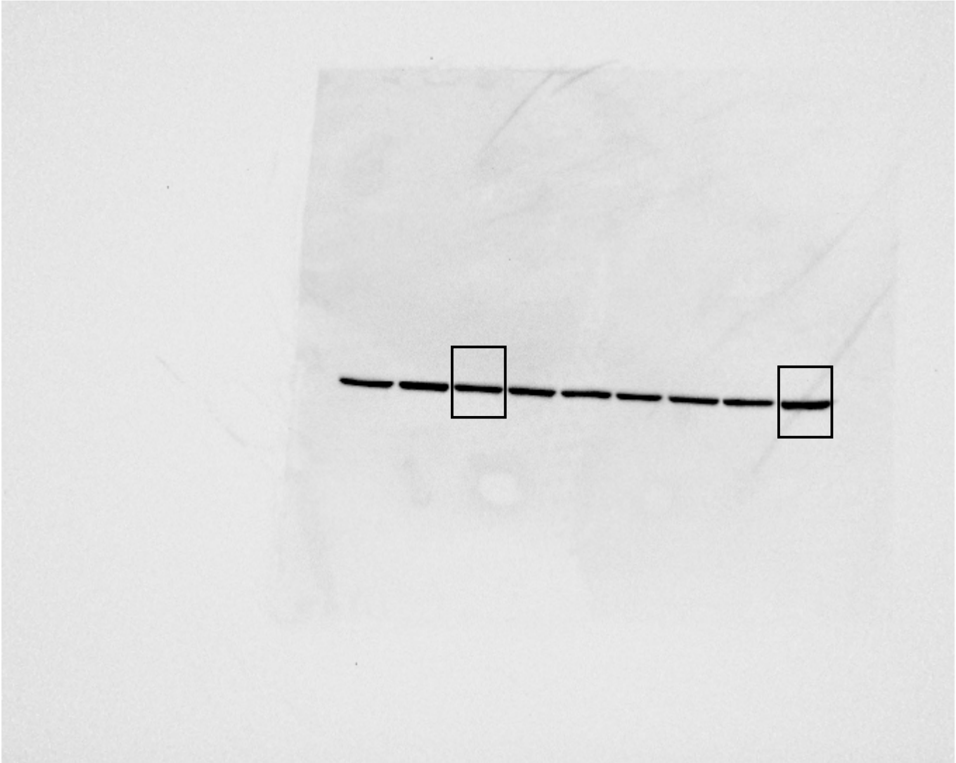

WB for total aSyn (Fig S4)

N3

aSyn

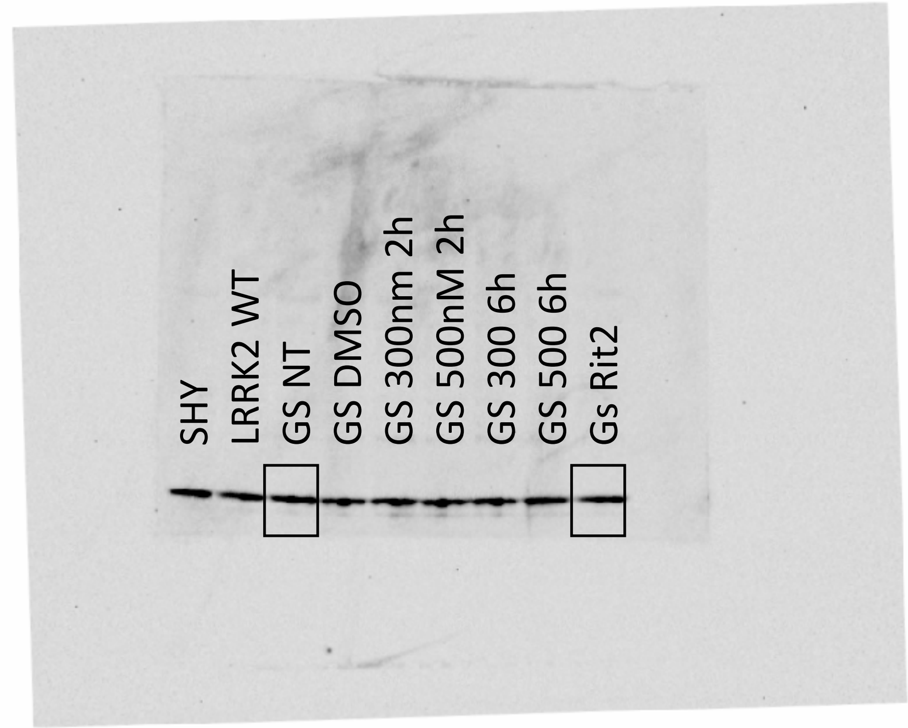

actin

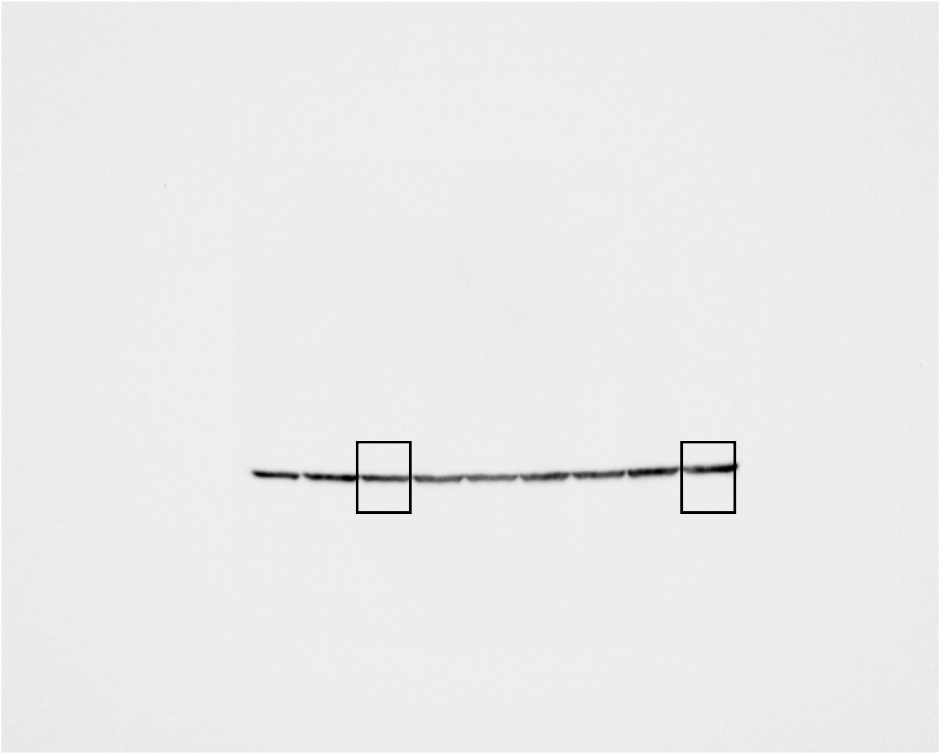

WB for total aSyn (Fig S4)

N4

aSyn

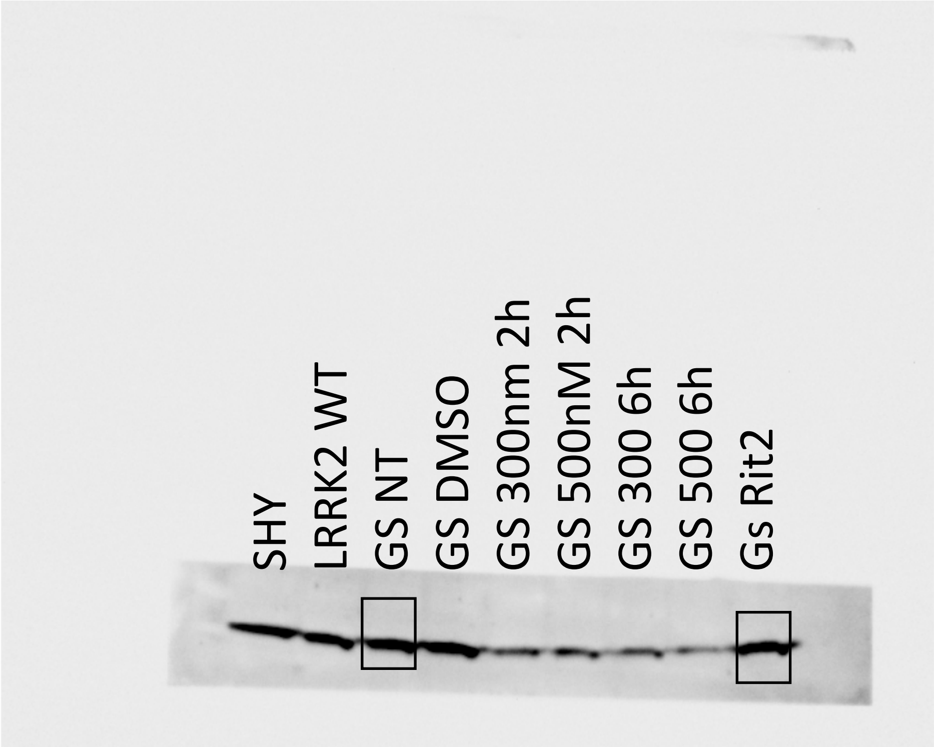

actin

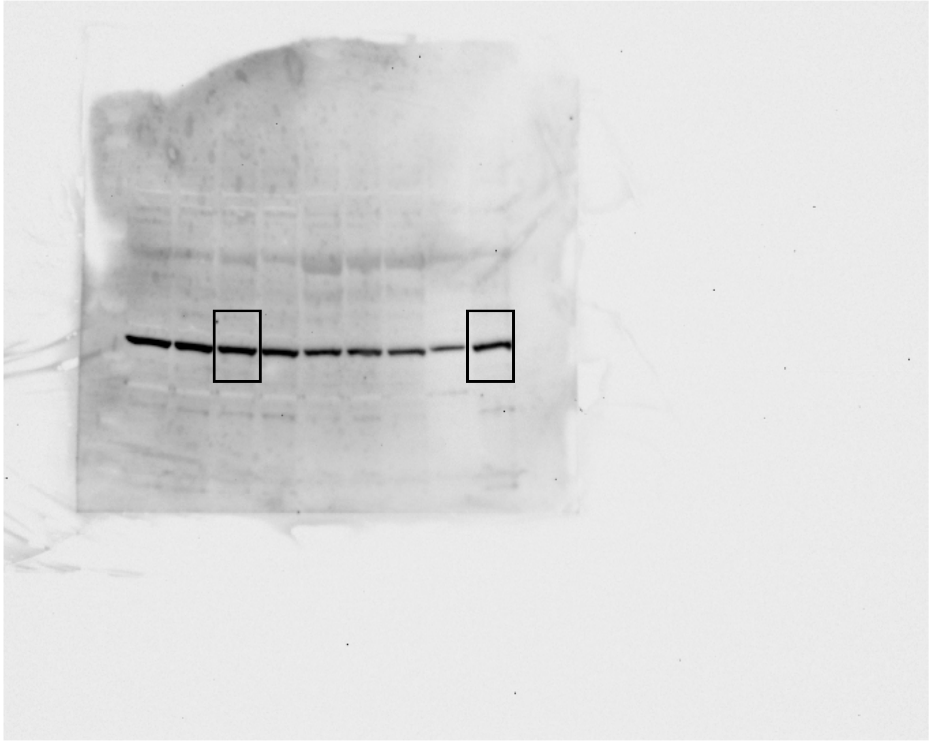

WB for total aSyn (Fig S4)

N5

aSyn

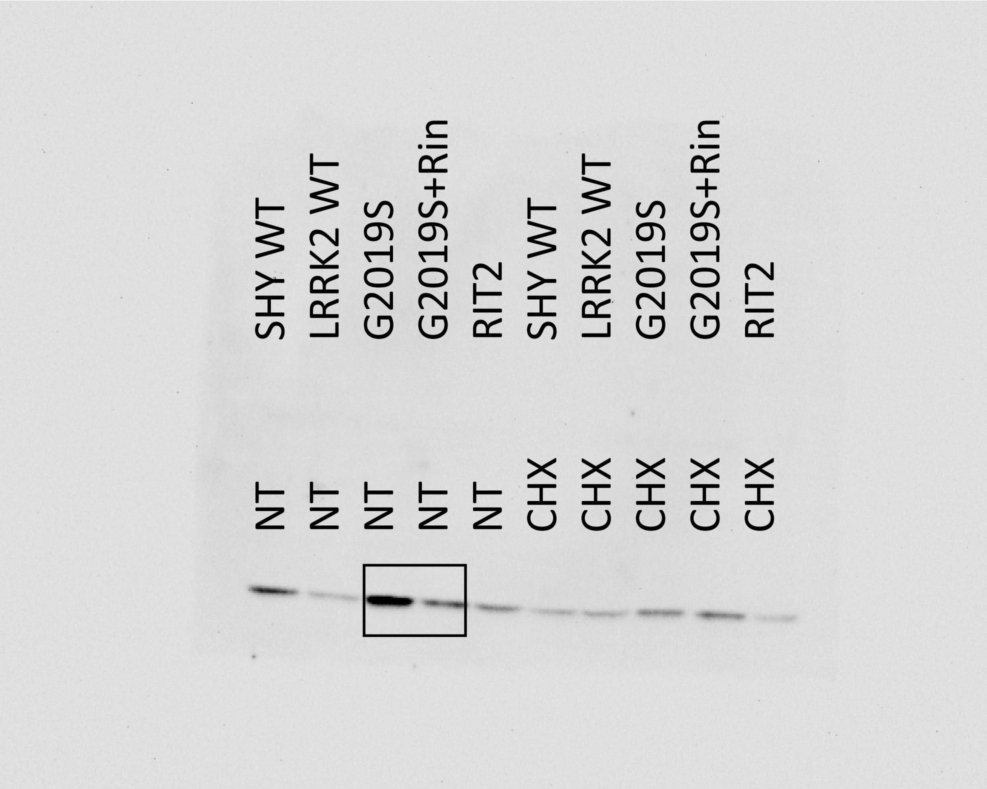

actin

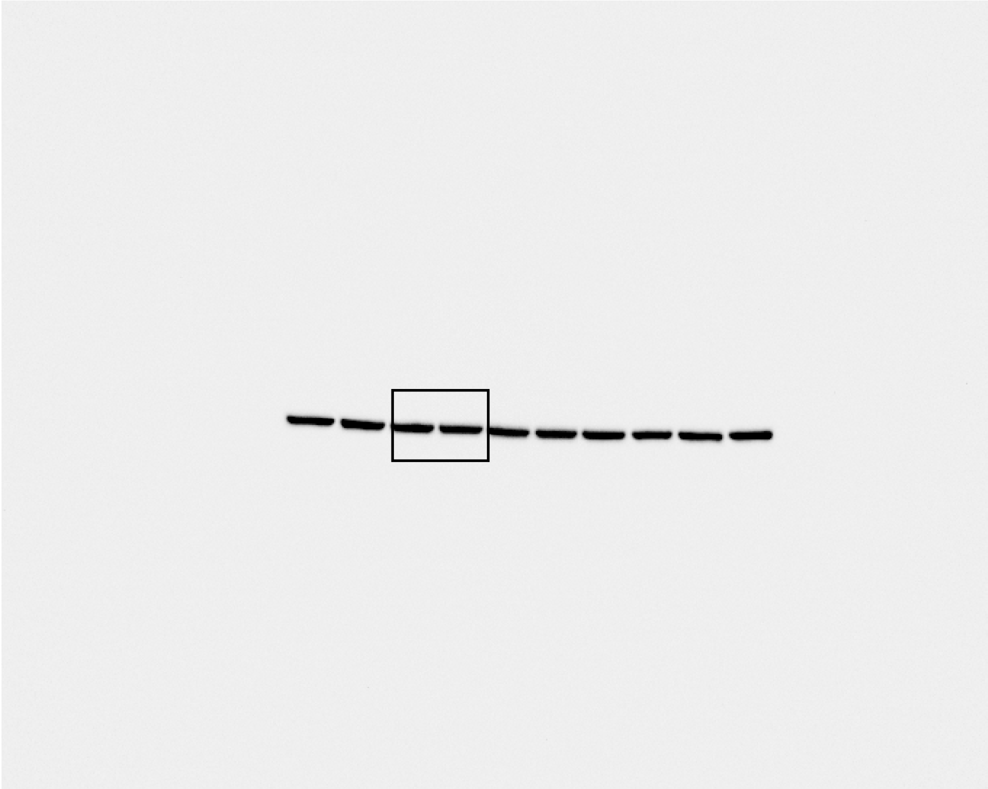

WB for total aSyn (Fig S4)

N6

aSyn

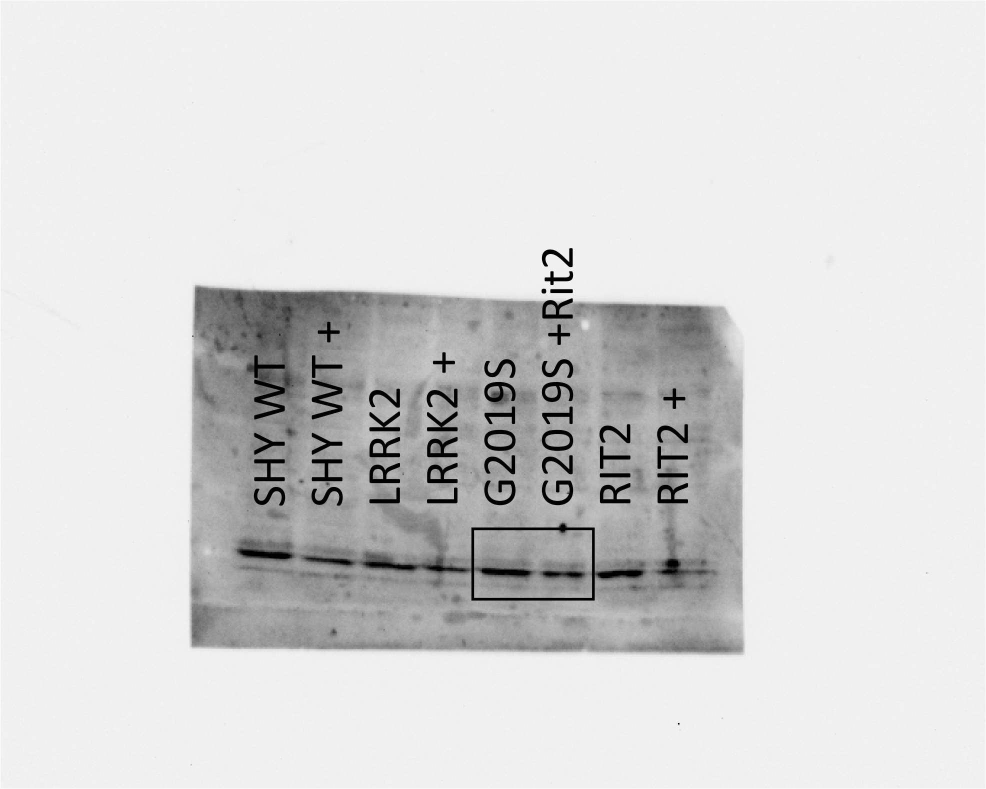

actin

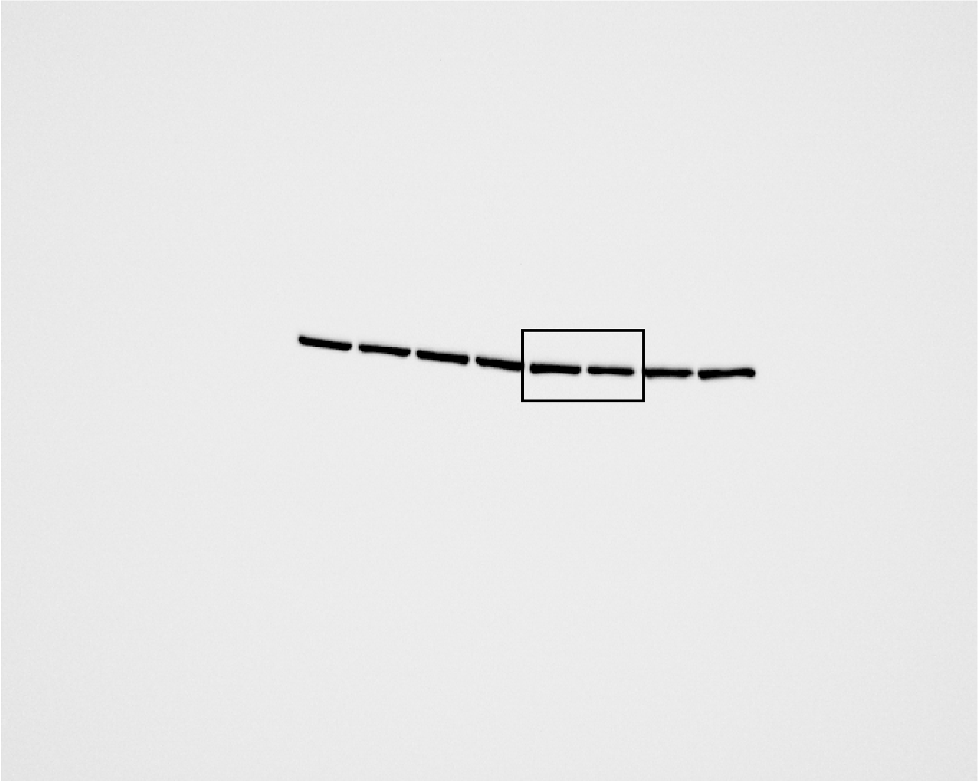

WB for pS1292-LRRK2 (Fig S6)

N1

pSer1292- LRRK2

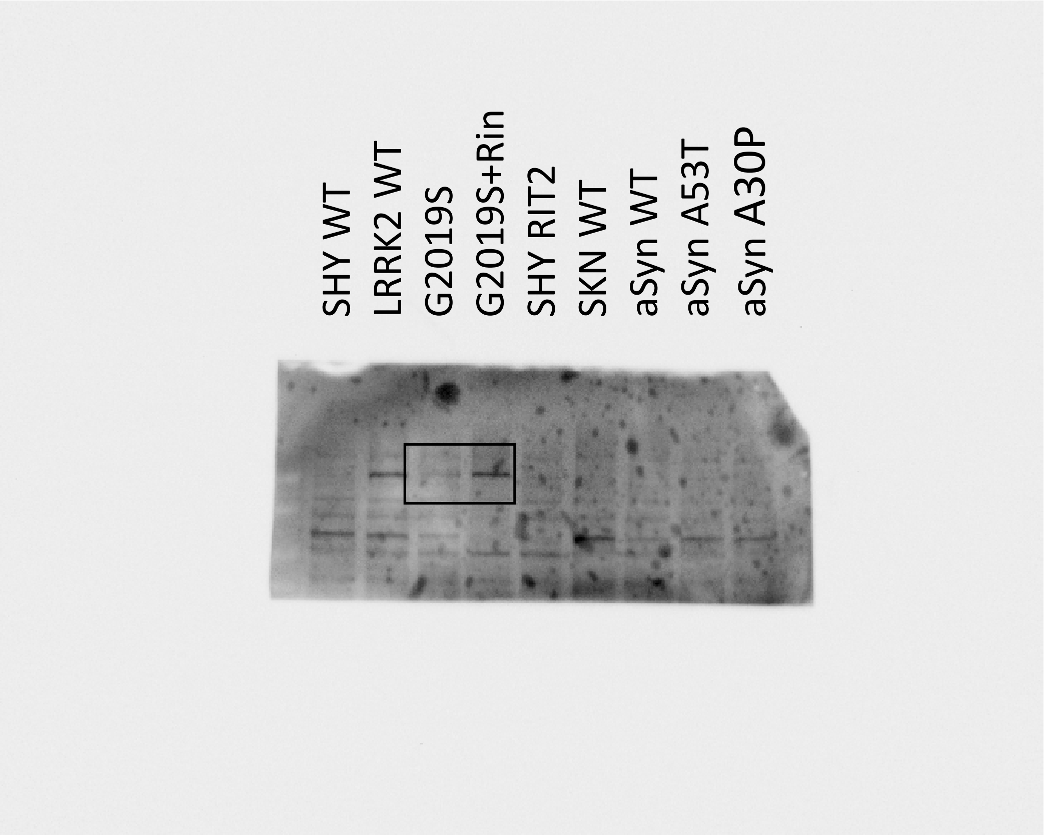

LRRK2

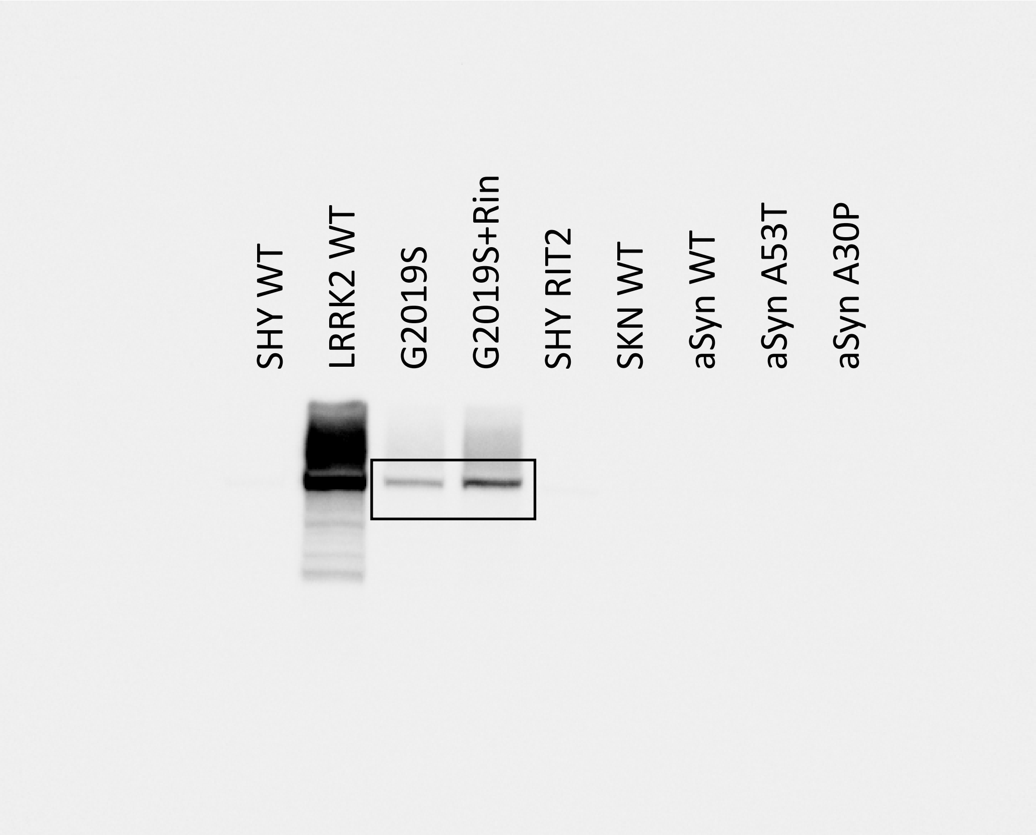

WB for pS1292-LRRK2 (Fig S6)

N2

pSer1292- LRRK2

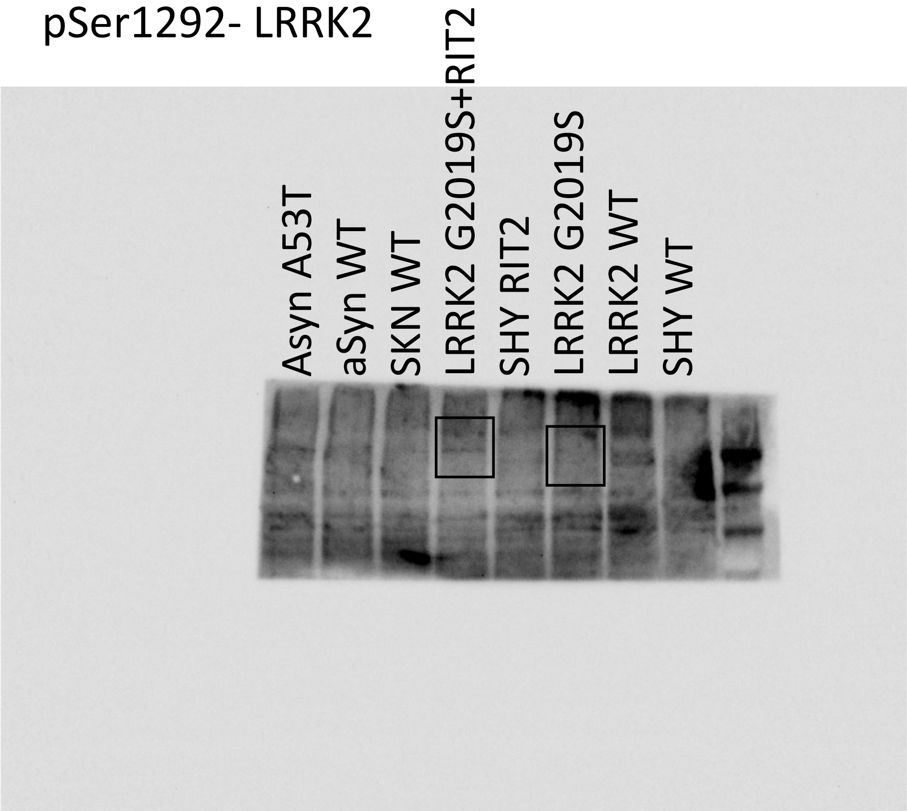

LRRK2

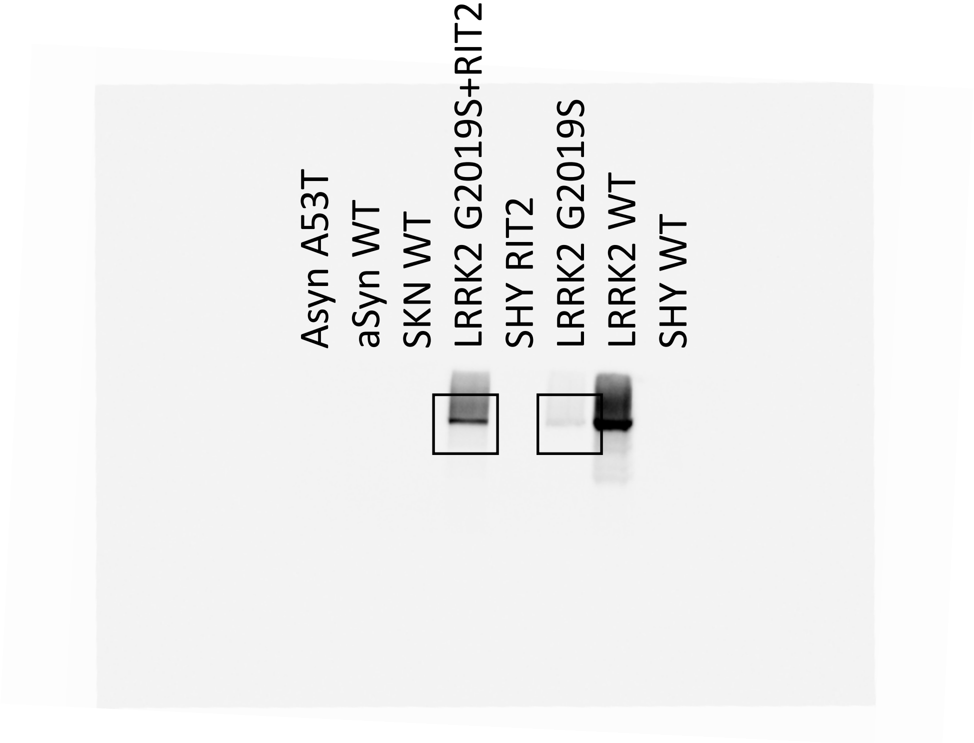

WB for pS1292-LRRK2 (Fig S6)

N3

pSer1292- LRRK2

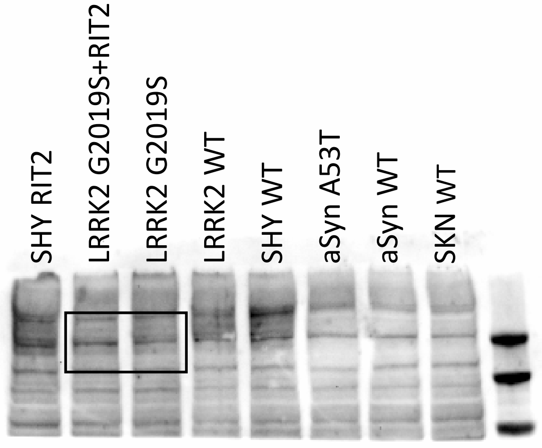

LRRK2

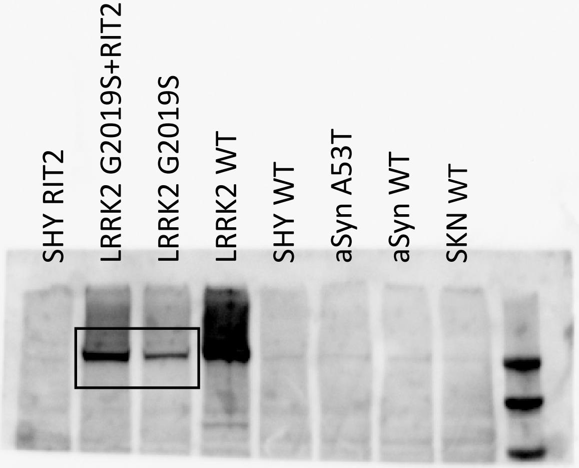

WB for pS1292-LRRK2 (Fig S6)

N4

pSer1292- LRRK2

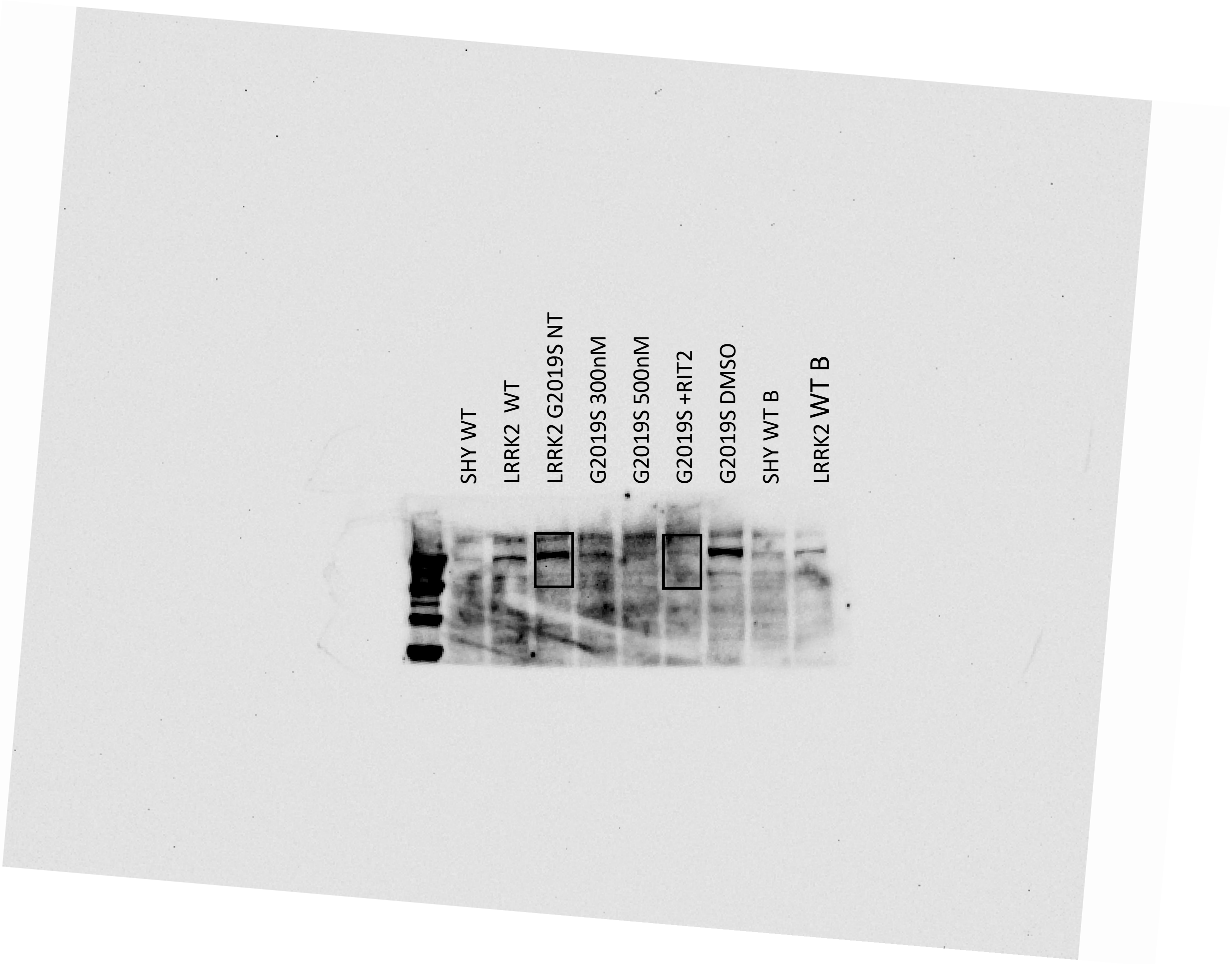

LRRK2

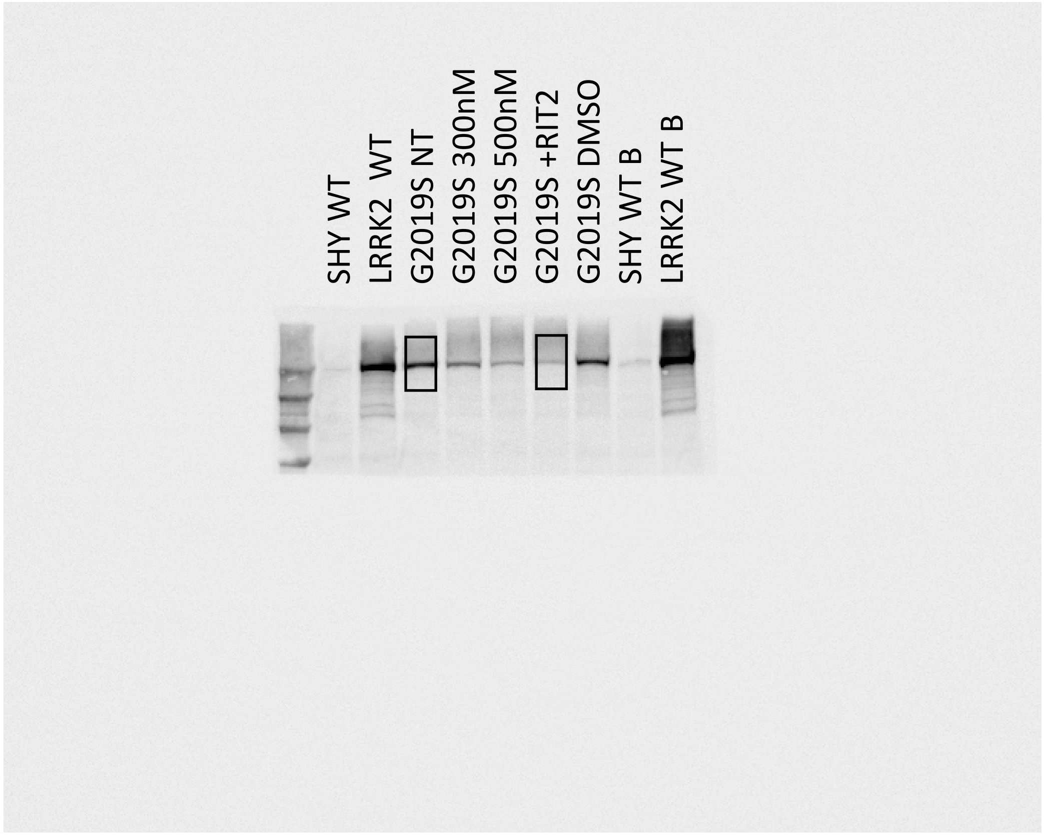

WB for pS935-LRRK2 (Fig S6)

N1, N2, N3

pSer935- LRRK2

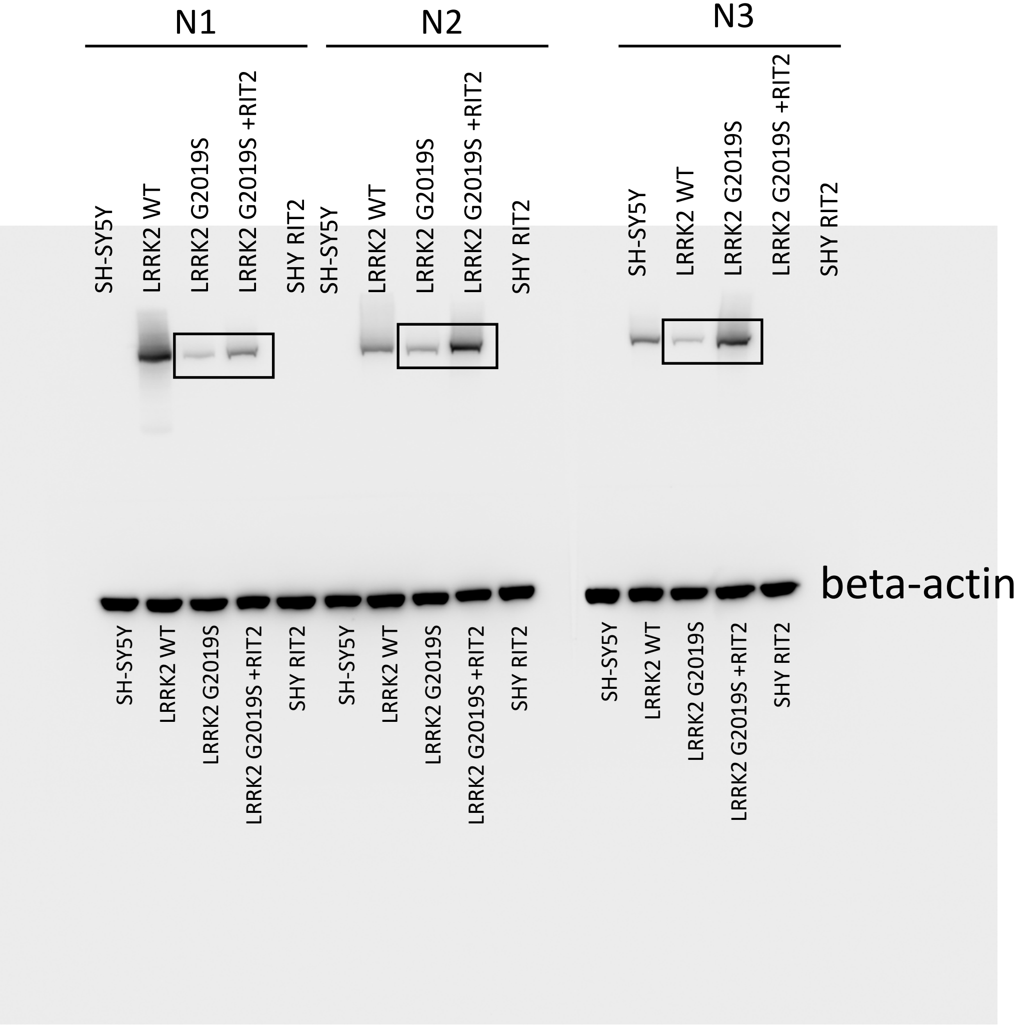

LRRK2

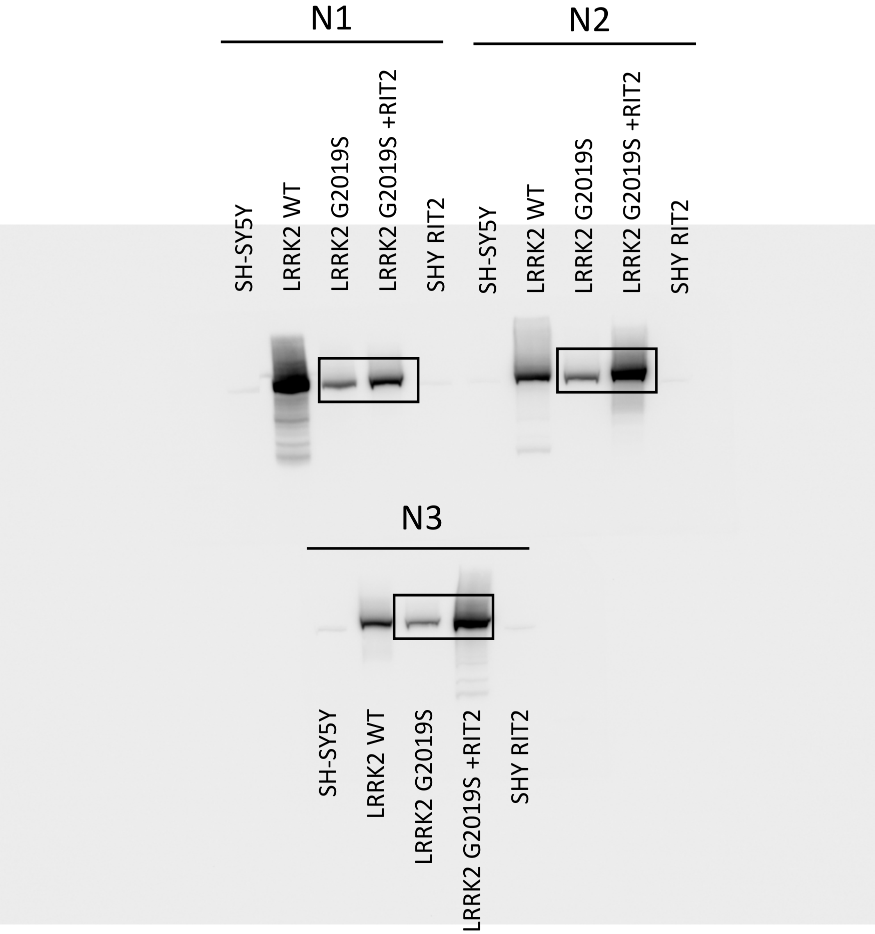

WB for pS935-LRRK2 (Fig S6)

N4, N5

pSer935- LRRK2

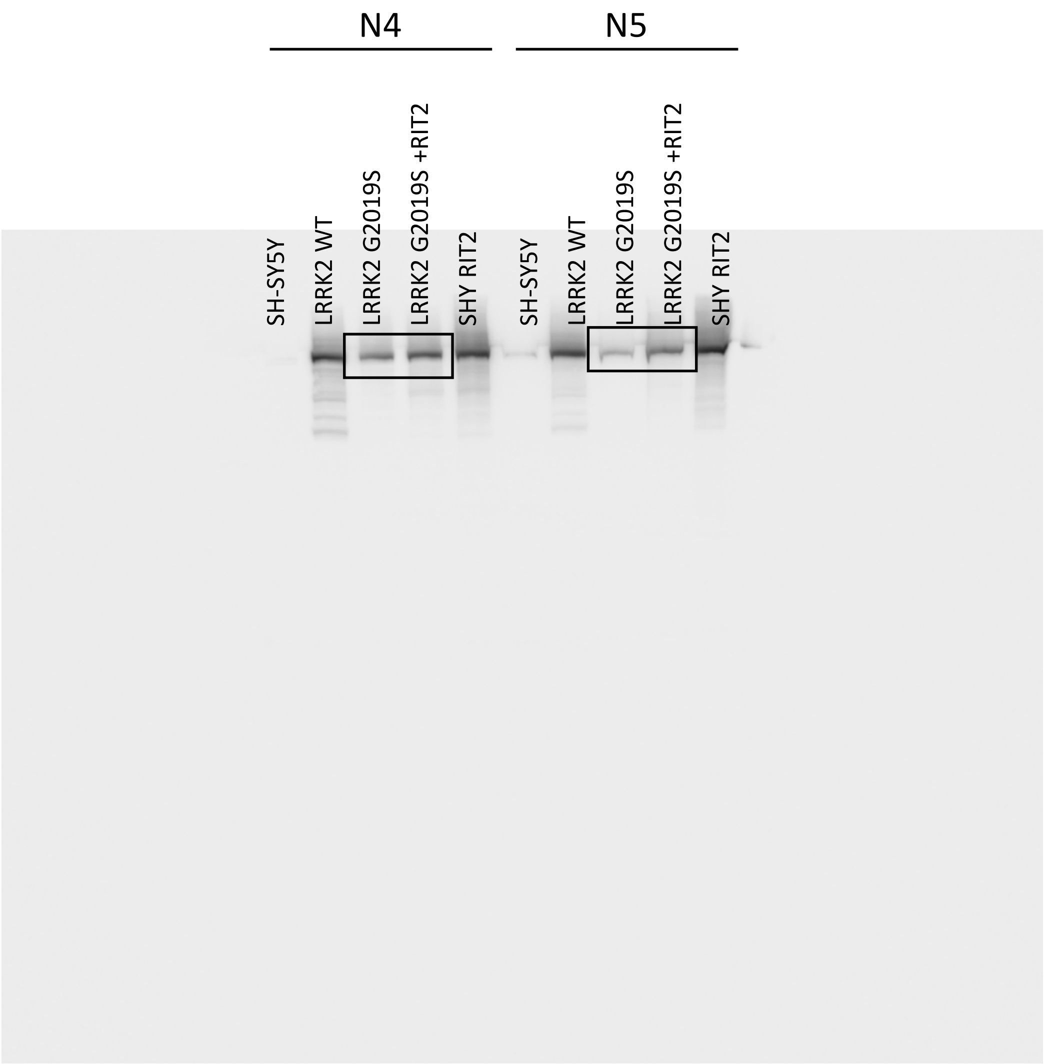

LRRK2

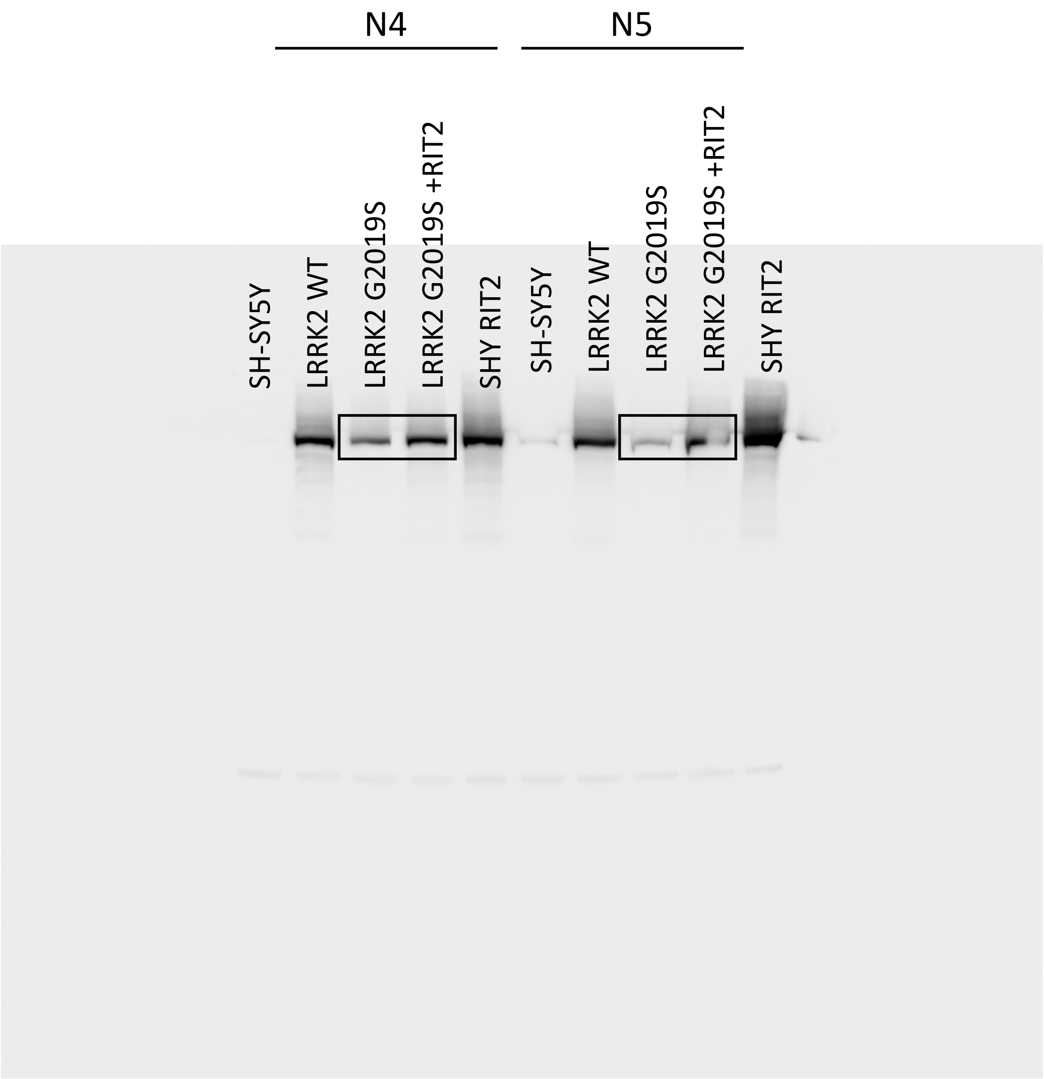

WB for pS935-LRRK2 (Fig S6)

N6

pSer935- LRRK2

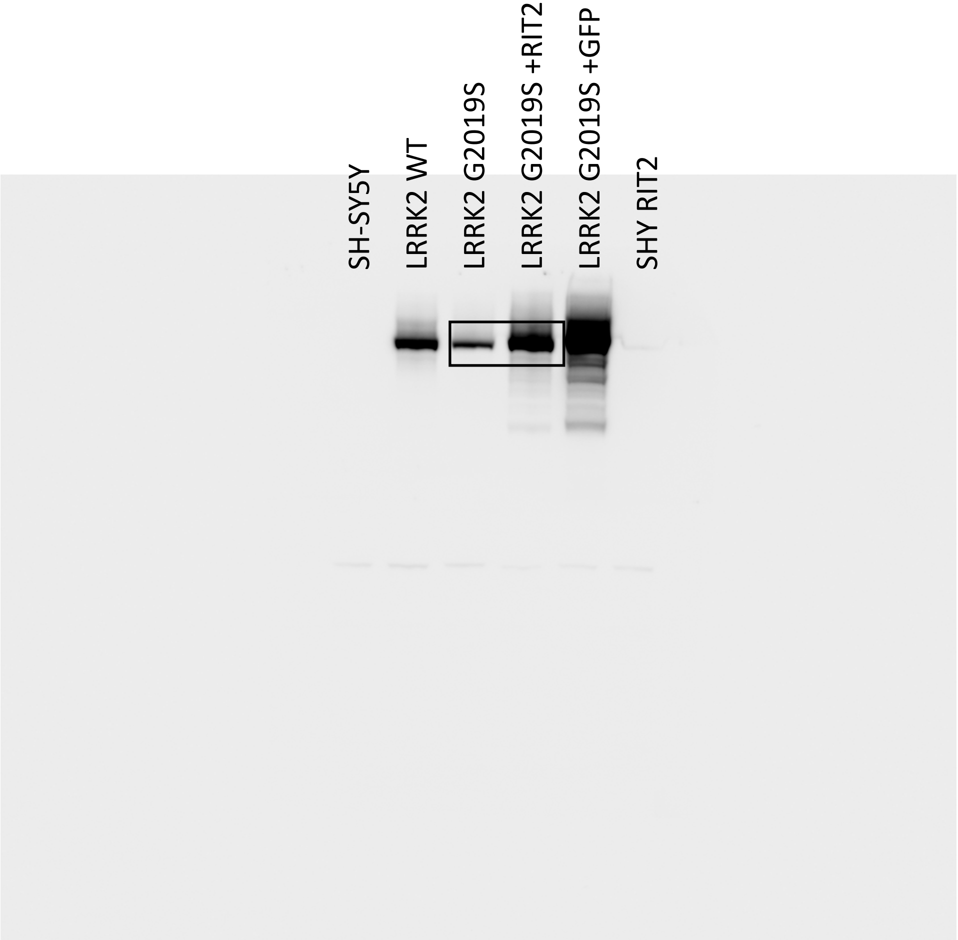

LRRK2

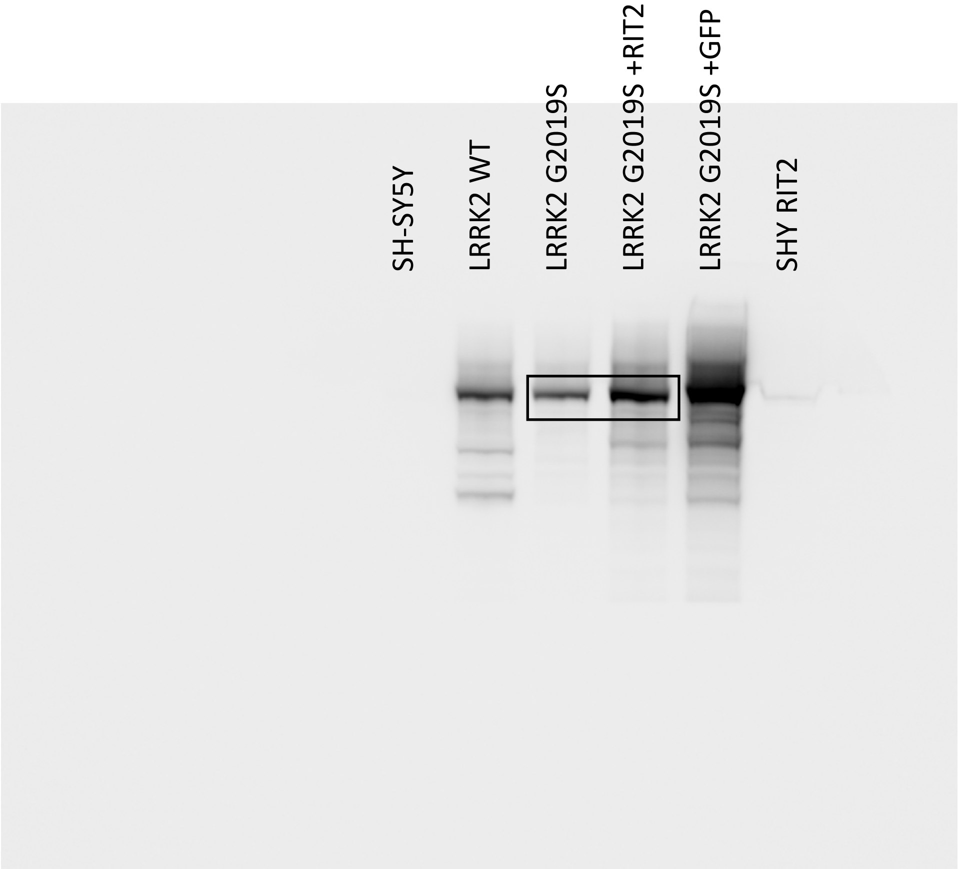

WB for total LRRK2 (Fig S6)

N1, N2, N3

LRRK2

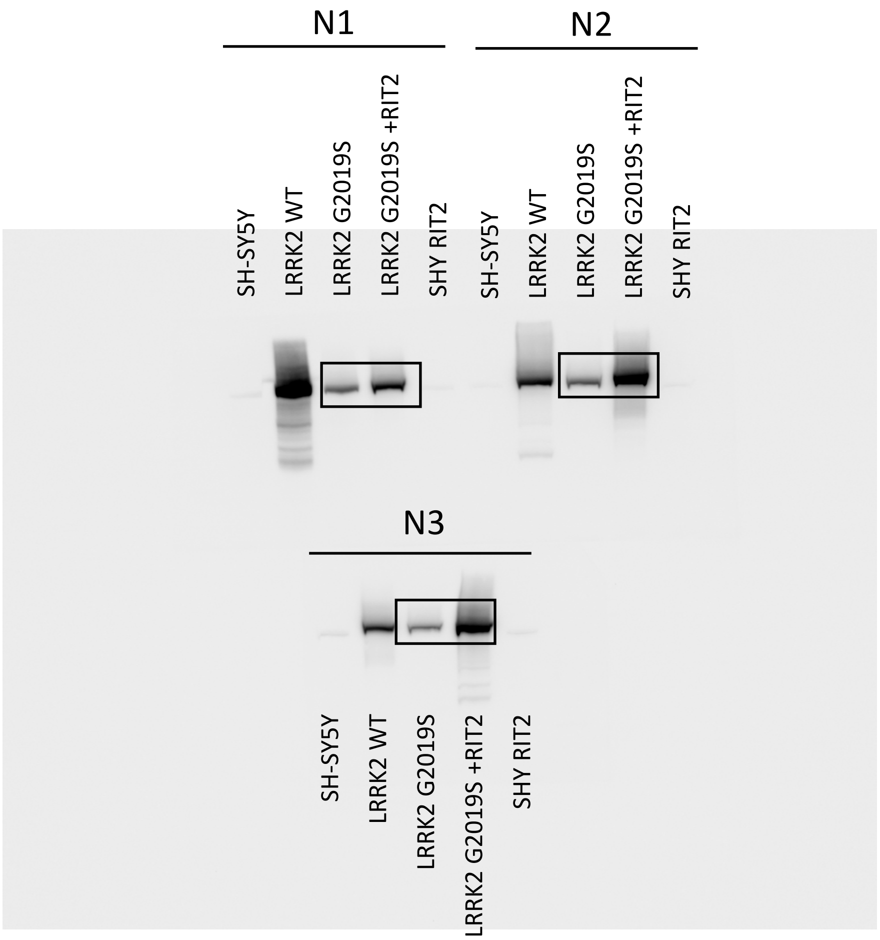

beta-actin

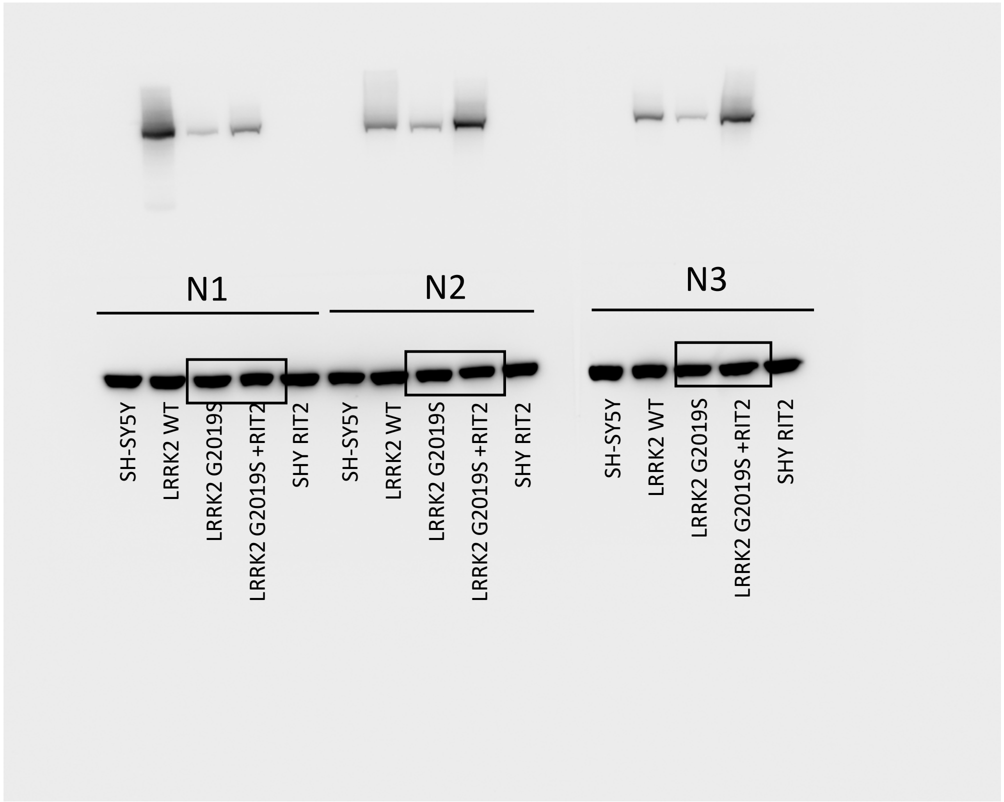

WB for total LRRK2 (Fig S6)
N4, N5

LRRK2

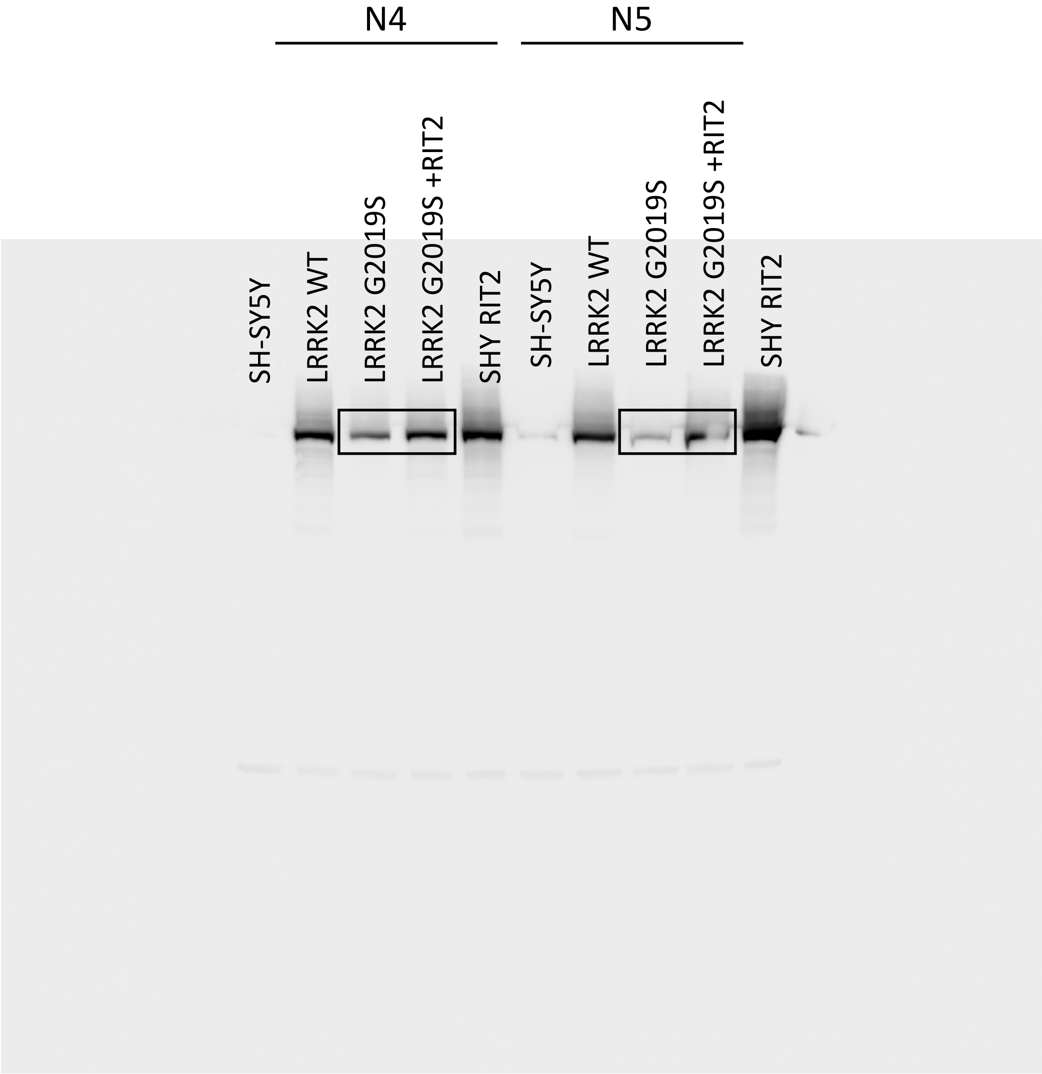

beta-actin

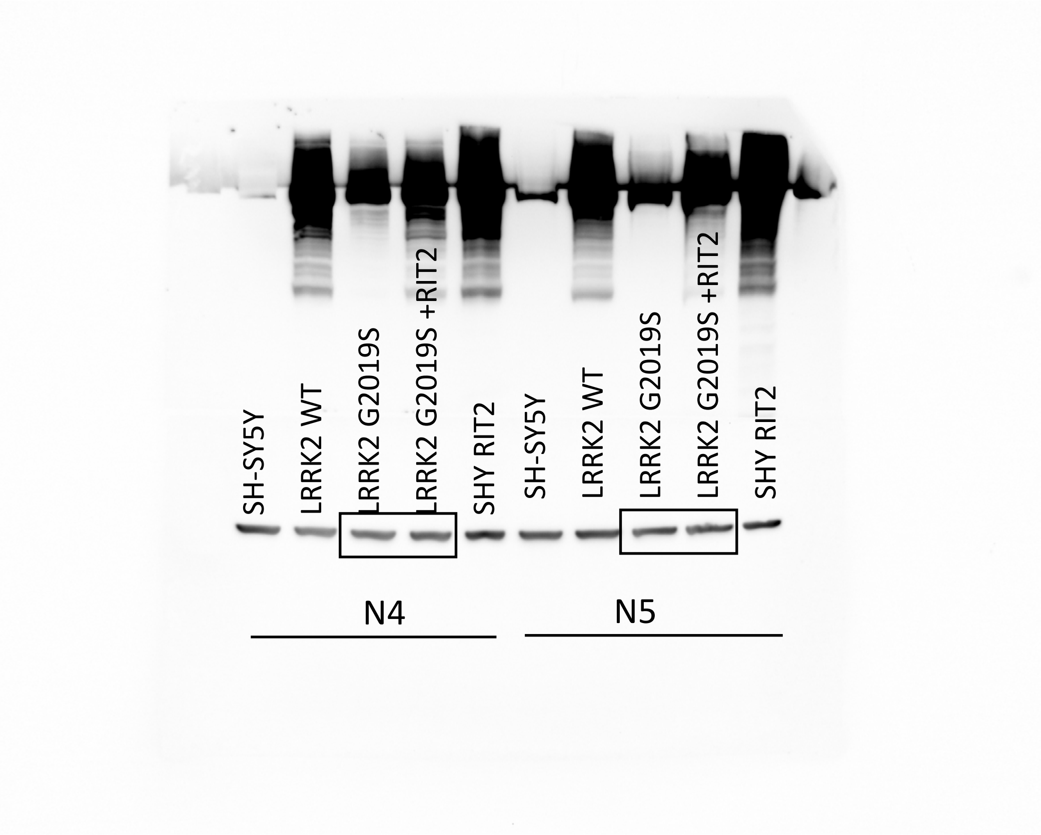

WB for total LRRK2 (Fig S6)

N6

LRRK2

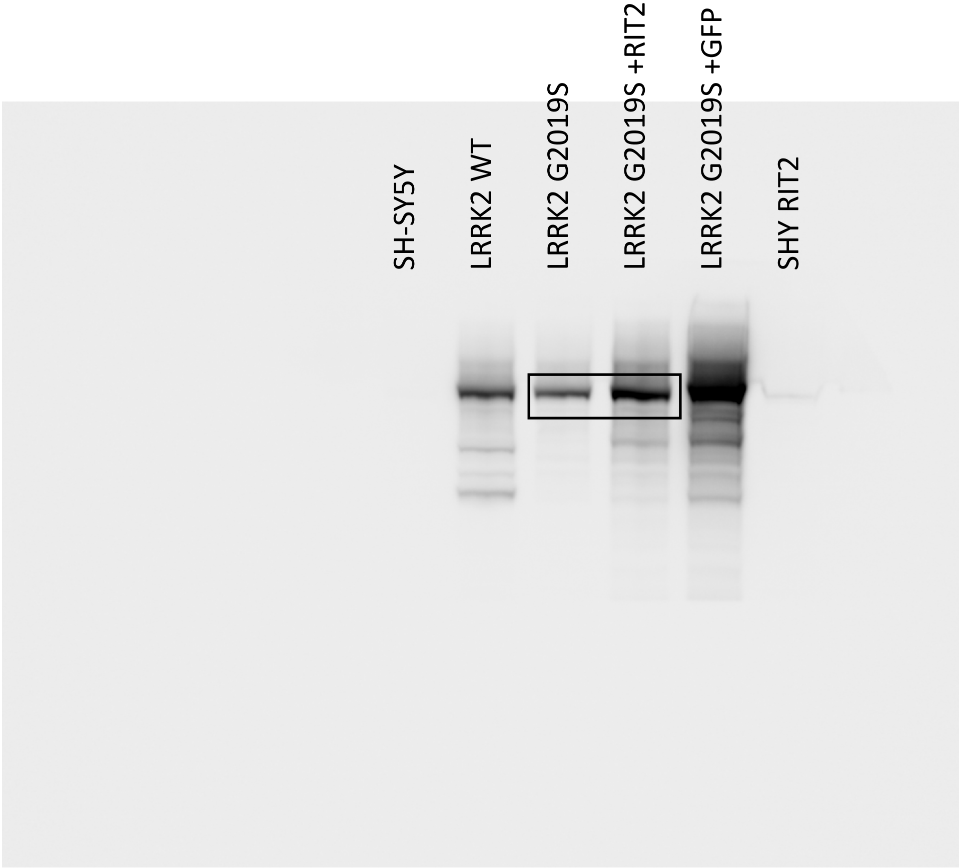

beta-actin

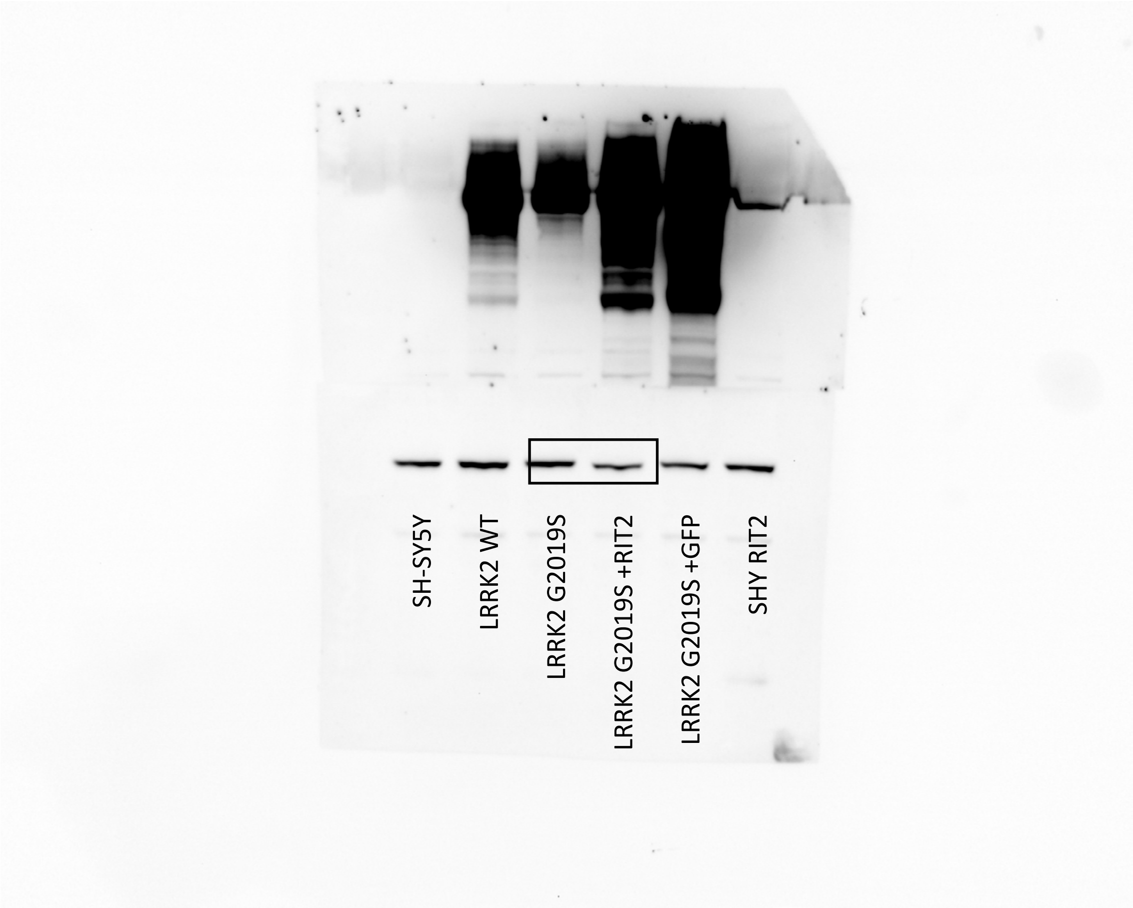

WB for total LRRK2 (Fig S6)

N7

LRRK2

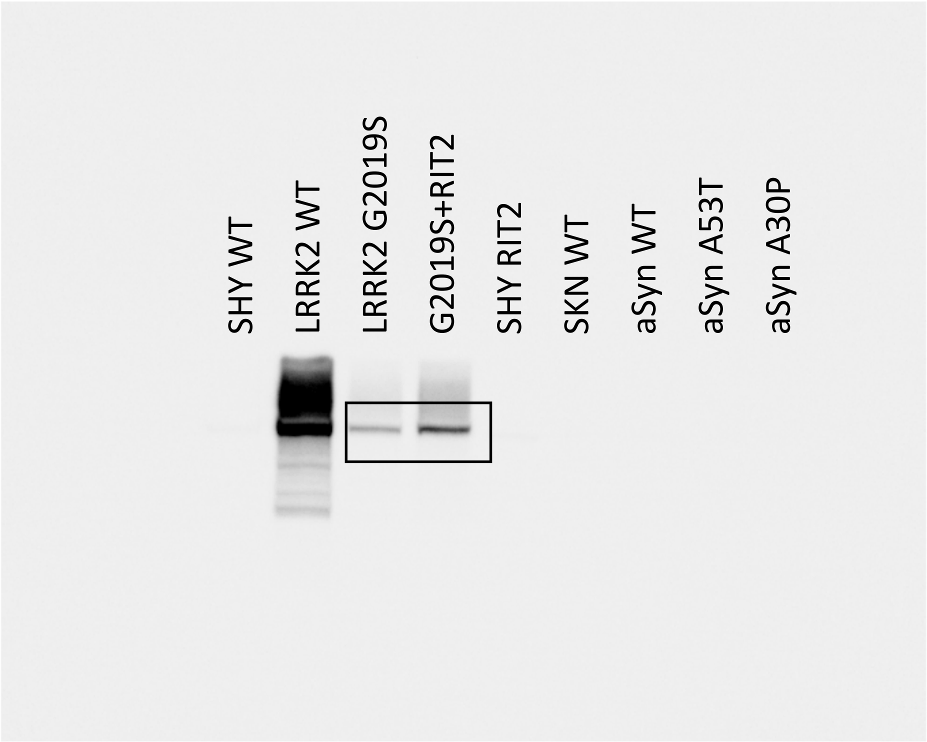

b-actin

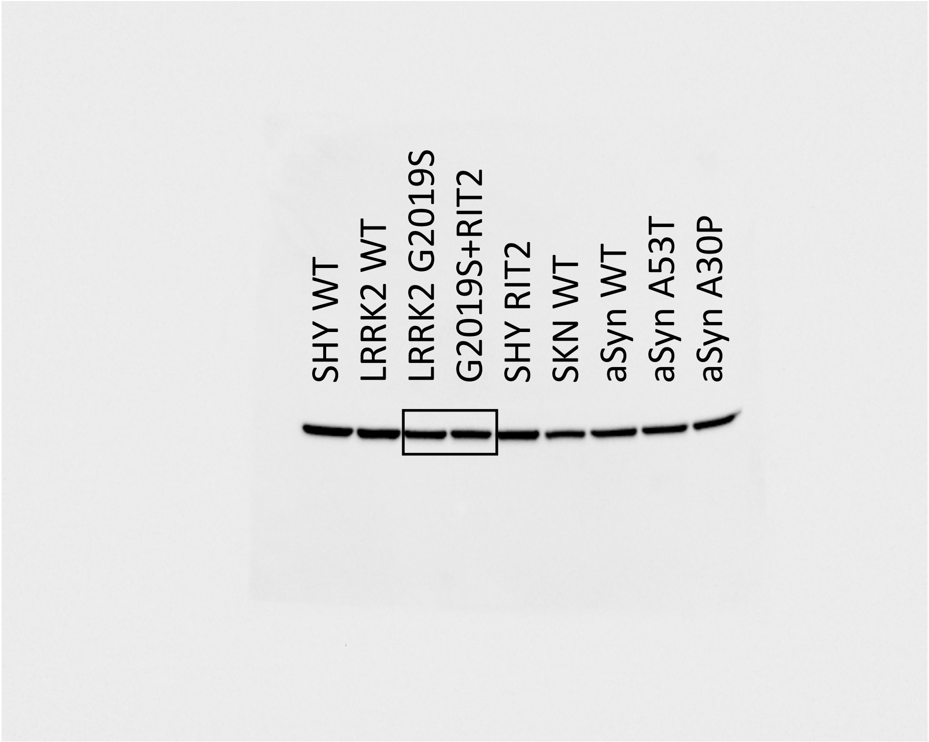

WB for total LRRK2 (Fig S6)

N8

LRRK2

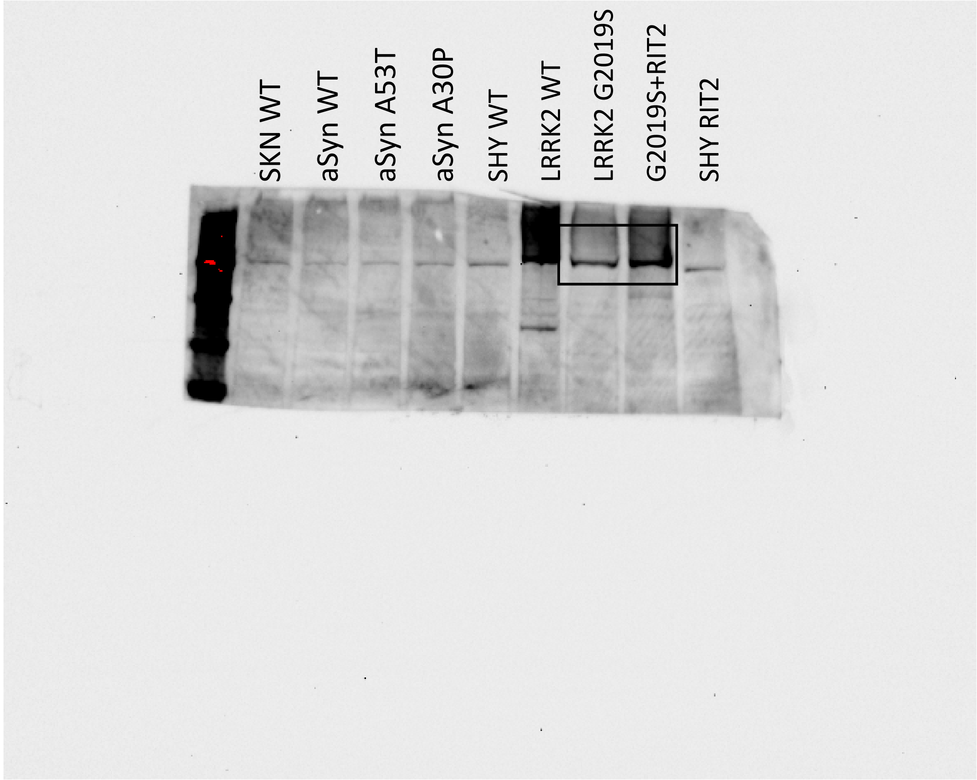

b-actin

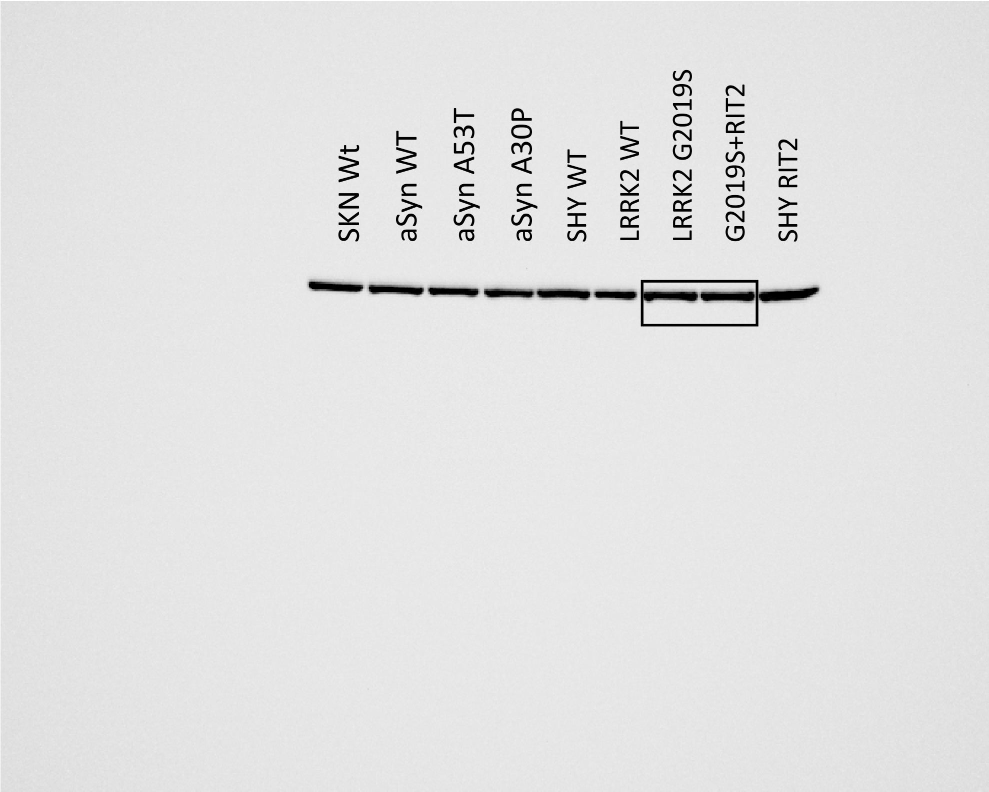

WB for total LRRK2 (Fig S6)

N9

LRRK2

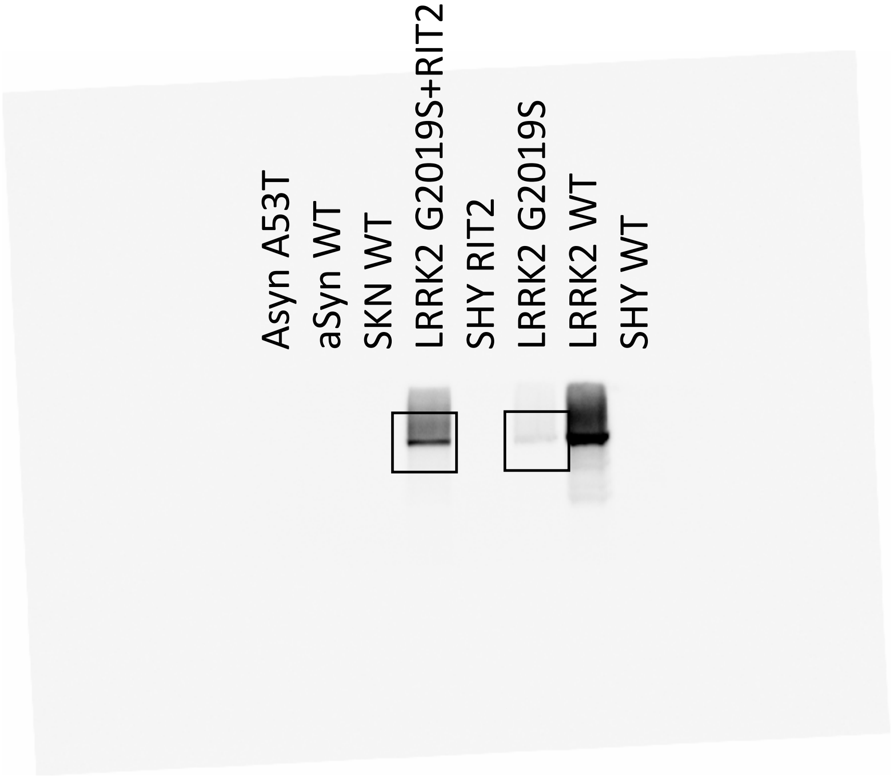

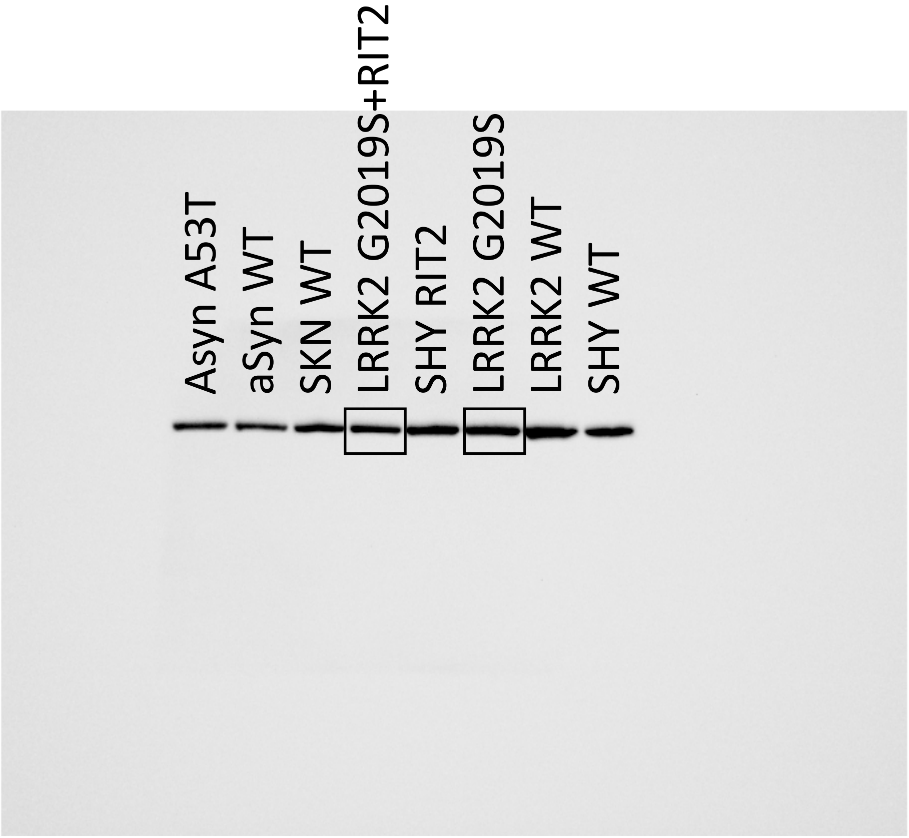

WB for total LRRK2 (Fig S6)

N10

LRRK2

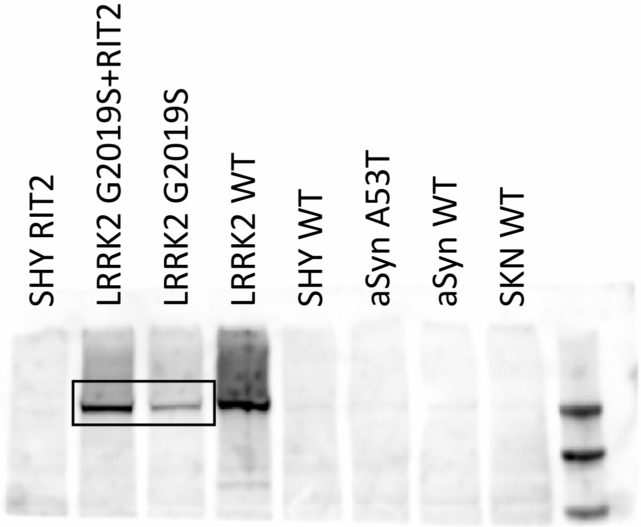

beta-actin

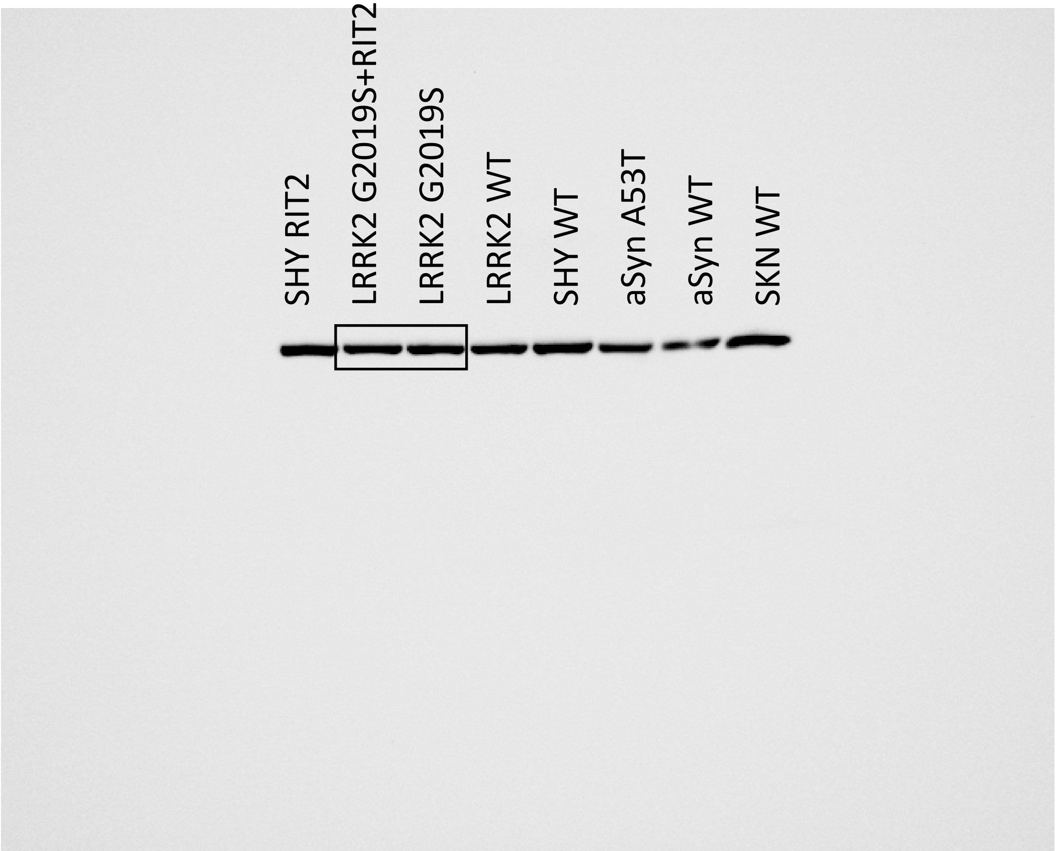

WB for aSyn in vivo (Fig 6)

aSyn

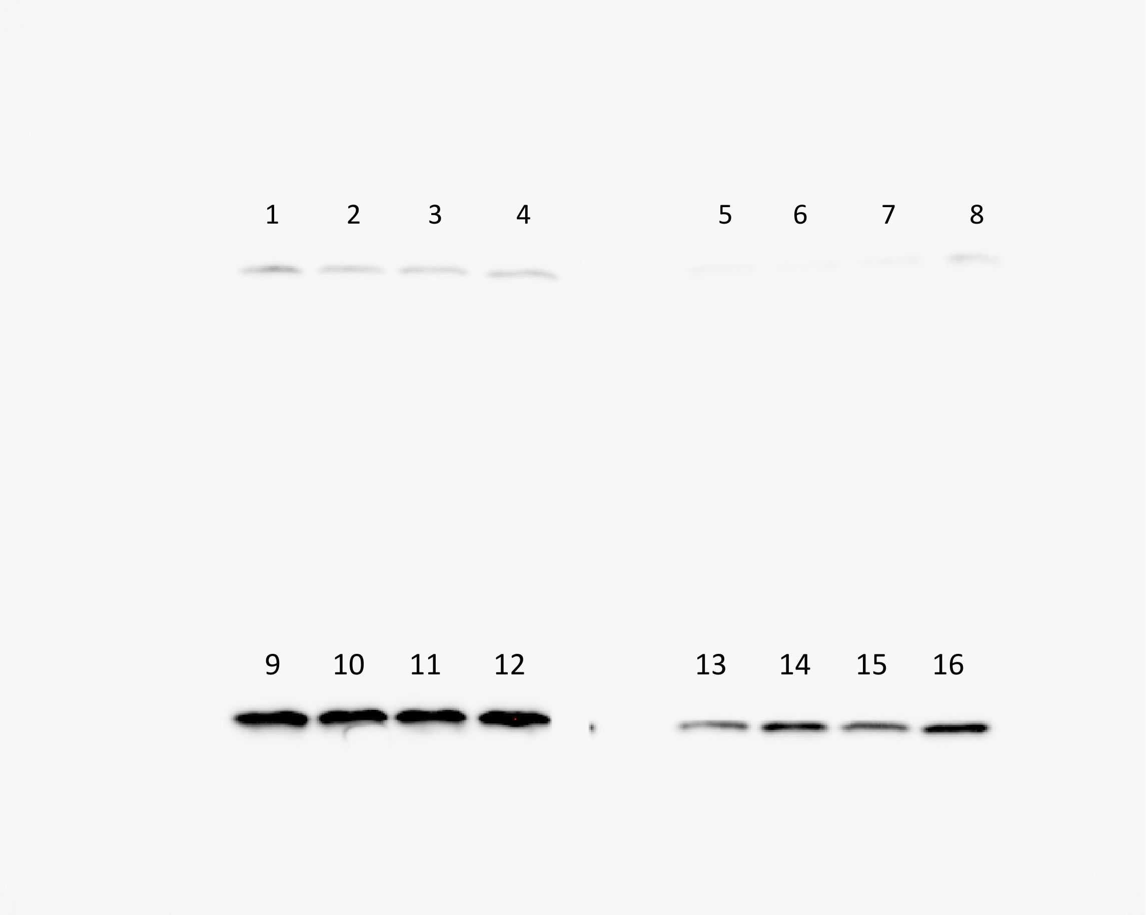

b-actin

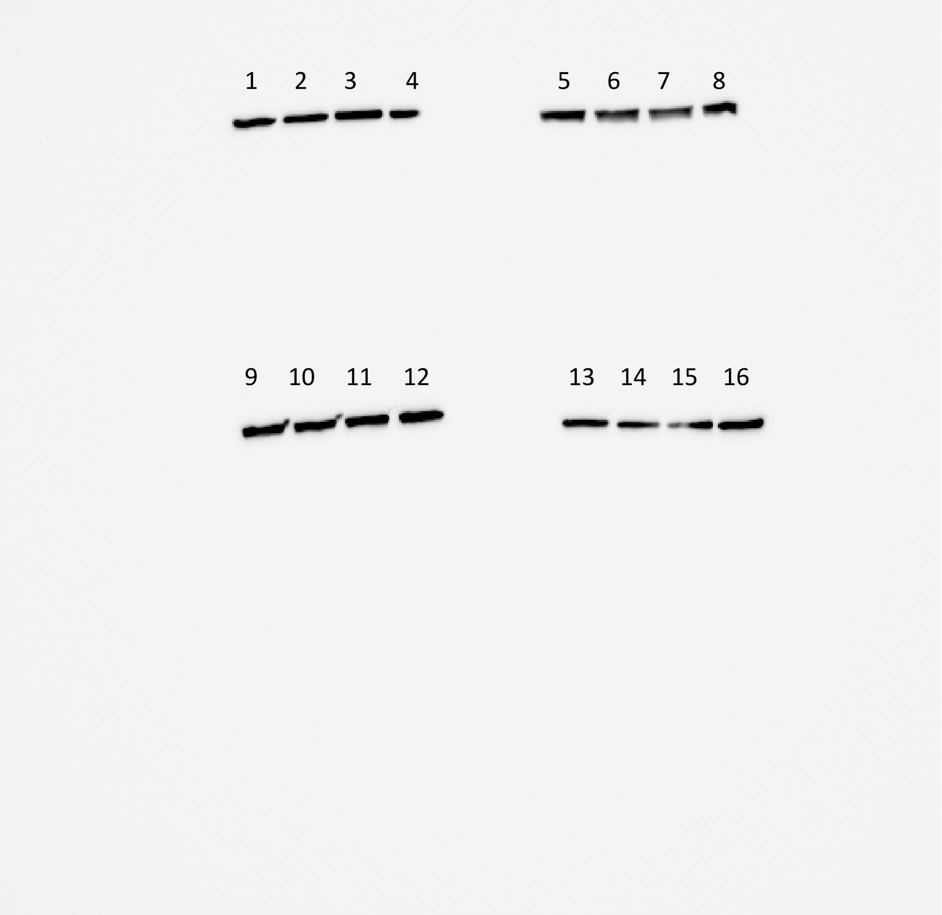

	Mouse ID
AAV-GFP	1
	2
	3
	4
AAV-RIT2	5
	6
	7
	8
AAV-GFP+AAV-aSyn	9
	10
	11
	12
AAV-RIT2+AAV-aSyn	13
	14
	15
	16

WB for aSyn in vivo (Fig 6)

pS129-aSyn

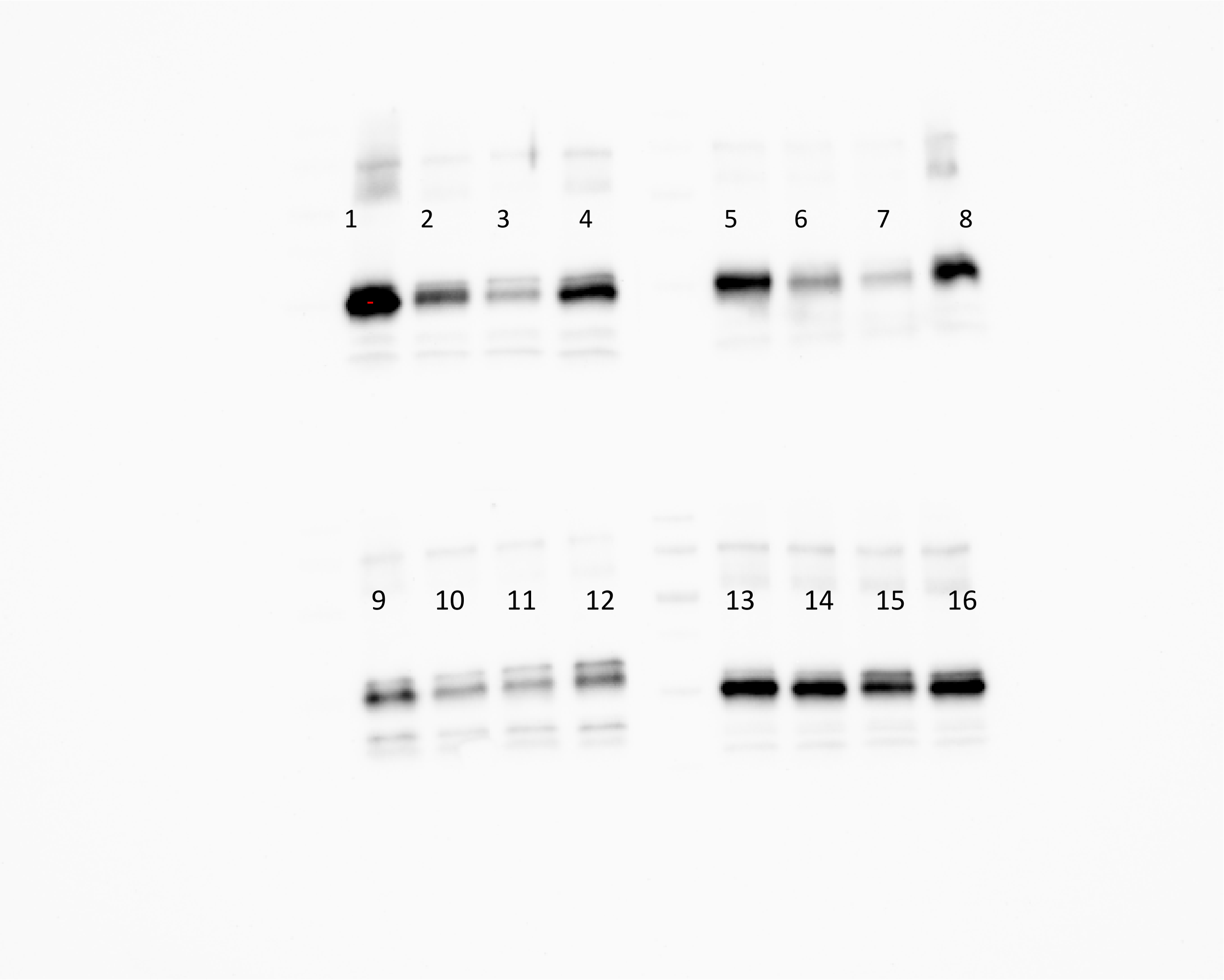

WB for LRRK2, Rit2 in vivo (Fig S8)

LRRK2

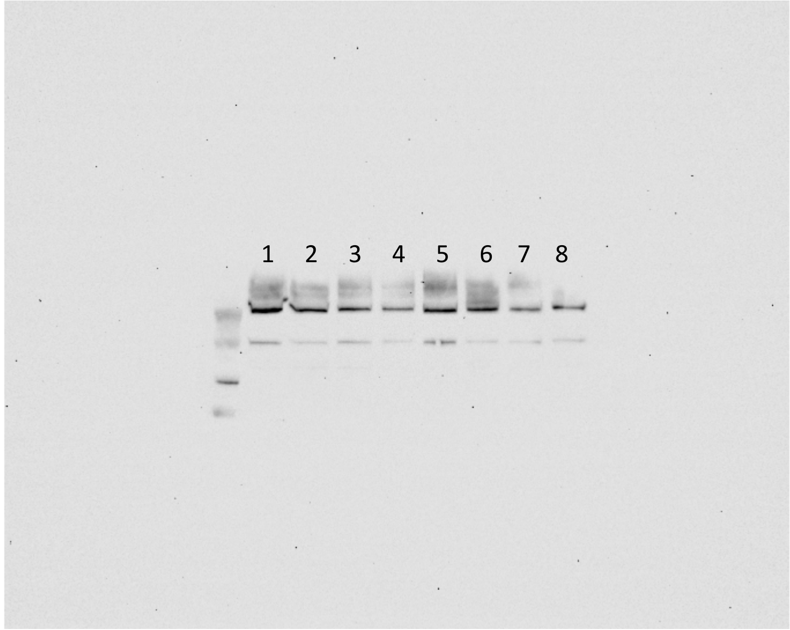

b-actin

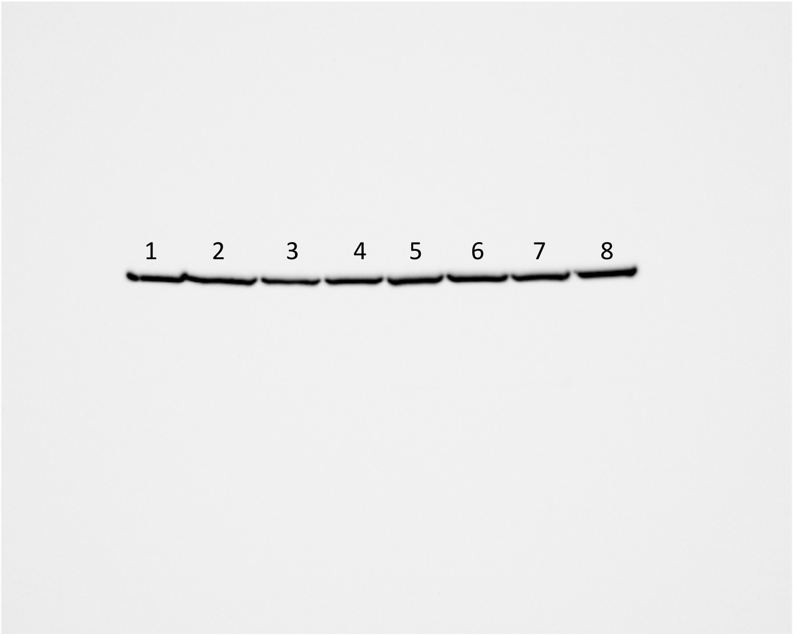

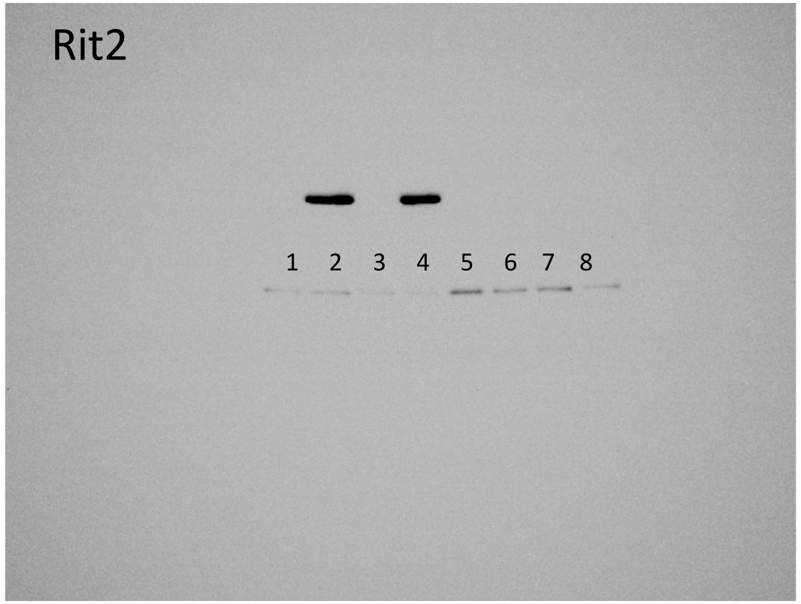

LC3

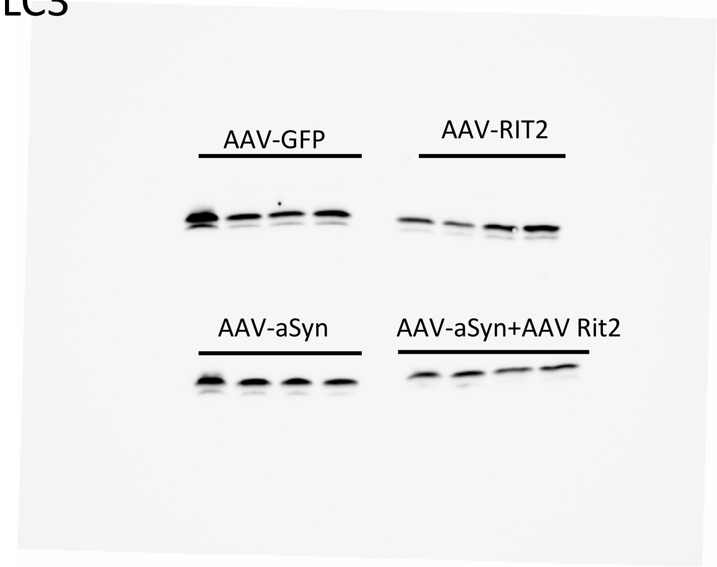

1	aSyn+RIT2	244 G
2		244 D
3		240 G
4		240 D
5	aSyn+GFP	245 G
6		245 D
7		189 D
8		189 G

G=contralateral
D= ipsilateral

WB for LRRK2 in vivo (Fig S8)

LRRK2

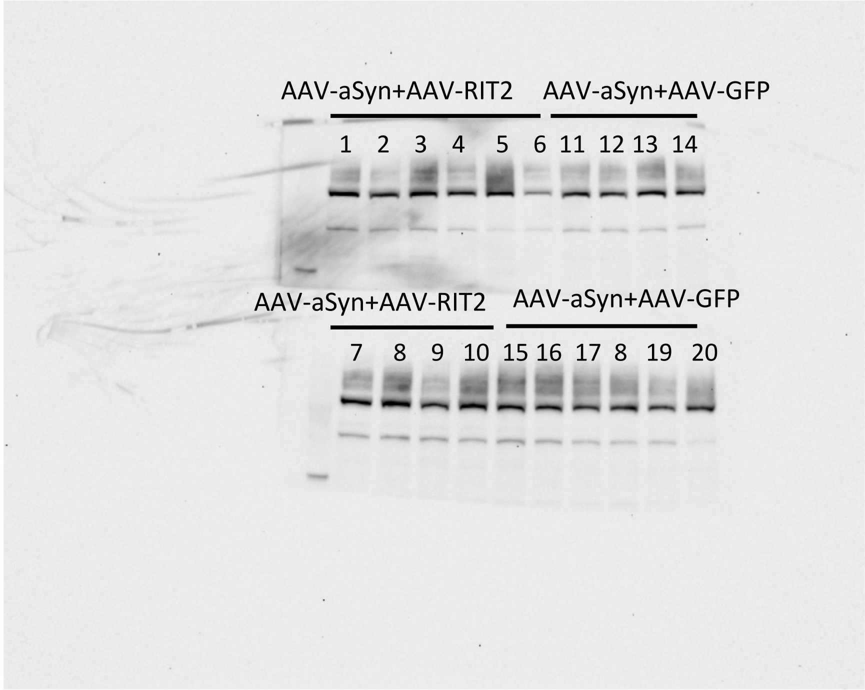

b-actin

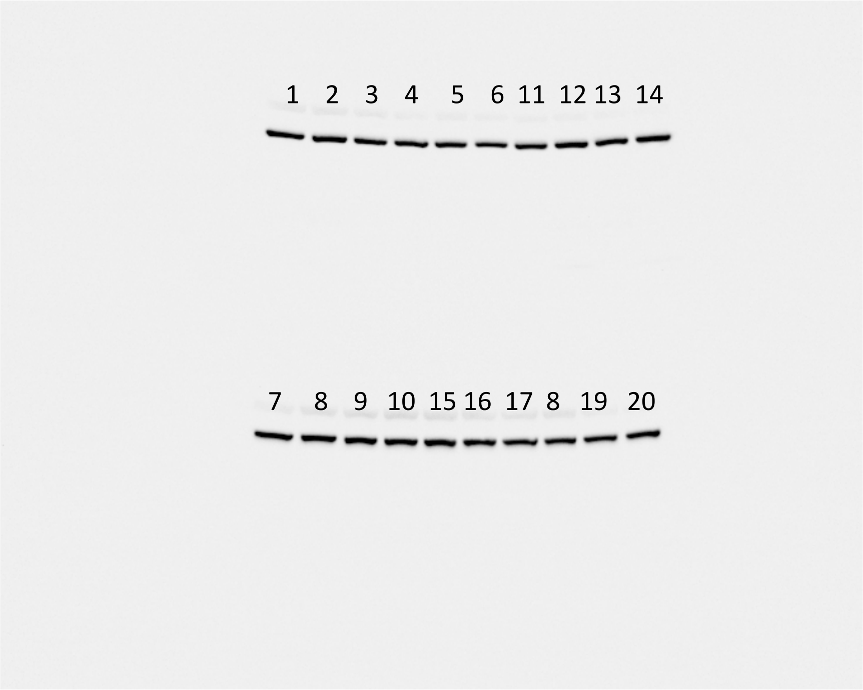

aSyn+RIT2

19244	G	1
19244	D	2
19240	G	3
19240	D	4
19191	G	5
19191	D	6
19192	G	7
19192	D	8
19241	G	9
19241	D	10

aSyn+GFP

19190	G	11
19190	D	12
19246	G	13
19246	D	14
19245	G	15
19245	D	16
19189	G	17
19189	D	18
19248	G	19
19248	D	20

G=contralateral
D= ipsilateral

WB total LRRK2, total aSyn in WT LRRK2 cells (Fig S3) and Rit2-KO cells (Fig S9)

WB 05-09-22

Total LRRK2

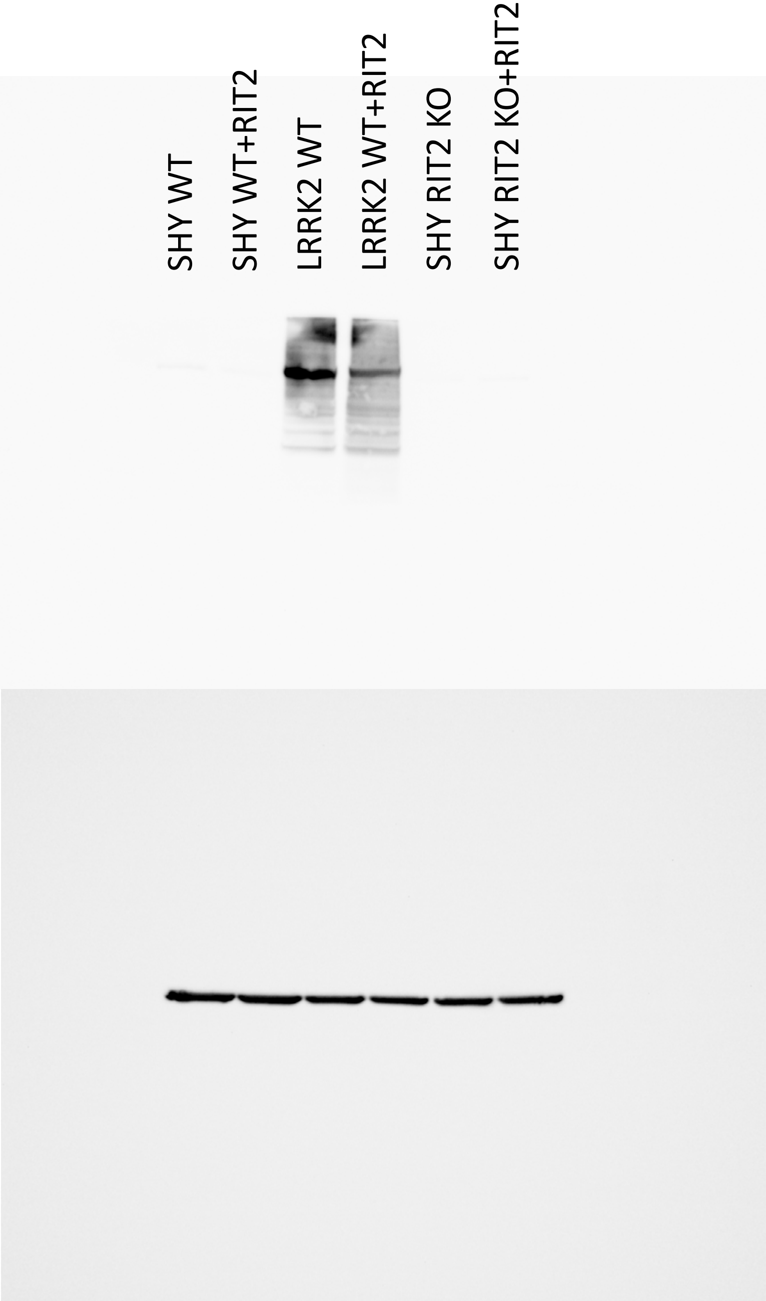

Beta-actin

Total aSyn

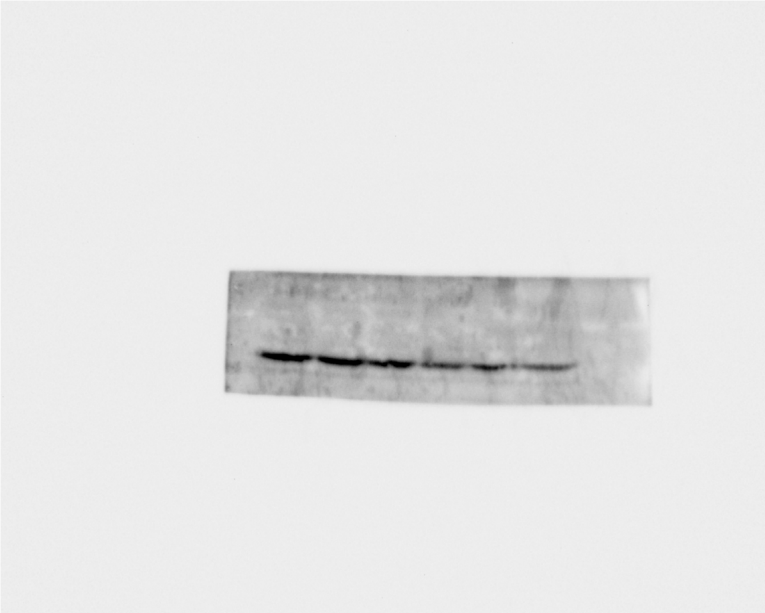

WB 19-09-22

WB total LRRK2, total aSyn in WT LRRK2 cells (Fig S3) and Rit2-KO cells (Fig S9)

Total LRRK2

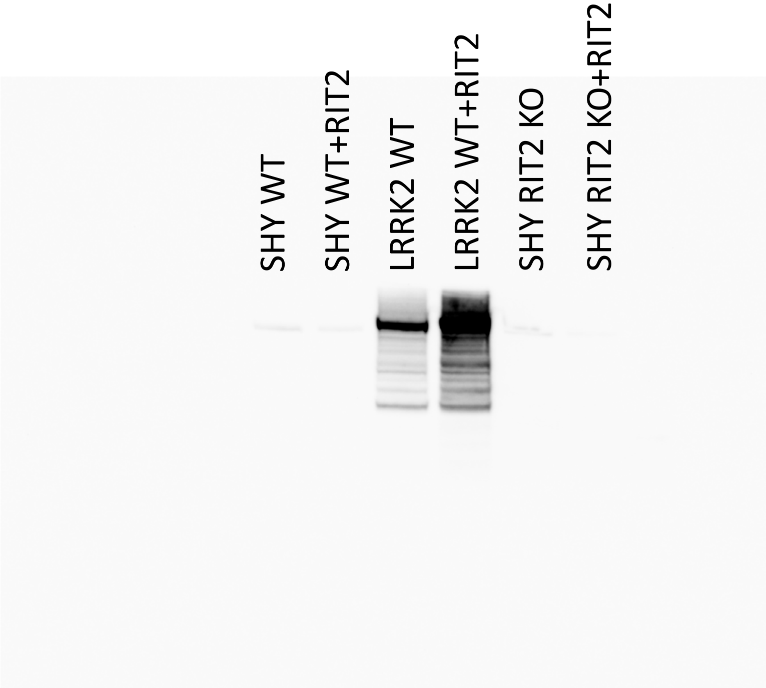

Total aSyn

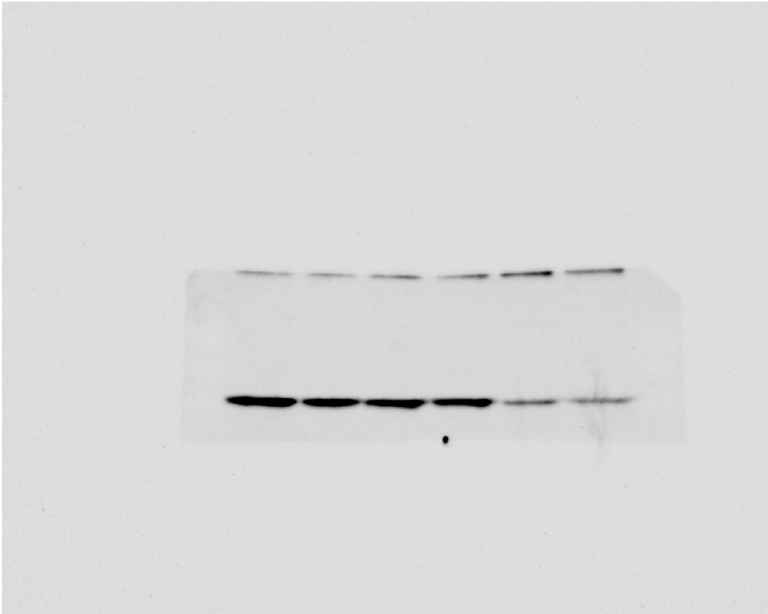

Beta-actin

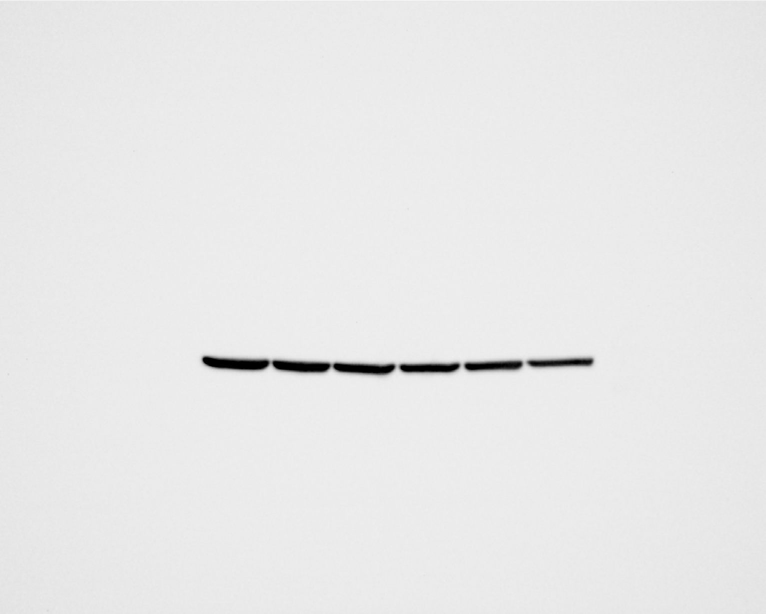

WB total LRRK2, total aSyn in WT LRRK2 cells (Fig S3) and Rit2-KO cells (Fig S9)

WB 22-09-22

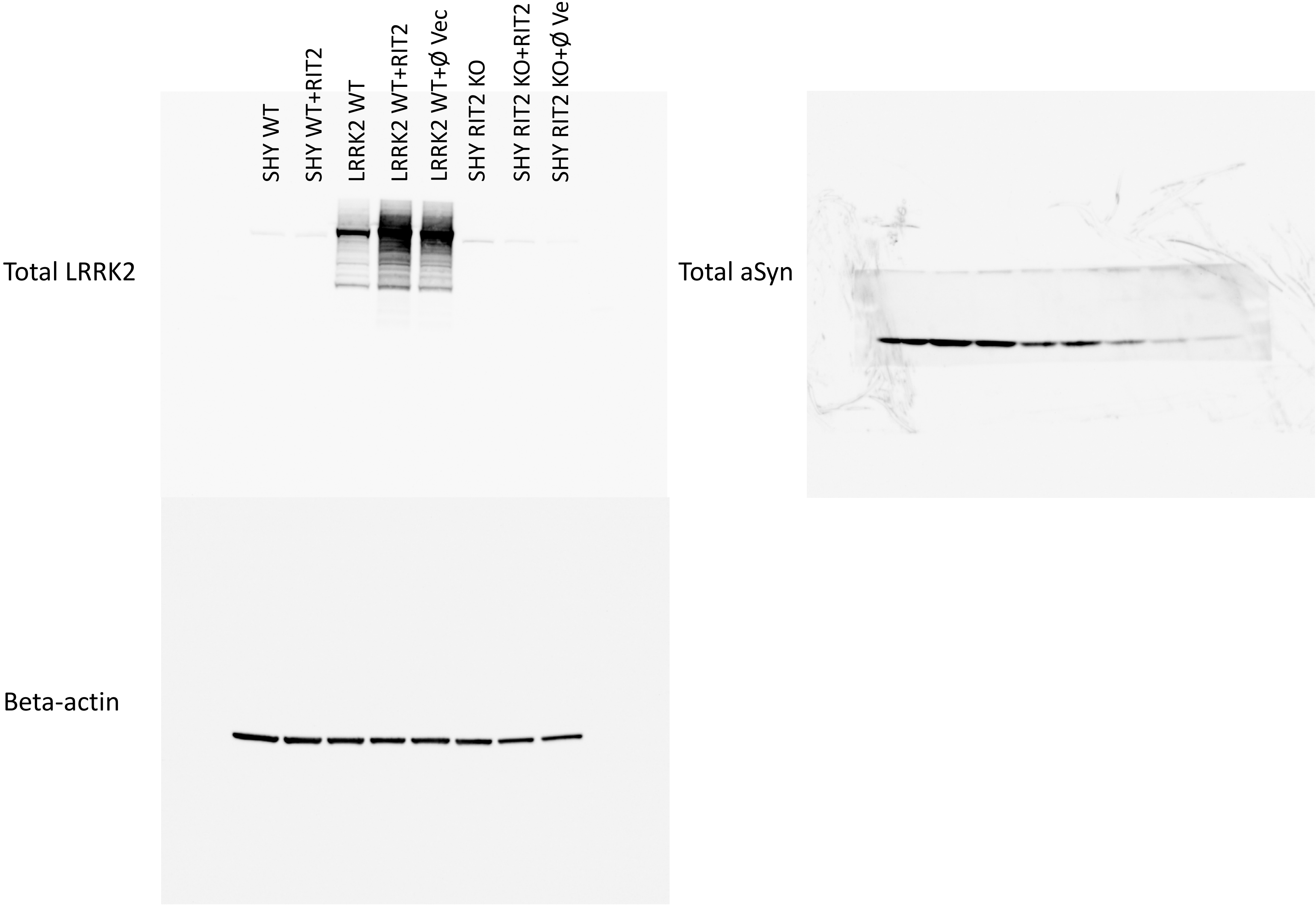

WB total LRRK2, total aSyn in WT LRRK2 cells (Fig S3) and Rit2-KO cells (Fig S9)

WB 28-09-22

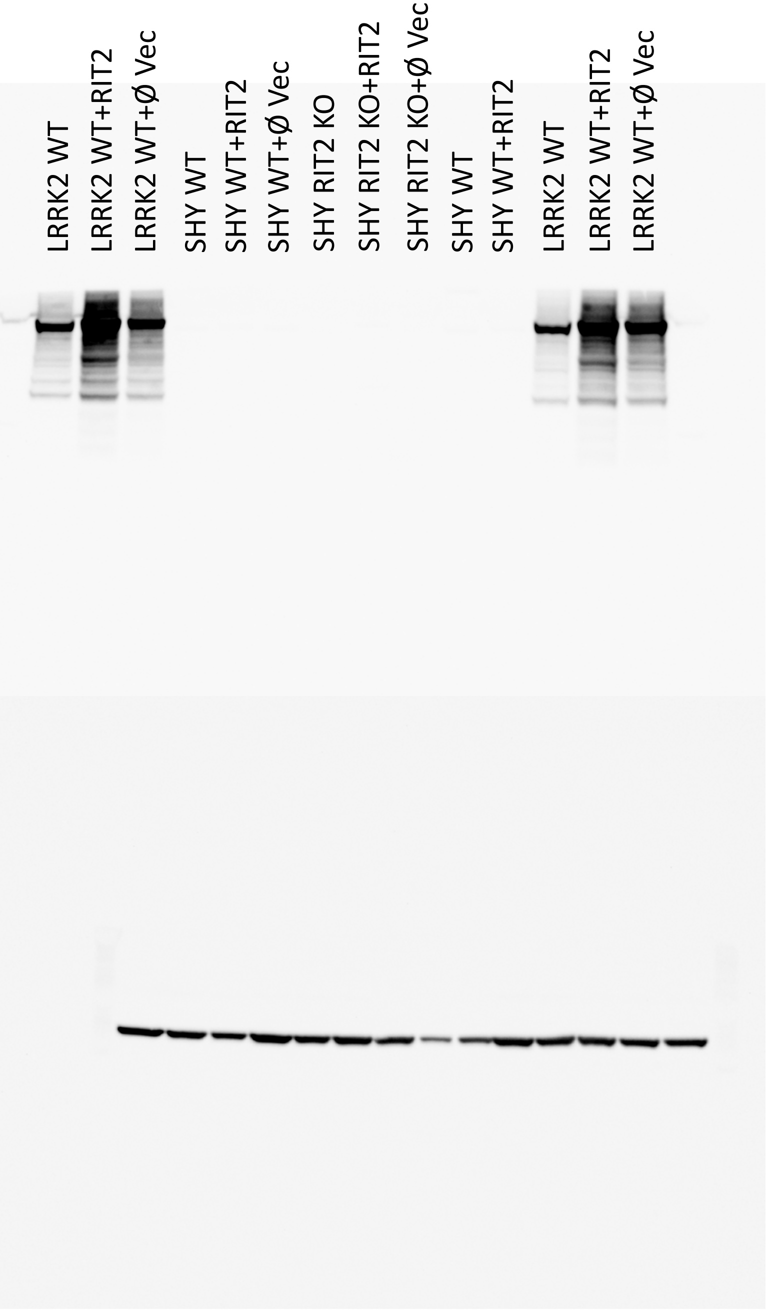

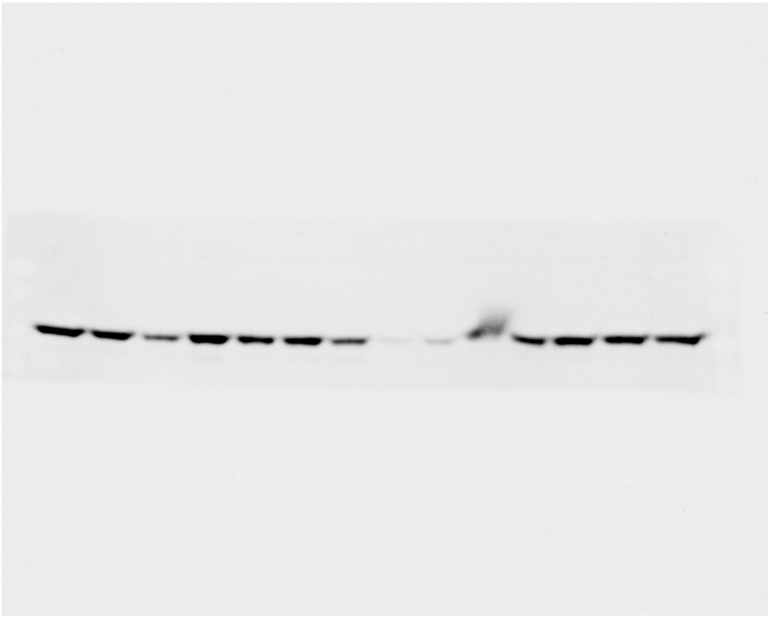

WB total LRRK2, total aSyn in WT LRRK2 cells (Fig S3) and Rit2-KO cells (Fig S9)

WB 12-10-22

Total LRRK2

Beta-actin

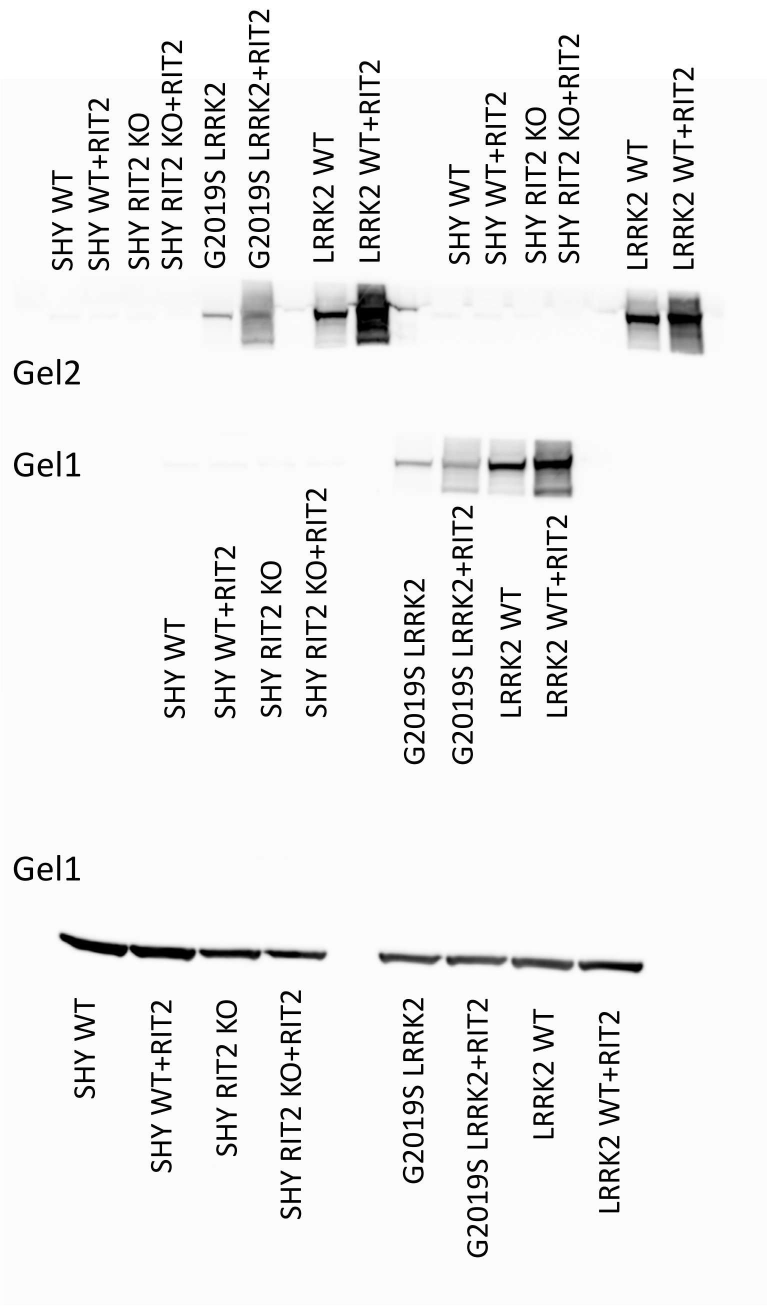

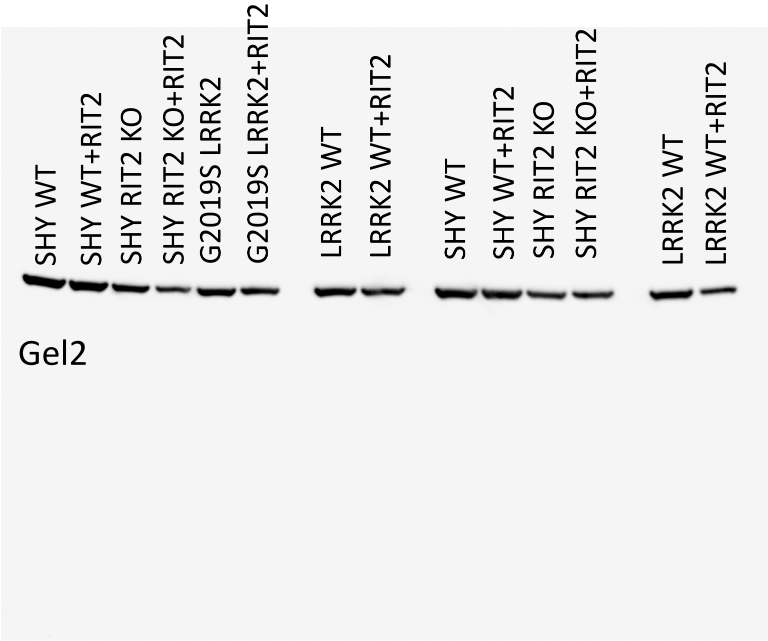

WB Rit2 overexpression G2019S LRRK2 (Fig S1), WT LRRK2 (Fig S3), Rit2-KO (Fig S9)

WB 10-05-2022

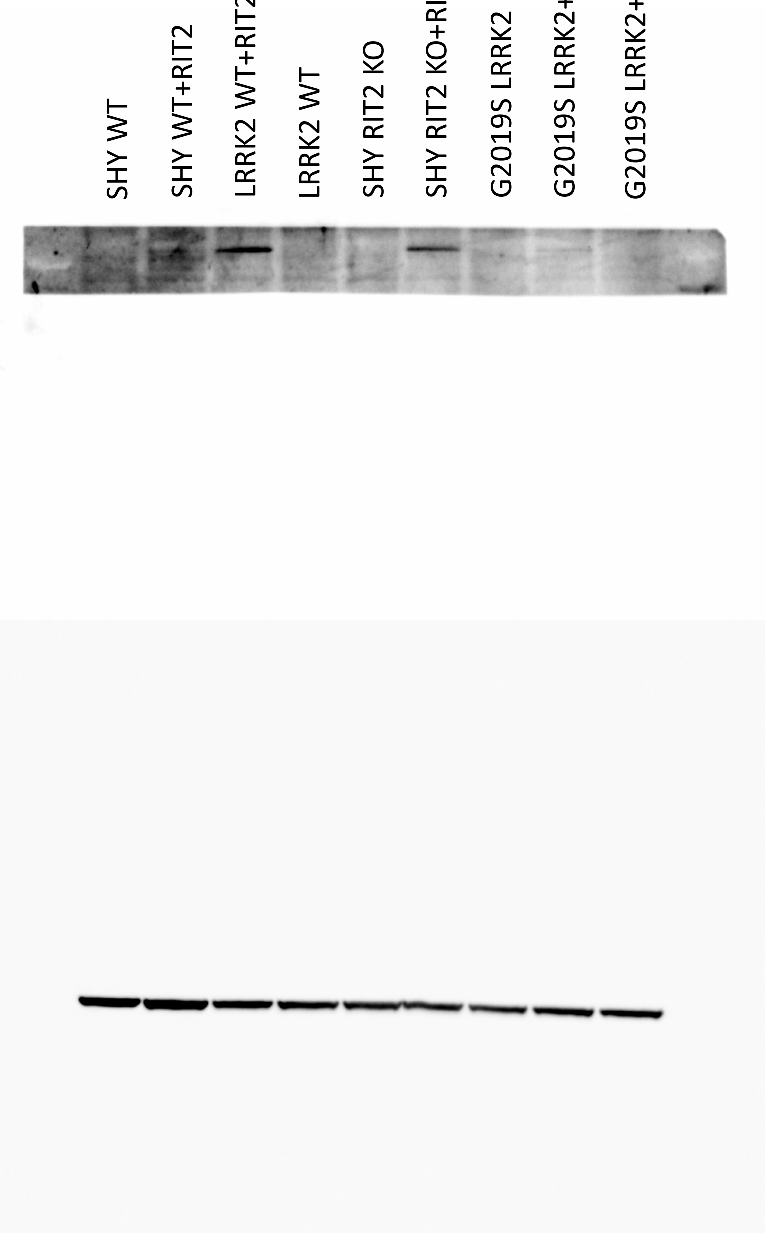

WB Rit2 overexpression G2019S LRRK2 (Fig S1), WT LRRK2 (Fig S3), Rit2-KO (Fig S9)

WB 26-01-2023

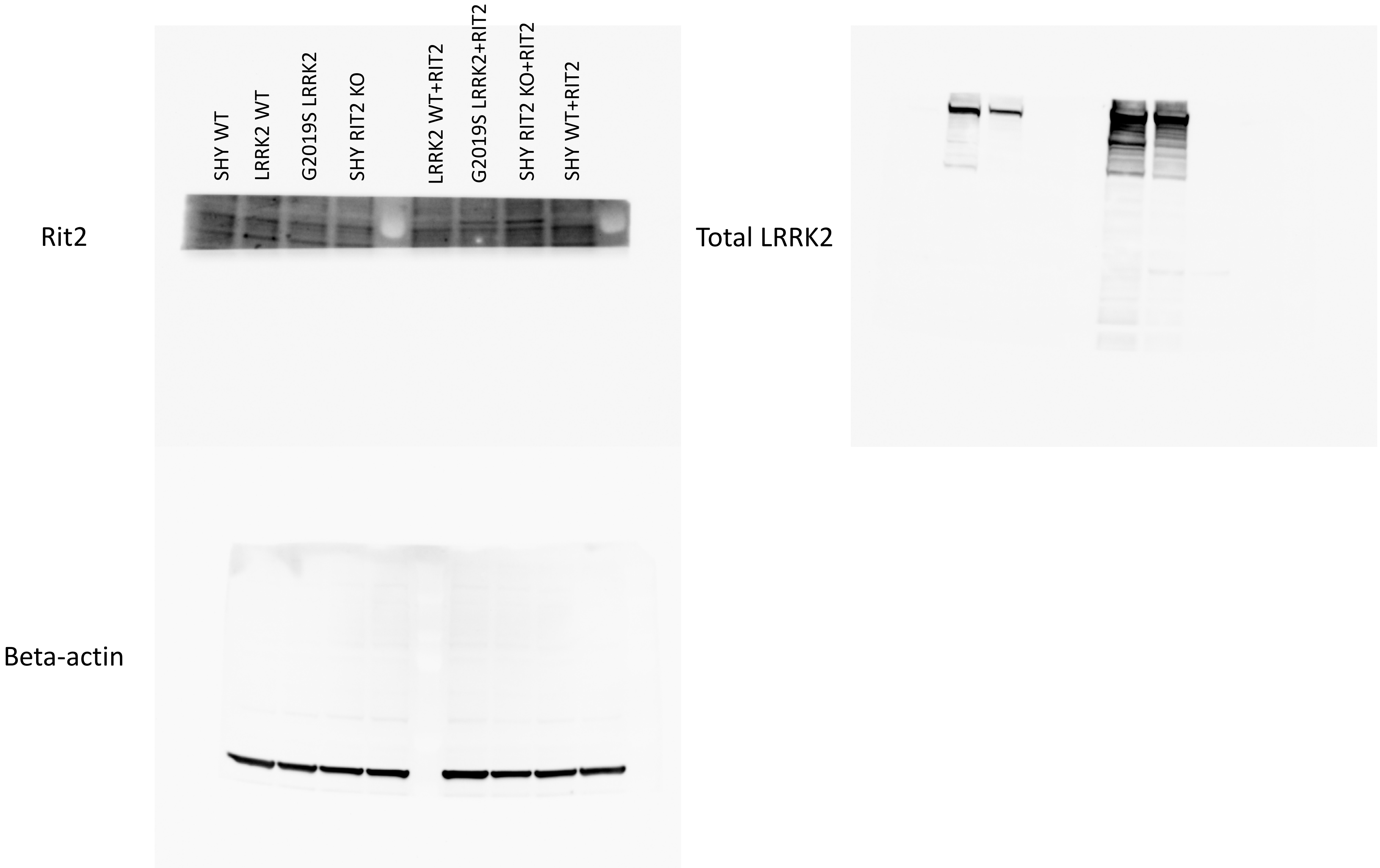

WB Rit2 overexpression G2019S LRRK2 (Fig S1), WT LRRK2 (Fig S3), Rit2-KO (Fig S9)

WB 30-01-2023

Rit2

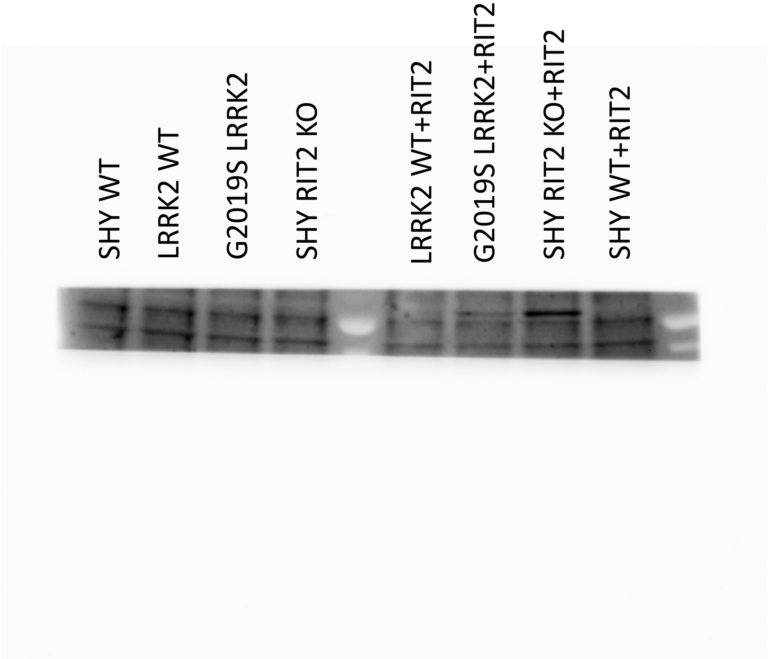

Total LRRK2

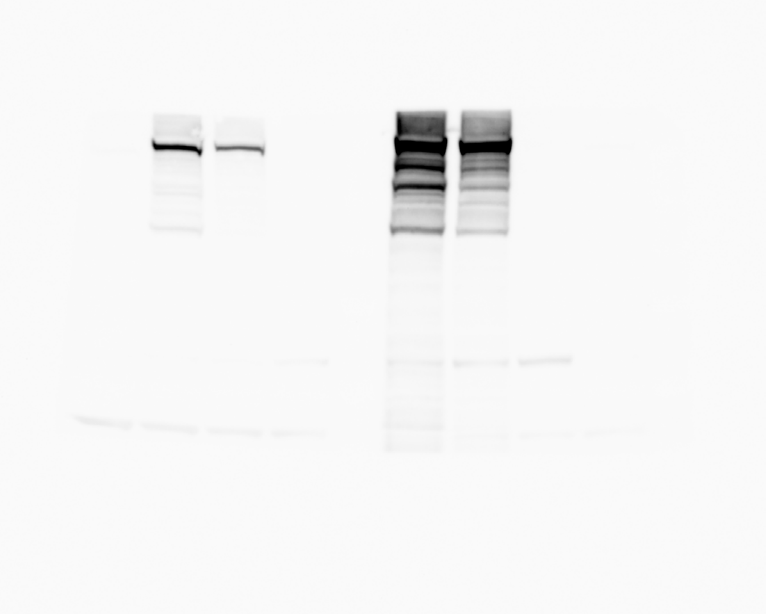

Beta-actin

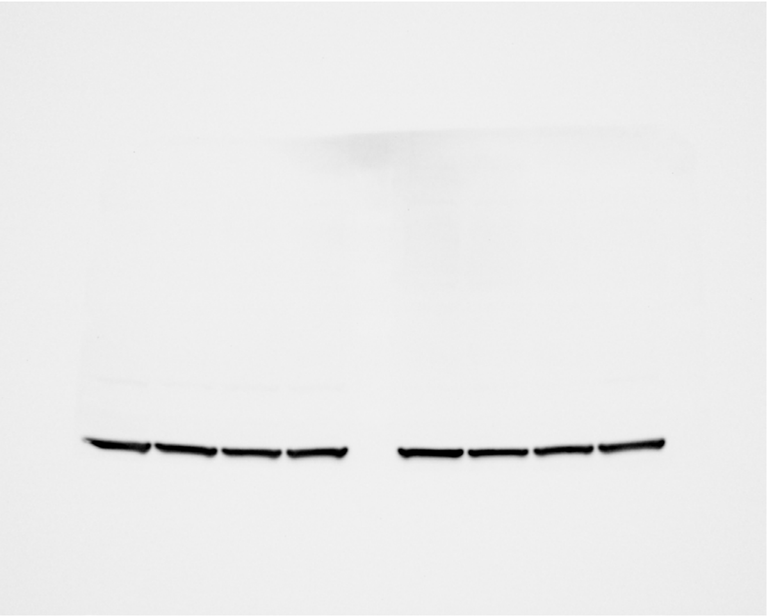

WB Rit2 overexpression G2019S LRRK2 (Fig S1), WT LRRK2 (Fig S3), Rit2-KO (Fig S9)

WB 02-02-2023

Rit2

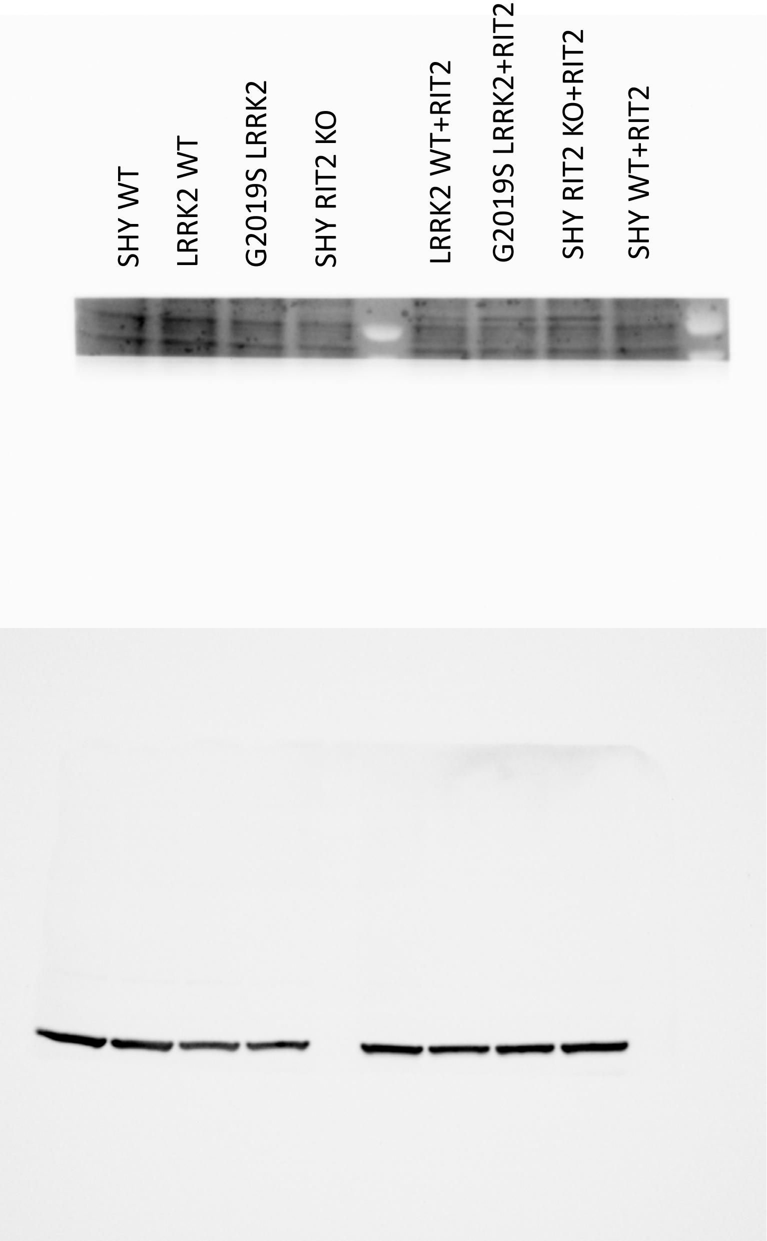

Beta-actin

Total LRRK2

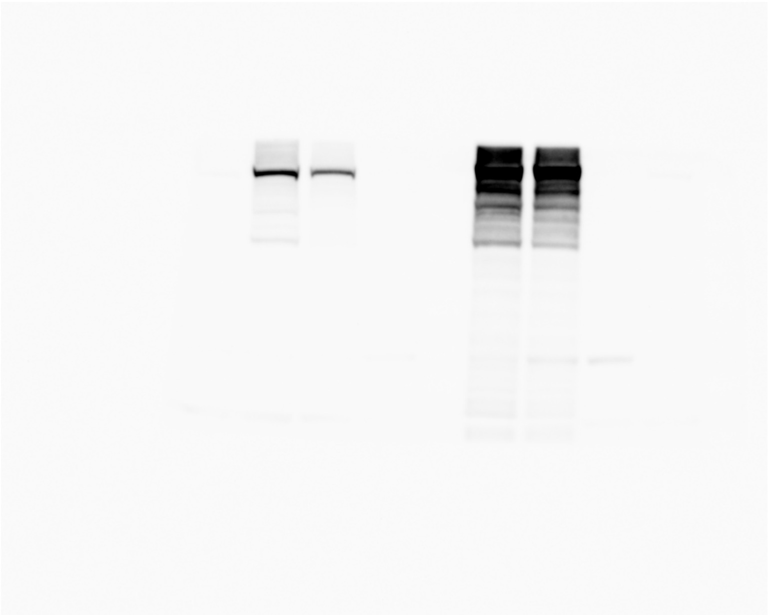

WB Rit2 overexpression G2019S LRRK2 (Fig S1), WT LRRK2 (Fig S3), Rit2-KO (Fig S9)

WB 06-02-2023

Rit2

Beta-actin

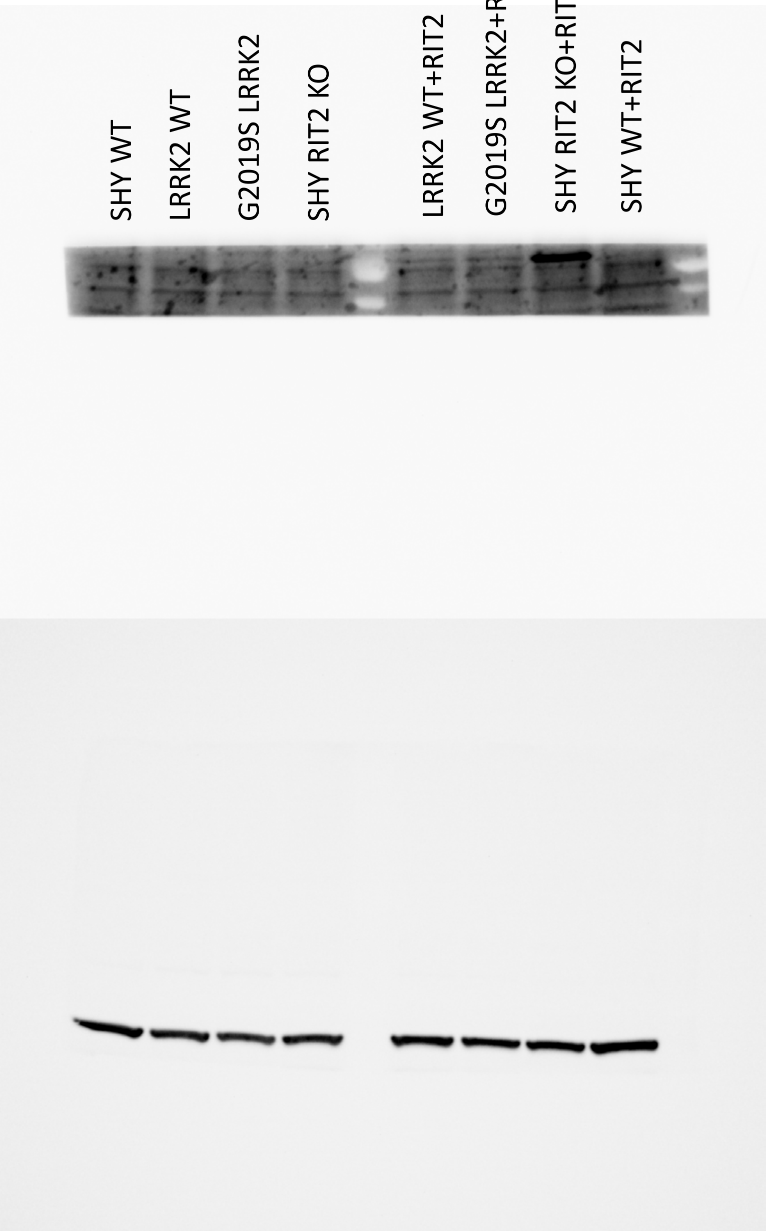

Total LRRK2

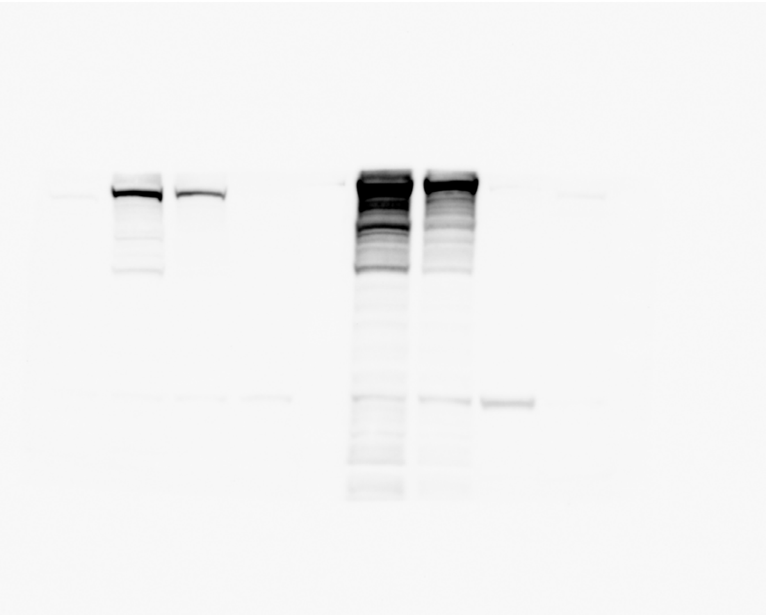

WB LC3B in Rit2-KO cells (Fig S9)

WB 05-05-2022

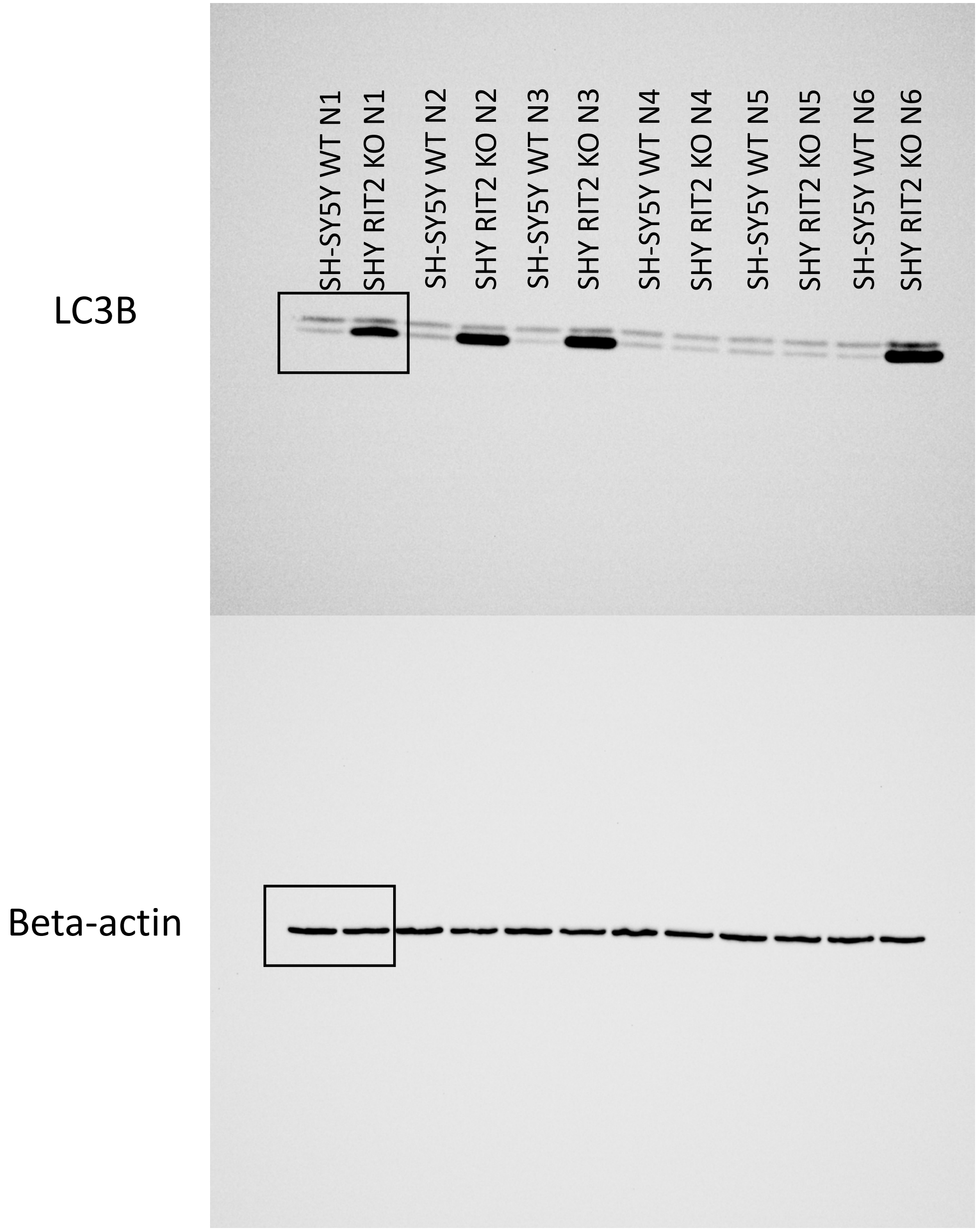

WB for Myc in NIH-3T3 cells (Fig S10)

Myc

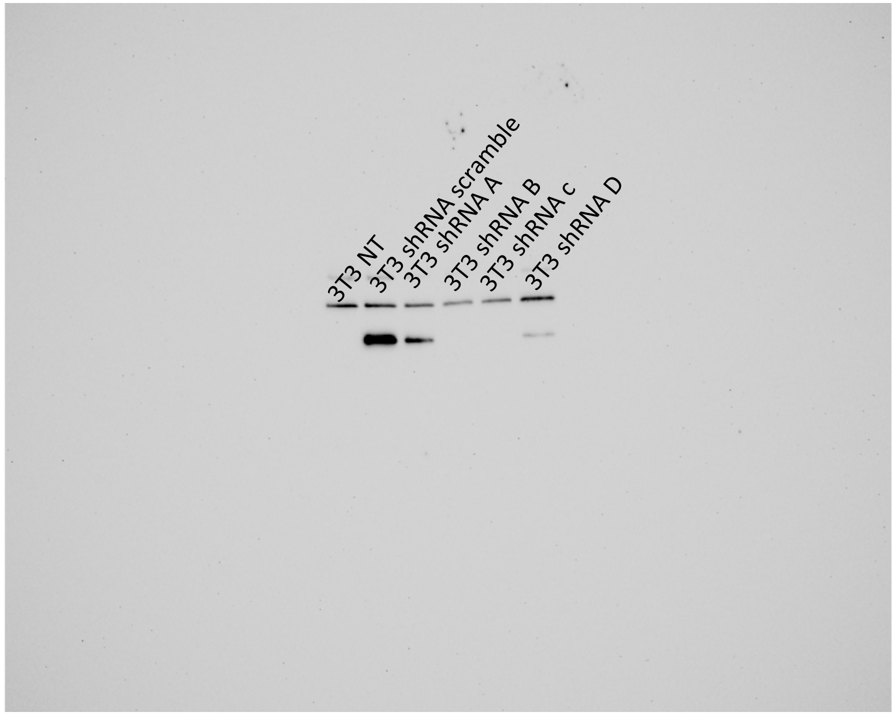

actin

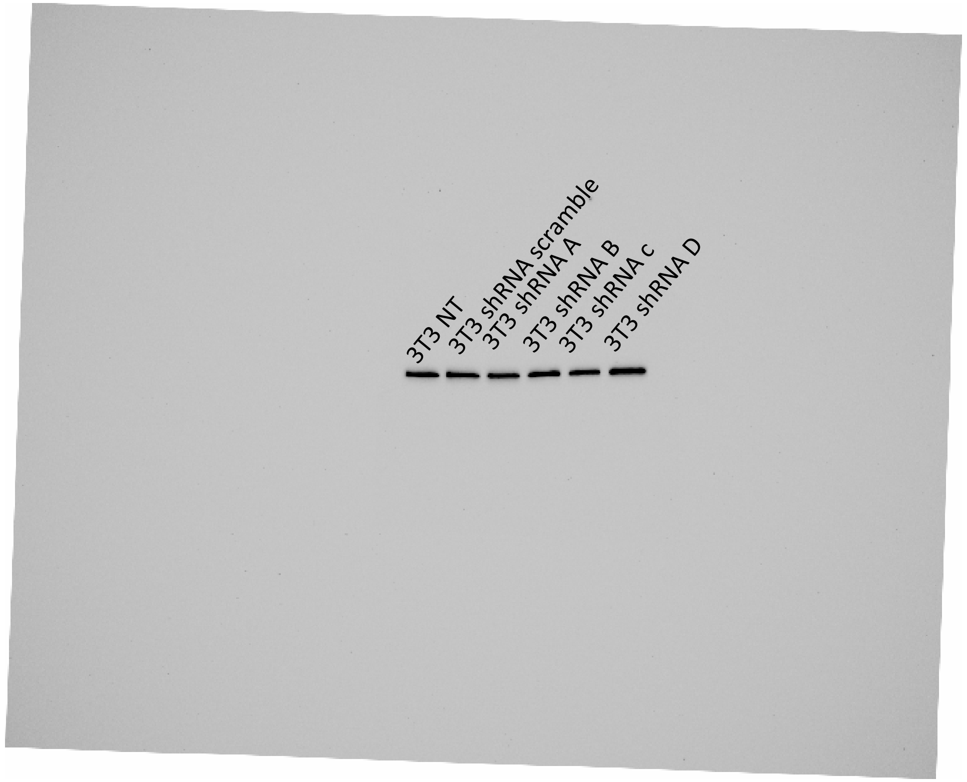

WB for LRRK2, Rit2, LC3B, TH in primary neurons (Fig S10)

TH

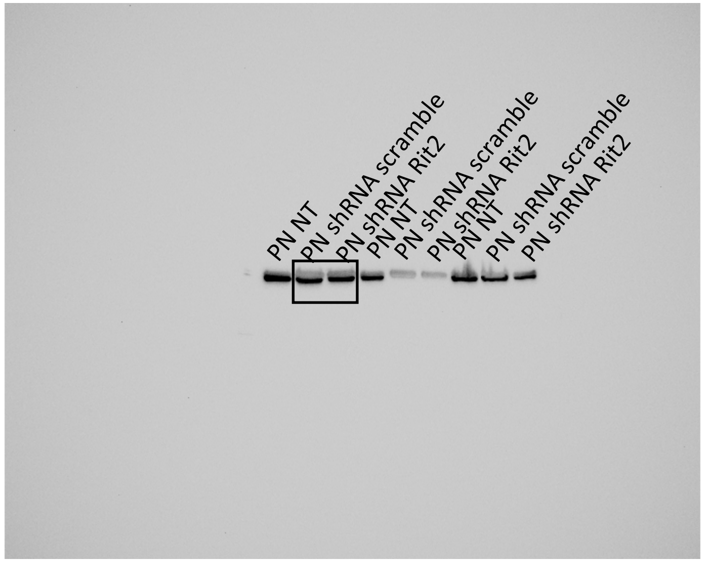

Rit2

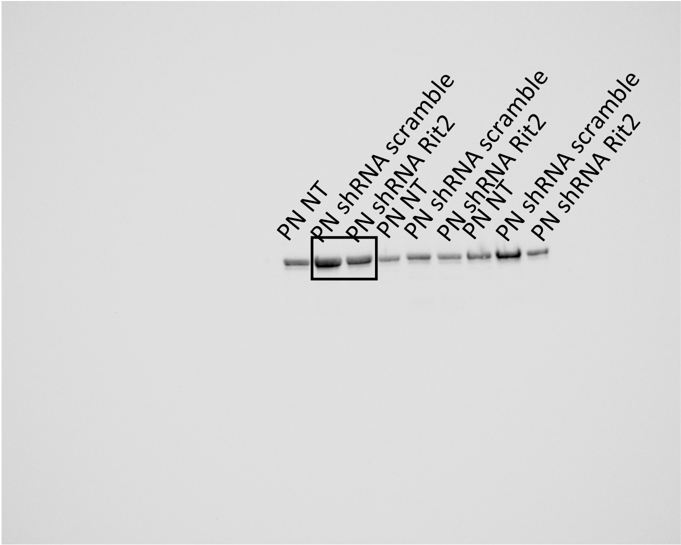

LC3B

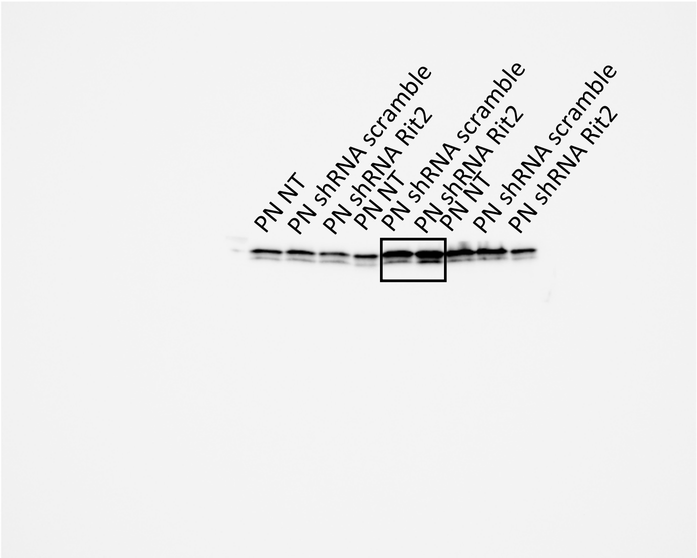

actin

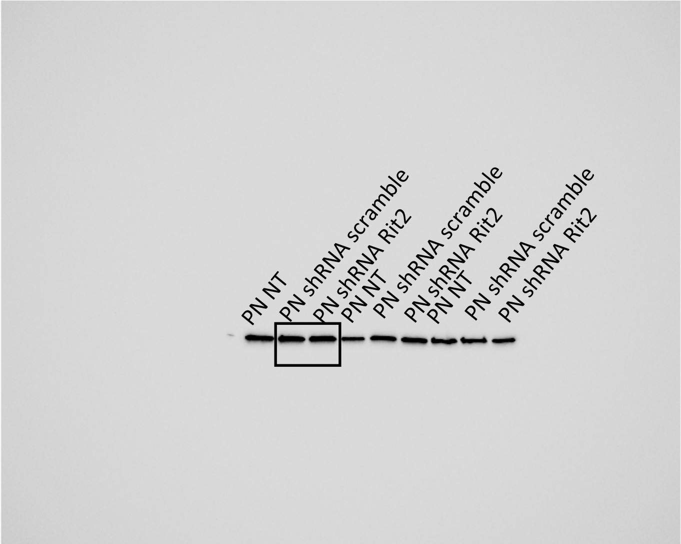

WB for LRRK2, Rit2, LC3B, TH in primary neurons (Fig S10)

Rit2

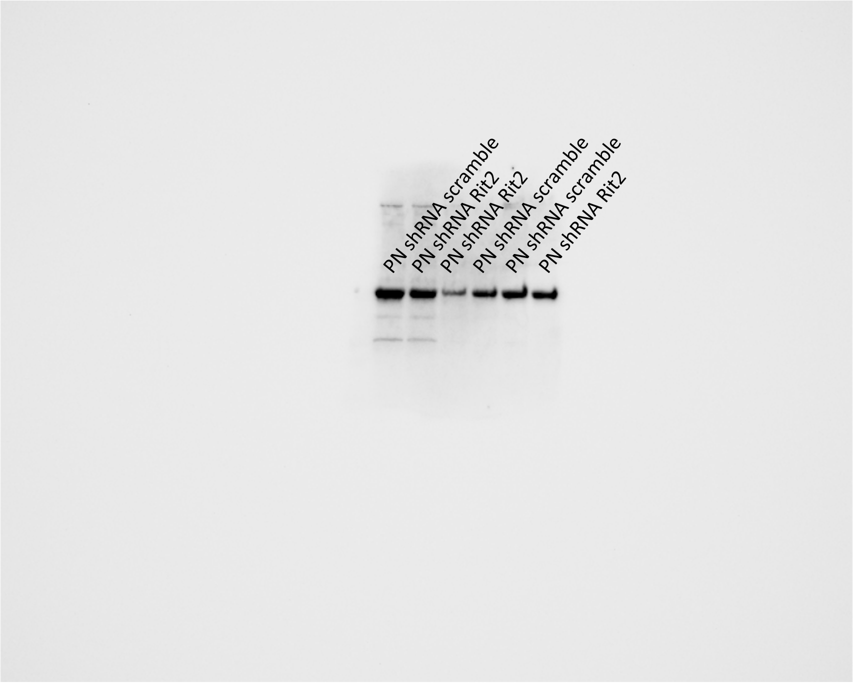

actin

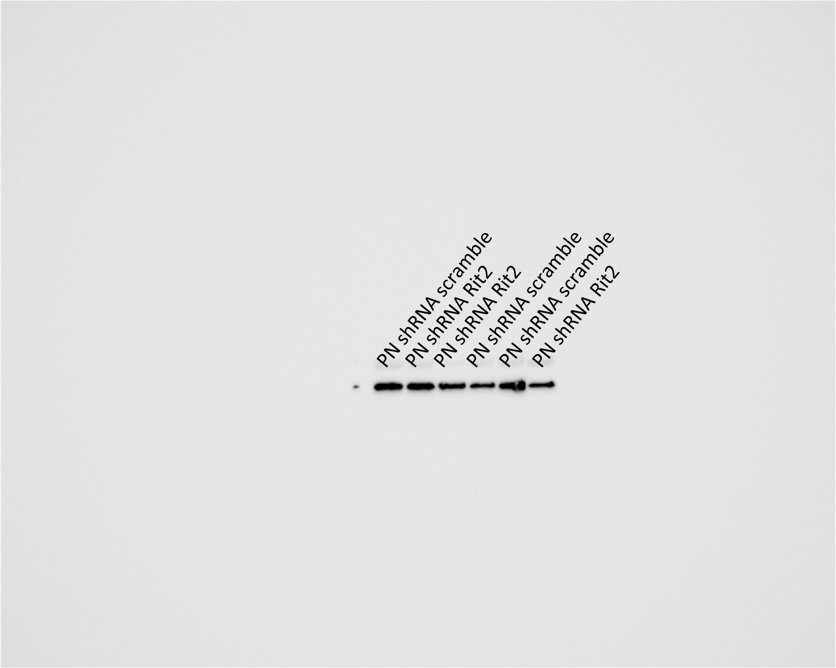

Supplement: Supplementary file 1 — Supplementary figures and legends [file 41531_2023_484_MOESM1_ESM.pdf]
